# Wealth redistribution policies to mitigate the impact of development assistance defunding: a retrospective evaluation and forecasting analysis up to 2030

**DOI:** 10.1016/S0140-6736(26)00975-X

**Published:** 2026-10-03

**Authors:** Gonzalo Barreix Sibils, Rodrigo Volmir Rezende Anderle, Andrea Ferreira da Silva, Lucas de Oliveira Ferreira de Sales, Daiana Pena, Caterina Monti, Claudia Garcia Vaz, Lucio Esposito, Edward Anderson, Xavier Jara, Hugo Santa, Gabriela Dos Santos de Jesus, Daniella Medeiros Cavalcanti, Ariel Nhacolo, Ivalda Macicame, Quique Bassat, Davide Rasella

**Affiliations:** a ISGlobal, Barcelona, Spain; b Facultat de Medicina i Ciències de la Salut, Universitat de Barcelona (UB), Barcelona, Spain; c Institute of Collective Health, Federal University of Bahia, Bahia, Brazil; d Universidade Regional do Cariri - URCA, Cariri, Brazil; e School of Global Development, University of East Anglia, Norwich, UK; f Universidad ICESI, Cali, Colombia; g London School of Economics and Political Science, London, UK; h Centro de Investigação em Saúde de Manhiça, Maputo, Mozambique; i Instituto Nacional de Saúde, Marracuene, Mozambique; j ICREA, Barcelona, Spain; k Institut Clínic de Medicina I Dermatologia, Hospital Clínic de Barcelona, Barcelona, Spain; l Pediatrics Department, Hospital Sant Joan de Déu, Universitat de Barcelona, Esplugues, Barcelona, Spain; m CIBER de Epidemiología y Salud Pública, Instituto de Salud Carlos III, Madrid, Spain

## Abstract

**Background:**

The ongoing decline in funding for humanitarian and development assistance stands in stark contrast to the global rise in wealth concentration and widening socioeconomic inequalities. This study aimed to assess whether the implementation of wealth redistribution policies—proposed in recent high-level international agreements to support development—could mitigate the health impact of the current official development assistance (ODA) defunding crisis.

**Methods:**

We conducted an integrated retrospective impact evaluation and forecasting analysis using longitudinal panel data from 59 low-income and lower-middle-income countries. First, we estimated the association between per-capita ODA and mortality outcomes from 2002 to 2021 using two-way fixed-effects multivariable Poisson regression models with robust standard errors, adjusting for key demographic, socioeconomic, and health-system covariates. We did extensive sensitivity and triangulation analyses to assess the robustness and plausibility of the findings. We then evaluated ten wealth redistribution policies with validated country-level microsimulation models to forecast mortality outcomes under alternative wealth redistribution scenarios versus the current defunding scenario from 2026 to 2030.

**Findings:**

In comparison with the baseline ODA category, high ODA funding was associated with a 24% reduction in age-standardised all-cause mortality (rate ratio [RR] 0·76; 95% CI 0·67–0·87) and a 33% reduction in under-5 mortality (0·67; 0·50–0·89). Forecasting analyses showed that, in comparison with the current ODA funding reductions in the 59 selected low-income and lower-middle-income countries, all ten wealth redistribution policies were associated with millions of averted deaths by 2030: overall, the lowest number of averted deaths was 6 591 984 (95% uncertainty interval 4 884 075–8 374 138) for a 3% crypto tax applied to the top 10 000 holders, while the highest number of averted deaths was 29 509 934 (20 273 842–38 603 961) for a 3% wealth tax on ultra-high-net-worth individuals; in children younger than 5 years, the corresponding numbers of averted deaths were 1 312 912 (802 926–1 785 121) for a 3% crypto tax applied to the top 10 000 holders and 3 464 607 (1 235 930–5 609 331) for a 3% wealth tax on ultra-high-net-worth individuals.

**Interpretation:**

The implementation of wealth redistribution policies proposed in recent high-level international agreements could mitigate the health consequences of the current ODA defunding crisis, avert millions of deaths, and support the achievement of the UN Sustainable Development Goals, particularly those related to health.

**Funding:**

Spanish Ministry of Science and Innovation.

## Introduction

The world is experiencing extreme levels of inequality, which is manifested across multiple dimensions and has worsened in the aftermath of the COVID-19 pandemic.^1^ Today, the top 10% of the global population owns approximately 75% of global wealth, while the bottom 50% holds only 2%, with absolute income inequality steadily increasing over the past three decades.^2^ Moreover, the wealthiest 0·002% of the global population—corresponding to 92 140 centi-millionaires—controls an estimated US$37·1 trillion in global wealth, surpassing the gross domestic product (GDP) of the world's largest economy—the USA ($31·1 trillion). In parallel, the world's 3028 billionaires collectively hold $16·1 trillion in wealth, and this extreme concentration of wealth is continuing to accelerate.^1^, ^3^ In stark contrast, the world's poorest populations are facing one of the most challenging periods in recent decades. Poverty rates have increased after the COVID-19 pandemic, with an estimated 808 million people living in extreme poverty (below $3 per day) and 3·5 billion people living in poverty (below $6·85 per day) in 2025.^4^ Recent estimates show that a quarter of low-income and middle-income countries (LMICs) still have per-capita incomes below 2019 levels, with the worst stagnation in low-income countries, where the COVID-19 pandemic erased all previous development gains.^5^

**Research in context**

**Evidence before this study**

We searched PubMed for reports examining the effects of official development assistance (ODA) on mortality worldwide, from database inception to Dec 31, 2025. The search was conducted in two streams: one broadly addressed ODA and the other explicitly focused on humanitarian assistance, to capture studies that might not have used ODA-specific descriptors. The ODA search string comprised the following descriptors: “(“international cooperation” OR “international funding” OR “international investment*” OR “foreign investment*” OR “global health investment*” OR “global health funding” OR “international aid” OR “official development assistance” OR “foreign aid” OR “foreign assistance” OR “development aid” OR “ODA” OR “development assistance for health”) AND (“mortality” OR “life expectancy” OR “death*” OR “fatal*”) AND (“program evaluation” OR “outcome assessment” OR “impact evaluation” OR “health effect” OR “causal effect” OR “effectiveness” OR “evaluation” OR “modelling”)”. An analogous search strategy was applied for humanitarian assistance. Searches were conducted in titles, abstracts, and keywords. Of 399 records retrieved, 74 met the inclusion criteria, and after abstract screening, 12 were identified as directly relevant to the research question. Across the reviewed literature, the effectiveness and impact of international cooperation and ODA on mortality have been evaluated through various modelling studies, primarily programme-specific models of US President's Emergency Plan for AIDS Relief funding disruptions that show substantial excess deaths among children, mothers, and those with HIV when funding is halted or frozen. Country-level longitudinal research further links child-health ODA to lower infant and under-5 mortality rates, while effects on maternal and neonatal outcomes are mixed and seem to depend on subsector composition, funding stability, and coordination. Overall, the evidence suggests that targeted, stable ODA contributes to mortality reduction, whereas sudden cuts or aid sanctions are associated with increased deaths and service coverage losses. Despite this evidence, most studies have concentrated on models or US-funded programmes, leaving the effects of overall ODA, including differences among other donors, on mortality less understood. There is a need for research that combines retrospective data and projections across a broader set of countries, especially those most vulnerable to ODA shocks, to fully understand the effects of funding fluctuations on mortality. Regarding wealth redistribution policies, we searched PubMed for reports examining the effects of wealth redistribution policies on mortality worldwide, from database inception to Dec 31, 2025. The search string comprised the following descriptors: “(“wealth” “assets” OR “net worth” OR “capital” OR “property”) AND (“tax*” OR “redistribution” OR “fiscal policy” OR “fiscal policy” OR “levy”) AND (“mortality” OR “life expectancy” OR “death”)”. Of 117 records retrieved, 12 met the inclusion criteria, and after abstract screening, two were identified as directly relevant to the research question. Although there is a substantial literature on the relationship between wealth levels and mortality, evidence on the effects of wealth redistribution policies remains scarce. To date, only one recent study has evaluated these policies, showing that—when applied to the US context—they could substantially reduce survival inequities and increase population longevity.

**Added value of this study**

This study combines retrospective analyses with near-term projections to 2030 across a broad set of countries, with a particular focus on low-income and lower-middle-income countries, which are most vulnerable to fluctuations in international assistance. This approach allows us to comprehensively assess the effects of international cooperation—and its potential contraction—on mortality in the context of the ongoing ODA crisis. Our models estimate that higher ODA funding levels over the past two decades were associated with a 24% reduction in age-standardised all-cause mortality and a 33% reduction in under-5 mortality. We further project that, if current ODA defunding trends continue, more than 7·9 million additional deaths could occur in low-income and lower-middle-income countries by 2030. Crucially, our study provides the first evidence that implementing wealth redistribution policies proposed in recent high-level international agreements to support development could mitigate these health impacts, potentially saving between 6·6 million and 29·5 million lives by 2030, depending on policy type, target population, and taxation level.

**Implications of all the available evidence**

The current sudden and severe reductions in ODA funding could have serious consequences for population health. However, our findings demonstrate the substantial potential of alternative wealth redistribution policies—such as those already proposed in recent high-level international agreements on global development—to mitigate the health benefits lost due to ODA defunding. If implemented, such policies could save millions of lives and substantially accelerate progress towards achieving the health-related UN Sustainable Development Goals by 2030.

In this context, the recent substantial reductions in funding for humanitarian and development assistance—commonly referred to as official development assistance (ODA)—by several donor high-income countries are further exacerbating global inequalities and having severe consequences for the world's most vulnerable populations.^6^, ^7^ ODA represents only a small fraction of donor countries’ gross national income, averaging just approximately 0·3%, and remains modest in absolute terms (around a few billion dollars for most donors).^8^, ^9^, ^10^ In 2023, total ODA amounted to $250·3 billion, with France, Germany, Japan, the UK, and the USA accounting for roughly 70% of global contributions.^9^, ^10^ Yet, all major donors reduced their ODA in 2024 and 2025, representing the first historical instance of these five countries reducing ODA simultaneously for two consecutive years.^8^, ^9^, ^10^ The dismantling of the US Agency for International Development (USAID) in July, 2025, accompanied by an announced 83% reduction in its programmes, illustrates the scale of the crisis.^6^ Outlooks beyond 2025 remain highly uncertain, as budgets for 2026 and 2027 have been announced, although in some donor countries they have not yet been formally approved and budgeting cycles vary considerably across Development Assistance Committee (DAC) members.^8^, ^9^, ^10^ However, preliminary projections point to further substantial reductions from major DAC donors, amounting to an estimated overall decline of 7·0% from 2025 to 2026,^9^ and resulting in estimated reductions in ODA funding by 2026—relative to 2023 levels—of 58·5% for the USA, 41·4% for the UK, 34·8% for Germany, 16·2% for France, and similarly large decreases for nearly all other donor countries, with only a few exceptions.

Recent studies have shown that humanitarian and development assistance has had a substantial impact on the health of the most vulnerable populations over the past few decades. Projections have examined the potential consequences of defunding such assistance in 133 LMICs, estimating 14·1 million deaths by 2030—including 4·5 million deaths in children younger than 5 years—resulting from near-complete dismantling of USAID programmes.^6^ Another study comprising 93 LMICs estimated that reducing ODA to minimal levels could result in 22·6 million excess deaths by 2030.^11^ Focusing on a smaller set of LMICs, another study estimated that eliminating US foreign assistance for health could result in more than 4 million additional AIDS deaths between 2025 and 2030, more than 600 000 tuberculosis deaths, and 2·5 million child deaths, alongside millions of unintended pregnancies and unsafe abortions.^12^ Recent estimates suggest that a 30% long-term reduction in humanitarian and development funding, in particular related to health care, could be responsible for 16 million excess child deaths by 2045.^13^ For most LMICs, especially the most vulnerable, no effective short-term mitigation strategies exist to cope with the abrupt reduction of ODA funding. Although some countries have attempted to reallocate domestic resources to partially offset external funding losses, these efforts have been few and modest, falling well short of compensating for the scale of ODA cuts.^14^, ^15^ This shortfall reflects the structural constraints faced by countries that are most dependent on humanitarian assistance, including fragile health-care and social protection systems and high public debt burdens.^1^, ^7^, ^10^

To support development financing and reduce global inequalities, wealth redistribution policies have been endorsed in recent high-level international declarations, including the Rio de Janeiro G20 Leaders’ Declaration of November, 2024,^16^ the Sevilla Commitment of the UN Fourth International Conference on Financing for Development (FfD4) in June, 2025,^17^ and the associated Sevilla Platform for Action,^18^ among others. In this context, a joint initiative promoted by the Brazilian and Spanish Governments under the Seville Platform for Action has been launched to advance a framework for effective taxation of high-net-worth individuals,^19^ and 147 countries have agreed on a coordinated operation of global minimum tax arrangements within the Organisation for Economic Co-operation and Development (OECD)/G20 Inclusive Framework on Base Erosion and Profit Shifting (BEPS), which includes measures such as the global minimum tax on multinational corporate profits, ensuring a minimum effective tax rate of 15% on the profits of corporations with revenues exceeding €750 million.^20^ Even highly complex domains such as the cryptocurrency sector are undergoing considerable reforms enabling the taxation of digital assets (eg, the Crypto Asset Reporting Framework, undersigned by >60 jurisdictions).^21^ All of these wealth redistribution policies could mobilise substantial resources. For example, a report commissioned by the EU estimates that a 2% tax on the wealth of high-net-worth individuals could generate up to $250 billion per year,^22^ while other estimates suggest that a 5% tax on the wealth of the more than 92 140 centi-millionaires could raise up to $1·3 trillion annually.^1^ According to these declarations and related action frameworks, the proposed taxes could be collected either at the national level or through cross-border mechanisms,^16^, ^17^, ^18^, ^19^, ^22^ such as the Global Solidarity Levies Task Force, and are intended to support inequality reductions and global development, including through bilateral or multilateral ODA.

To the best of our knowledge, no studies have comprehensively evaluated the global health impact of wealth redistribution policies, nor has any work estimated the potential health effects of transferring wealth from the world's richest individuals to the poorest on a global scale. This study aimed to assess the extent to which the implementation of a range of wealth, income, and socially efficient taxation policies—as proposed in recent high-level international agreements to support global development—could mitigate the mortality impact of the current defunding trends in humanitarian and development assistance. Specifically, we comprehensively evaluated the impact of ODA on adult and child mortality in low-income and lower-middle-income countries over the past two decades; estimated the mortality consequences of current defunding trajectories through 2030; and quantified the mitigation potential of selected wealth redistribution strategies by estimating the number of lives saved under each approach.

## Methods

### Study design

We conducted a retrospective impact evaluation (ex-post, covering the period 2002–21) integrated with forecasting analyses (ex-ante, projecting from 2026 to 2030), both using the same data structure and analytical models. We applied the same methodological approach and study design used in our recent study, which evaluated the impact of USAID defunding on mortality in LMICs.^6^

The study used a longitudinal ecological design in which countries as units of analysis were retrospectively observed and projected until 2030. The dataset included publicly available, aggregated demographic, socioeconomic, health, and ODA data from 2002–21 (appendix p 9). Among all countries and territories globally, only the most vulnerable—defined as low-income and lower-middle-income countries—were included, totalling 59 countries and representing 3·9 billion people in 2021 (appendix p 7).

### Study variables

The primary outcome was age-standardised all-cause mortality rate (ASMR) per 1000 people and under-5 child mortality rate (U5MR) per 1000 livebirths. We also analysed different age subgroups in children: infancy (0–1 year), preschool (2–4 years), child (<5 years), and school age (5–9 years).

Our exposure variable was the financial assistance provided by OECD-defined official donors in social infrastructure and services, and multisector or cross-cutting and humanitarian aid sectors, specifically measured as ODA funding per capita. These sectors were chosen as they express health determinants that are expected to affect mortality in the short term.^6^, ^7^, ^12^, ^23^, ^24^, ^25^ Other subsets were explored in sensitivity analyses. The ODA funding per capita was calculated following previous studies,^26^, ^27^ by dividing the total disbursed amount by the total population for each of the 59 countries across each fiscal year from 2002 to 2021. Following previous research,^28^, ^29^ we categorised the per-capita ODA funding to take into account the non-linear dose–response relationship resulting from different intervention levels. Given the absence of established reference values in the literature and in line with previous evaluations,^6^, ^28^, ^29^ we categorised per-capita ODA funding using quintiles of its distribution: baseline, considered as not exposed ($0–12·34 per capita per year); low ($12·35–24·40); lower-intermediate ($24·41–35·97); upper-intermediate ($35·98–52·12); and high ($52·13 and higher). We preferred this over a continuous specification due to the uncertainty about the dose–response functional form and to reduce disturbances in effect estimates due to outliers originating from continuous models. To check the robustness of these assumptions, we tested specifications of continuous variables and multiple alternative categorisations. All the results remained robust (appendix p 19).

All relevant time-variant adjusting variables according to the literature,^26^, ^27^, ^30^, ^31^ including demographic, socioeconomic, and health-care variables, were included in the model specification: economic downturns, GDP per capita at purchasing power parity (PPP), armed conflict or wars index, fertility rate, percentage of urban population, percentage of primary school attendance, percentage of people using at least basic drinking water services, health expenditure per capita (at PPP), and percentage of social protection coverage. As in previous studies,^6^, ^28^, ^29^ to harmonise the model and reduce disturbances due to potential outliers, covariates were dichotomised according to their median value over the period (except for economic downturn and armed conflict or wars index; appendix p 10). We also added time dummy variables for the years 2009, 2015, 2020, and 2021 from the Global Health Data Exchange to account for major global economic and health shocks over the past 20 years.

ASMR comes from the Global Burden of Disease Collaborative Network. Data on ODA funding were collected from the OECD Creditor Reporting System, flow-based ODA. Covariate variables were collected from the World Bank data and the WHO Global Health Observatory data (appendix p 9).

### Statistical analysis

In the retrospective analysis, we estimated the effect of ODA per capita on ASMR (and age-specific mortality rates) during the period 2002–21, using Poisson multivariable regression models with robust standard errors and two-way fixed-effects specifications. This consolidated methodological framework, supported by extensive sensitivity checks and triangulation analyses, allows for the estimation of intervention effects on mortality using aggregate-level panel data.^6^, ^28^, ^29^, ^30^, ^32^ The two-way fixed-effects models have the advantage of including a term to control for unobserved non-time-varying characteristics of the unit of analysis, such as the geographical, historical, infrastructural, or sociocultural aspects of each country, which could be associated both with the outcome and with the intervention implementation; and time dummy variables to control for unobserved variables related to yearly social, economic, and health shocks that affected the countries under study.^28^, ^29^, ^30^ We also generated a stratification analysis by countries’ governance levels in order to conduct the forecasting analysis.

The stratified analyses of ODA effectiveness according to governance levels were conducted by classifying countries into two strata—worst-off and better-off governance conditions. To construct this indicator, we used the World Bank's Worldwide Governance Indicators, which include six dimensions: voice and accountability; political stability; government effectiveness; regulatory quality; rule of law; and control of corruption.^33^ For each country–year dyad, we calculated the average score across the six dimensions to obtain a composite governance indicator. We then dichotomised this indicator into two categories based on the median value of the distribution, resulting in the two governance groups used in the stratified analyses.

A wide range of sensitivity analyses were done for the retrospective evaluation. First, to assess the sensitivity of the results to the exposure specification, we re-estimated the models using continuous measures of ODA per capita and alternative categorical groupings, varying both the number of categories and the threshold values. Second, to evaluate the external validity of our findings—that is, to test whether the observed associations held across a broader global context—we expanded the sample to include more countries and territories worldwide. Third, to examine the potential influence of temporal dynamics, we re-specified the models using alternative sets of time variables to capture different temporal structures, and we conducted different time-dynamic ODA effect analyses: lag, duration, and consolidation effects. Fourth, to verify the specificity of the models, we conducted a negative control analysis using mortality due to transit injuries. Fifth, to better characterise ODA effectiveness, we analysed other subsets of ODA assistance sectors, and the same ODA sector subsets but with only DAC country member donors. Finally, to validate the overall impact evaluation, we conducted triangulation analyses—specifically, difference-in-differences supplemented by propensity score matching—and applied Bradford Hill criteria for causal inference.

All data processing and statistical retrospective analyses were done with Stata (version 17.0).

In the forecasting analyses, we used validated country-level microsimulation models to project the mortality impacts of the current ODA defunding crisis until 2030. Microsimulation is one of the most rigorous and accurate forecasting techniques, as it enables the integration of country-specific characteristics and their associated outcome probabilities directly into the modelling framework. This approach is particularly robust when model parameters are derived from retrospective, real-world cohorts, as it preserves the empirical distribution of variables, their intercorrelations, and country-level temporal dynamics.^31^ Building on established methodologies applied in previous studies,^6^, ^29^, ^32^, ^34^ our modelling framework comprised two main stages. In the first stage, we generated a synthetic cohort including all countries for the period 2021–30 (including the years before 2026 for calibration and validation purposes) based on the extrapolation of each country-level independent variable extended from the retrospective dataset, divided into two cohorts according to governance levels. In the second stage, we used these projected inputs in two multivariable Poisson regression models—one for each governance stratum—retaining the same variables and specifications as in the stratified retrospective analyses, allowing ODA effects and other independent variables to vary by governance conditions, and including country fixed effects.

Various ODA funding scenarios were simulated. First, we generated the current defunding scenario, defined as the ODA resulting from the current defunding data and trends from 2024 to 2030: starting with ODA data from 2023 (the last year with consolidated country-specific data), we then applied the already estimated defunding rate of 6·33% to compute the ODA for 2024,^7^, ^9^, ^10^, ^11^ and then used the estimated 15·8% defunding rate from 2024 to obtain the 2025 ODA.^9^, ^11^ For the following years, we applied an annual decrease of 11·1%, the mean of the decrease in the period from 2023 to 2025, until 2030.^7^, ^9^, ^10^, ^11^ In the 59 low-income and lower-middle-income countries, for the three selected ODA sectors under study, the ODA overall value corresponded to $76·7 billion in 2023, and reached $33·6 billion by 2030.^7^

We then estimated ASMR and U5MR up to 2030 according to this scenario and compared it with a scenario where the levels of ODA funding were maintained at a constant level over the same period (ie, business-as-usual scenario), based on the most recent country-specific data available (2023), which also corresponded to the historical funding peak.

Then, we generated different wealth redistribution scenarios, one for each funding source (and its associated funding level), in which—on the top of the current defunding scenario—the funds were added to the system. These total annual funds were introduced progressively, applying a bounded exponential function, simulating initial implementation challenges and rates of avoidance and evasion, beginning below the full allocation in 2026 and increasing each year at a diminishing increasing rate until the full annual amount is reached by 2030. For example, the $102·2 billion in carbon tax begin entering the system in 2026, with an initial inflow of $64·6 billion in the first year. This amount then increases each year—at a diminishing increasing rate—until reaching the full $102·2 billion by 2030. The share of total incoming funds allocated to each country in a given year was determined by that country's proportional share of ODA in the preceding year. This approach was used to keep each country's funding allocation as close as possible to the pre-defunding distribution. However, when a country attained the high ODA per capita level, no additional ODA was provided in the following year (appendix p 43).

For each outcome and scenario, we conducted 1000 Monte Carlo simulations, varying parameters across iterations based on their empirical probability distributions. Comparisons between scenarios focused on ASMR and U5MR, expressed as mortality rate ratios (RR) and absolute differences in deaths, including estimates of cumulative excess mortality over the entire 2026–30 period. All models were subjected to external validation.

Moreover, to verify the robustness of the forecasting results with alternative approaches, we developed country-specific algebraic simulations (appendix p 61).

All forecasting procedures adhered to international model reporting standards (guidelines from the Professional Society for Health Economics and Outcomes Research and Society for Medical Decision Making)^35^ and were implemented in R (version 4.1.2). Detailed documentation of the modelling process, including calibration, validation, parameter distributions for the Monte Carlo simulations, and full model equations is provided in the appendix (p 47).

### Role of the funding source

The funder had no role in study design, data collection, data analysis, data interpretation, or writing of the manuscript, or the decision to submit the manuscript for publication.

## Results

We calculated the mean values and trends of all the variables in the selected countries over the 2002–21 study period (figure; table 1). Overall ASMR in 2002 was 12·80 (SD 3·45) per 1000 population and decreased by 6·64% to 11·95 (2·74) per 1000 population in 2021, whereas U5MR was 89·68 (42·92) per 1000 livebirths in 2002 and decreased by 49·91% to 44·92 (25·27) per 1000 livebirths in 2021. On average, ODA funding increased by 108·60% (from $20·94 per capita to $43·68 per capita), whereas the average funding per country increased by 239·64% (from $323·74 million to $1099·55 million). The figure shows the ODA quintiles, overall ASMR, and U5MR between 2002 and 2021 by country, showing a general inverse relationship between them. Overall, socioeconomic conditions improved, in terms of GDP per capita, basic drinking water, and primary education. The percentage of the urban population, health expenditure per capita, and social protection coverage also increased. The conflict or wars index and fertility rate decreased, and the proportion of countries with economic downturns varied between 2002 and 2021.

**Figure:**
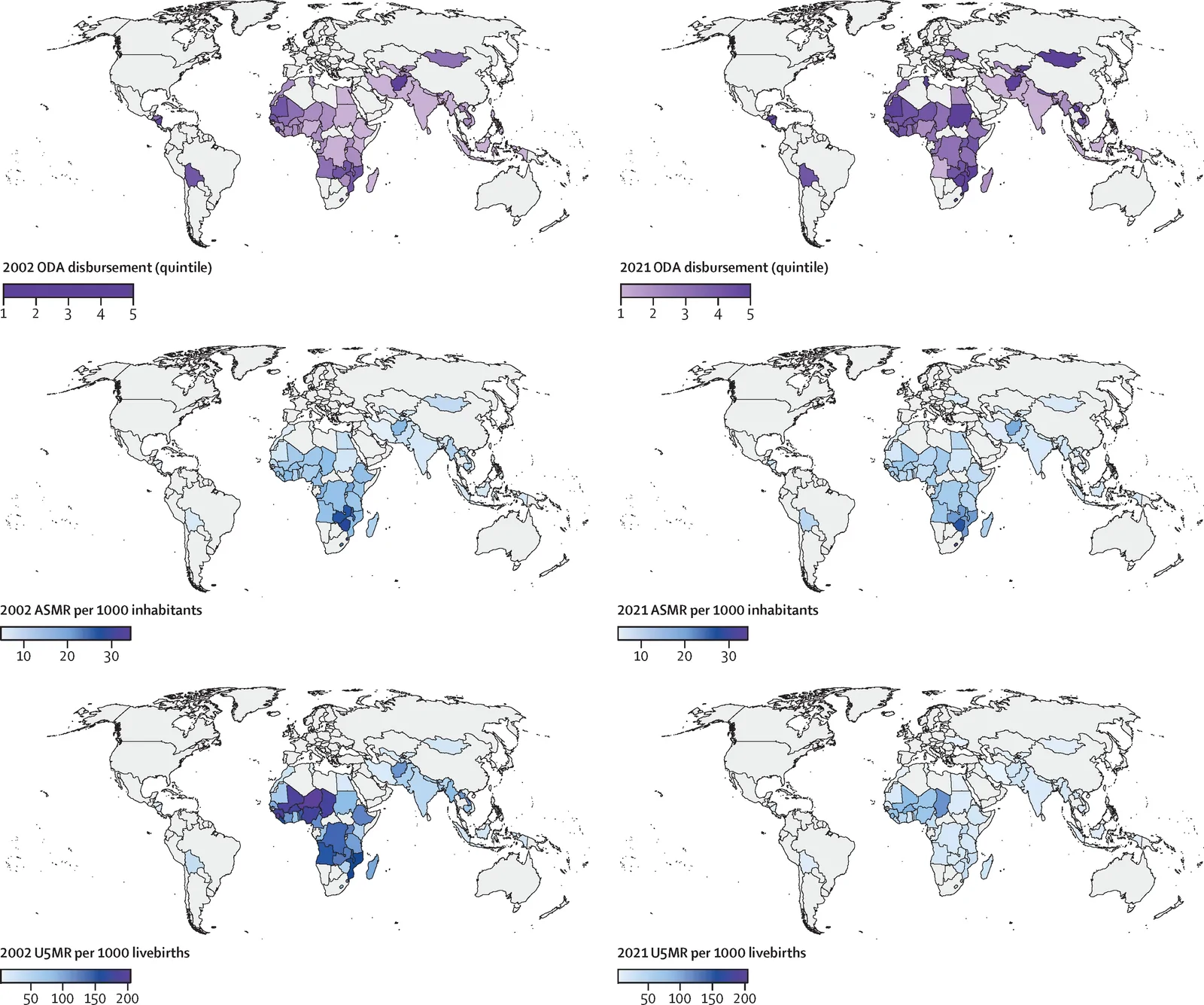
ODA funding per capita by quartiles across the selected 59 low-income and lower-middle-income countries in 2002 and 2021, age-standardised mortality rates per 1000 inhabitants in 2002 and 2021, and under-5 mortality rates per 1000 livebirths in 2002 and 2021 ASMR=age-standardised mortality rate. ODA=official development assistance. U5MR=under-5 child mortality rate.

**Table 1:** Descriptive statistics of mortality rates, ODA funding, and demographic, socioeconomic, and health-care-related variables in the selected countries (n=59) from 2002 to 2021

	**2002**	**2011**	**2021**	**Change from 2002 to 2021**
**Mortality rate**				
Overall ASMR per 1000 population	12·80 (3·45)	11·01 (2·36)	11·95 (2·74)	−6·64
Children aged <5 years per 1000 livebirths	89·68 (42·92)	68·50 (33·27)	44·92 (25·27)	−49·91
**ODA or funding**				
ODA per capita (constant US$)	20·94 (17·84)	41·29 (37·75)	43·68 (28·05)	108·60
Average funding per country (in millions of US$)	323·74 (339·57)	814·82 (943·92)	1099·55 (979·86)	239·64
**Population size**				
All 59 countries	2 726 214 458	3 295 823 882	3 897 063 090	42·95%
**Other covariates**				
Economic growth (%)	4·8% (6·39)	4·92% (4·23)	4·39% (4·94)	−8·54%
GDP per capita PPP (constant US$)	3761·46 (2423·56)	5220·56 (3673·93)	6237·61 (4218·8)	65·83
Conflict or wars index ξ	0·09 (0·2)	0·05 (0·13)	0·05 (0·12)	−44·44
Fertility rate (births per woman)	4·6 (1·59)	4·09 (1·53)	3·54 (1·31)	−23·04
Urban population (% population)	35·9% (15·22)	40·06% (16·36)	44·23% (16·85)	23·20%
Primary education (% population)	49·86% (25·09)	54·39% (24·84)	60·79% (25·73)	21·92%
Basic drinking water (% population)	62·00% (19·81)	69·69% (17·47)	77·25% (15·77)	24·60%
Health expenditure per capita (PPP)	114·93 (98·08)	206·5 (195·74)	321·45 (262·93)	179·69
Social protection coverage (% population)	20·47% (19·6)	27·6% (24·31)	31·54% (27)	54·08%

Data are presented as mean (SD), percentage, or absolute number. Mortality (all ages) is expressed per 1000 population, and mortality for children younger than 5 years is expressed per 1000 livebirths. Economic growth is based on GDP in constant US dollars. ASMR=age-standardised mortality rate. GDP=gross domestic product. ODA=official development assistance. PPP=purchasing power parity.

We estimated the associations between different levels of ODA funding per capita and decreases in mortality rates by age groups (table 2). Higher levels of funding were associated with lower mortality. Specifically, in comparison with the baseline ODA category, the high ODA category was associated with a 24% reduction in overall ASMR (RR 0·76; 95% CI 0·67–0·87), 28% reduction in infant mortality rate (aged <1 year; 0·72; 0·57–0·92), 43% reduction in mortality rate for preschool children (aged 2–4 years; 0·57; 0·39–0·83), 33% reduction in mortality rate for children younger than 5 years (0·67; 0·50–0·89), and 27% reduction in mortality rate for school-aged children (aged 5–9 years; 0·73; 0·58–0·92). The findings withstood all the sensitivity and triangulation analyses mentioned above, showing that the results are robust (appendix p 16). Moreover, all sensitivity, triangulation, and complementary results satisfied the Bradford Hill criteria for epidemiological studies (appendix p 13). When causes of death in all-age mortality were analysed, high levels of ODA funding were associated with large mortality declines in major communicable diseases: tuberculosis, HIV/AIDS, lower respiratory infections, and malaria, among others (appendix p 28). When stratified by governance levels, we observed a stronger association in countries with better governance, with a 23% reduction in overall mortality (RR 0·77; 95% CI 0·71–0·83), compared with an 18% reduction (0·82; 0·75–0·88) in countries with poorer governance. For children younger than 5 years, the corresponding reductions in mortality were 45% (RR 0·55; 95% CI 0·48–0·63) in countries with better governance and 16% (0·84; 0·72–0·98) in countries with poorer governance (appendix p 46).

**Table 2:** Adjusted rate ratios from multivariable fixed-effects Poisson models with robust standard errors for the association between mortality rates and annual ODA per capita in the selected countries (n=59) from 2002 to 2021

	**Overall (ASMR)**	**Childhood mortality rates**			
		Infancy (0–1 year)	Preschool (2–4 years)	Child (<5 years)	School age (5–9 years)
**ODA quintiles**					
Baseline (mean 6·38; range 0–12·34)	1	1	1	1	1
Low (mean 18·68; range 12·35–24·40)	0·92 (0·89–0·96; p<0·0001)	0·93 (0·87–0·99; p=0·031)	0·91 (0·80–1·03; p=0·14)	0·92 (0·85–1·00; p=0·045)	0·92 (0·87–0·97; p=0·004)
Lower-intermediate (mean 30·21; range 24·41–35·97)	0·85 (0·78–0·93; p=0·001)	0·80 (0·71–0·91; p=0·0005)	0·72 (0·57–0·91; p=0·006)	0·77 (0·66–0·90; p=0·001)	0·80 (0·70–0·92; p=0·001)
Upper-intermediate (mean 42·81; range 35·98–52·12)	0·79 (0·72–0·88; p<0·0001)	0·76 (0·66–0·88; p=0·0002)	0·65 (0·51–0·83; p=0·0005)	0·72 (0·61–0·86; p=0·0002)	0·75 (0·65–0·88; p=0·0003)
High (mean 83·03; range 52·13 or more)	0·76 (0·67–0·87; p<0·0001)	0·72 (0·57–0·92; p=0·010)	0·57 (0·39–0·83; p=0·03)	0·67 (0·50–0·89; p=0·006)	0·73 (0·58–0·92; p=0·008)
**Control variables**					
Economic downturn	1·00 (0·96–1·04; p=0·90)	0·98 (0·91–1·05; p=0·56)	0·95 (0·84–1·08; p=0·48)	0·97 (0·89–1·06; p=0·53)	0·94 (0·85–1·04; p=0·24)
GDP per capita	0·95 (0·91–0·98; p=0·003)	0·95 (0·90–1·01; p=0·08)	0·87 (0·77–0·99; p=0·031)	0·93 (0·88–1·00; p=0·053)	0·82 (0·78–0·87; p<0·0001)
Conflict or wars index	1·20 (1·05–1·36; p=0·006)	1·35 (1·15–1·59; p=0·0002)	1·51 (1·17–1·97; p=0·002)	1·41 (1·16–1·71; p=0·0004)	1·53 (1·24–1·89; p=0·0001)
Fertility rate	1·03 (0·99–1·07; p=0·20)	0·99 (0·89–1·09; p=0·79)	1·03 (0·82–1·28; p=0·81)	0·99 (0·87–1·13; p= 0·86)	0·93 (0·81–1·06; p=0·26)
Urban population	0·94 (0·88–0·97; p=0·039)	0·89 (0·78–1·02; p=0·09)	0·91 (0·74–1·11; p=0·35)	0·87 (0·76–1·00; p=0·06)	0·98 (0·83–1·17; p=0·86)
Primary education	0·95 (0·92–0·98; p=0·004)	0·84 (0·76–0·93; p=0·001)	0·82 (0·71–0·94; p=0·006)	0·84 (0·76–0·93; p=0·001)	0·88 (0·81–0·96; p=0·005)
Basic drinking water	0·97 (0·93–1·02; p=0·23)	0·94 (0·87–1·01; p=0·14)	0·89 (0·78–1·00; p=0·06)	0·91 (0·84–0·99; p=0·032)	0·98 (0·88–1·09; p=0·67)
Health expenditure	0·94 (0·91–0·98; p=0·002)	0·84 (0·80–0·90; p=0·001)	0·72 (0·61–0·85; p=0·0001)	0·82 (0·76–0·88; p<0·0001)	0·74 (0·64–0·86; p<0·0001)
Social protection coverage	0·95 (0·89–1·01; p=0·10)	0·96 (0·88–1·04; p=0·32)	0·90 (0·78–1·05; p=0·19)	0·94 (0·85–1·04; p=0·25)	0·98 (0·88–1·10; p=0·76)
Number of observations	1177	1177	1177	1177	1177
Number of countries	59	59	59	59	59

Data are rate ratio coefficients (95% CI), unless otherwise stated. ASMR=age-standardised mortality rate. ODA=official development assistance. GDP=gross domestic product.

Regarding the potential mitigation scenarios, table 3 presents a summary of all the considered wealth, income, and socially efficient taxes and measures, including their rationale, desirability, and associated challenges. These interventions are well researched wealth redistribution tools that have been brought back to the fore due to the recent increase in economic inequalities, leading to concrete steps taken by a range of institutions to increase the feasibility of these interventions. Although these measures differ substantially in their nature and underlying mechanisms, they all share the capacity to mobilise substantial resources. The interventions take into consideration taxable economic players whose fortune is so high that the resulting reduction in their own welfare can be deemed as modest (ultra-high-net-worth individual's tax, global minimum tax for large corporations, and crypto tax, which can raise $32·6–343·5 billion, depending on the specific intervention);^22^, ^36^, ^37^, ^38^, ^39^ target a fundamental market failure underlying the current climate change crisis, which disproportionately affects the poorest regions (carbon tax; $102·2 billion);^37^ tackle an extremisation of financial markets (where speculation and volatility increase systemic risk), which has generated substantial gains for a few (Tobin tax; $234 billion);^40^ or echo decades-long demands for reform of the debtor–creditor system (tax on interest earnings from LMICs’ debt; $74–120 billion), which continues to entrench many poor countries in a vicious cycle of poverty.^41^

**Table 3:** Summary of wealth redistribution scenarios, including sources, amounts (US$ billions), rationale, strengths, and limitations

	**Rationale**	**Strengths**	**Limitations**
**Tax on ultra-high-net-worth individuals**			
(1) $90 billion (1% tax on billionaires);^22^ (2) $217·5 billion (2% tax on billionaires);^22^ and (3) $343·5 billion (3% tax on billionaires)^22^	A simple, presumptive tax scheme that sets a minimum tax rate for billionaires as a percentage of their wealth (should they already pay that percentage in taxes, no extra amount would be charged; should they pay less, only the difference would be charged)	Addresses tax avoidance of the extremely rich and reinstates some form of progressivity, albeit moderate (at 2% taxation, billionaires would pay an overall tax rate similar to the ordinary citizen)	Wealth evaluation might be difficult for some assets; risk of wealth concealment; need for global cooperation
**Global minimum tax for large corporations (revenues >€750 million): eg, corporate tax**			
$173·5 billion^36^	Aims to reduce the amount of global profit of large multinational enterprises, which is subject to low taxation (below a 15% effective tax rate)	Strongly demanded, as 36·1% of global profit is currently taxed below a 15% effective tax rate due to extensive use of tax incentives worldwide; addressing low-taxed profits would yield a more level playing field across multinational enterprises as well as governments, promoting healthy market competition rather than rentier capitalism	Wide implementation across jurisdictions might be difficult and corporations could attempt to shift profits to tax havens; yet, 45 countries are taking steps towards implementing this tax
**Carbon tax**			
$102·2 billion^37^	Tax imposed on polluters to partially compensate for the negative externality associated with the production of greenhouse gas emissions	Raises funds to mitigate pollution and disincentivises the excessive use of natural resources; positive reinforcing is effective in the fight against poverty, especially considering the disproportionately negative effects of climate change in many LMICs	Opposition from powerful lobbies; potentially regressive effects if not duly implemented; revenues are likely to decrease in the long run
**Crypto tax**			
(1) $32·6 billion (3% tax on top 10 000 holders; own calculations based on Makarov and Schoar's 2021 and 2025 estimates^38^); and (2) $100 billion^39^	Fiscal levy imposed on economic activities related to cryptocurrencies (eg, buying, selling, trading, mining, staking, or receiving); it might be based on capital gains, volumes, or transaction values; applicable to a set of increasingly popular digital assets not considered here (eg, non-fungible tokens)	Taxing cryptocurrencies is now recognised as a priority for governments across the world given the substantial amount of untaxed economic activity related to them, leading to loss of revenues, biased investment incentives, and potential illegal activities	Difficulties in setting precise tax regimes, because cryptocurrencies can be seen and used as investments or means of payment, or bothDifficulties regarding compliance; key hurdles are pseudonymity (identities appearing as nicknames on the blockchain ledger) and disintermediation (operations carried out without the need for intermediaries); yet, major progress is under way as 60 countries have committed to the Crypto Asset Reporting Framework
**Tobin tax**			
$234 billion^40^	Originally envisaged as a tax on short-term foreign exchange trades and then often intended more broadly as a tax on financial transactions; typically consists of a low-rate tax, but able to lead to substantial revenues given the high volume of transactions	Instrument offering a double divided: first, a tool to calm speculation, reduce instability due to market volatility, and strengthen countries’ monetary policy autonomy; and second, as a means to generate revenues that could finance global public goods, world welfare, and hold off the decline of development aid	Objections due to possible financial implications of the measure such as reduction of liquidity, decrease in market trading volumes and increased transaction costs; risk of substitution and relocation of operations to more tax-friendly jurisdictions if not widely applied
**Tax on interest earnings from LMICs’ debt**			
(1) $74 billion (100% of interest payments by LMICs to official creditors);^41^ and (2) $120 billion (50% of interest payments by LMICs to official creditors plus 25% of interest payments by LMICs to private lenders)^41^	Decisions taken by national governments, international organisations, and other creditors to reduce to various degrees or restructure, or both, LMICs’ external debt obligations; the aim is to reach debt sustainability and free up resources for health, education, and infrastructure to foster economic growth and poverty reduction	Particularly appealing considering that external debt servicing costs—the combination of debt repayments and payments of interest on existing debt—have doubled over the past decade for LMICs, due to a combination of high and rising debt stocks, rising interest rates and depreciating currencies; the poorest countries have been hit the hardest	Debt relief could: undermine confidence in a country's creditworthiness, making future borrowing more costly; increase the risk of moral hazard attitudes, whereby countries would contract unsustainable debts in the future in the hope of further relief; undermine the financial stability of multilateral institutions and their ability to offer affordable financing to poor countries

LMICs=low-income and middle-income countries.

When these sources of funding were included in the models and the resulting wealth redistribution scenarios were compared with the current defunding scenario, the models estimated several averted deaths associated with these redistributive policies, the magnitude of which depended on the funding source and its associated funding levels.

For all 59 countries combined, the microsimulation models projected that the difference in the cumulative number of deaths from 2026 to 2030 between the business-as-usual scenario and the current defunding scenario was 7 558 423 (95% uncertainty interval [UI] 5 509 244–9 653 447) for the overall population and 1 436 684 (842 306–1 981 779) for children younger than 5 years (table 4).

**Table 4:** Averted deaths under the various wealth redistribution scenarios, compared to the current defunding scenario and business-as-usual scenarios in the 59 selected countries, 2026–30

	**2026**	**2027**	**2028**	**2029**	**2030**	**Total**
**Business as usual**						
Overall	1 042 046 (664 959–1 455 190)	1 253 537 (894 906–1 619 262)	1 600 934 (1 191 871–2 029 867)	1 723 857 (1 274 531–2 181 897)	1 938 048 (1 451 857–2 433 054)	7 558 423 (5 509 244–9 653 447)
Children (aged <5 years)	159 176 (23 812–286 565)	202 146 (83 749–309 177)	322 914 (202 025–436 840)	357 255 (228 250–481 556)	395 192 (263 080–517 705)	1 436 684 (842 306–1 981 779)
**Crypto tax (3% tax on top 10 000 holders): $32·6 billion**						
Overall	1 042 047 (664 959–1 455 190)	1 253 537 (894 906–1 619 262)	1 600 936 (1 191 871–2 029 867)	1 297 428 (840 516–1 712 996)	1 398 035 (1 015 715–1 736 767)	6 591 984 (4 884 075–8 374 138)
Children (aged <5 years)	159 187 (23 812–286 565)	202 151 (83 749–309 177)	322 912 (202 025–436 840)	301 834 (187 261–414 539)	326 828 (230 757–430 565)	1 312 912 (802 926–1 785 121)
**Interest earnings debt tax (100% of interest payments by LMICs to official creditors): $74 billion**						
Overall	1 653 567 (962 400–2 410 983)	2 561 070 (1 586 791–3 479 590)	2 882 116 (1 901 028–3 810 980)	2 979 195 (1 979 522–3 924 261)	2 746 808 (1 823 832–3 623 934)	12 822 756 (8 424 196–17 095 511)
Children (aged <5 years)	176 523 (0–399 370)	303 553 (907–571 147)	420 363 (139 964–682 025)	450 941 (182 587–712 475)	433 522 (177 442–675 204)	1 784 903 (470 332–2 998 117)
**1% tax on ultra-high-net-worth individuals (billionaires): $90 billion**						
Overall	1 895 236 (1 075 208–2 735 377)	3 023 254 (1 916 525–4 050 160)	3 381 152 (2 257 041–4 455 596)	3 431 655 (2 300 166–4 553 475)	3 356 878 (2 258 461–4 401 612)	15 088 176 (10 032 879–19 858 520)
Children (aged <5 years)	209 961 (0–450 539)	371 655 (54 174–652 650)	490 181 (196 108–773 274)	515 832 (230 219–795 378)	522 236 (244 556–796 265)	2 109 865 (707 781–3 397 793)
**Crypto tax IMF2023: $100 billion**						
Overall	2 359 072 (1 585 319–3 140 575)	3 241 333 (2 185 337–4 296 096)	3 549 006 (2 481 357–4 572 986)	3 632 977 (2 550 597–4 663 611)	3 648 306 (2 555 306–4 709 019)	16 430 694 (11 320 584–21 299 209)
Children (aged <5 years)	271 748 (3882–510 866)	376 448 (65 935–669 873)	490 307 (187 390–773 383)	518 053 (223 060–793 797)	549 500 (263 633–818 686)	2 206 056 (784 579–3 553 582)
**Carbon tax: $102·2 billion**						
Overall	2 383 271 (1 531 065–3 216 579)	3 241 333 (2 185 337–4 296 096)	3 549 006 (2 481 357–4 572 986)	3 632 977 (2 550 597–4 663 611)	3 808 946 (2 754 575–4 853 864)	16 615 532 (11 538 934–21 485 559)
Children (aged <5 years)	277 759 (1330–526 176)	376 445 (65 935–669 873)	490 309 (187 390–773 383)	518 055 (223 060–793 797)	549 620 (260 740–816 818)	2 212 188 (774 614–3 580 811)
**Interest earnings debt tax (50% of interest payments by LMICs to official creditors plus 25% of interest payments by LMICs to private lenders): $120 billion**						
Overall	2 376 453 (1 434 352–3 253 755)	3 447 368 (2 386 741–4 522 162)	3 855 894 (2 695 186–4 965 642)	3 929 807 (2 781 101–5 054 666)	4 099 796 (2 964 885–5 254 774)	17 709 318 (12 351 645–22 871 053)
Children (aged <5 years)	264 682 (0–534 920)	400 946 (79 910–695 257)	523 352 (197 425–823 309)	549 741 (231 957–844 197)	580 004 (273 519–860 478)	2 318 724 (778 272–3 758 437)
**Corporate tax: $173·5 billion**						
Overall	3 070 366 (2 046 148–4 104 238)	3 791 938 (2 205 562–5 300 307)	4 324 409 (2 618 046–5 960 848)	4 391 609 (2 665 995–6 030 134)	4 553 677 (2 883 704–6 171 375)	20 131 999 (12 457 715–27 510 726)
Children (aged <5 years)	340 645 (18 088–623 504)	390 806 (0–774 801)	510 395 (88 764–901 093)	532 888 (122 779–914 064)	564 022 (168 287–934 932)	2 338 756 (357 598–4 210 899)
**2% tax on ultra-high-net-worth individuals (billionaires): US$217·5 billion**						
Overall	3 280 811 (2 230 039–4 336 384)	3 785 668 (2 116 134–5 348 441)	4 415 682 (2 662 226–6 067 045)	5 670 565 (3 752 451–7 521 246)	5 810 847 (3 967 549–7 651 187)	22 963 571 (14 944 443–30 871 693)
Children (aged <5 years)	366 198 (40 575–658 489)	383 414 (0–782 614)	511 526 (81 437–911 439)	704 858 (279 201–1 112 456)	729 483 (317 327–1 126 287)	2 695 480 (673 378–4 610 691)
**Tobin tax: $234 billion**						
Overall	3 390 638 (2 326 500–4 478 290)	4 031 227 (2 292 641–5 690 807)	5 622 922 (3 691 714–7 505 721)	5 670 565 (3 752 451–7 521 246)	5 810 847 (3 967 549–7 651 187)	24 526 199 (16 268 726–32 604 183)
Children (aged <5 years)	384 087 (56 041–679 372)	397 192 (0–797 479)	684 318 (236 327–1 107 193)	704 857 (279 201–1 112 456)	729 485 (317 327–1 126 287)	2 899 938 (850 725–4 836 305)
**3% tax on ultra-high-net-worth individuals (billionaires): $343·5 billion**						
Overall	3 599 129 (2 064 005–5 066 130)	5 388 816 (3 432 058–7 330 546)	6 074 005 (3 948 193–8 161 761)	7 166 392 (5 113 910–9 418 762)	7 281 592 (5 239 630–9 504 623)	29 509 934 (20 273 842–38 603 961)
Children (aged <5 years)	354 054 (0–732 839)	577 944 (114 014–1 001 343)	774 748 (308 412–1 217 102)	869 723 (400 382–1 314 410)	888 137 (439 005–1 320 408)	3 464 607 (1 235 930–5 609 331)

Data are averted deaths (95% uncertainty intervals) compared with the current defunding scenario. The total number of averted deaths for the business-as-usual scenario is 7 558 423 (5 509 244–9 653 447) for the overall population and 1 436 684 (842 306–1 981 779) for children younger than 5 years. LMICs=low-income and middle-income countries.

Among the wide range of policies and tax intensities evaluated, the largest number of averted deaths was associated with a 3% tax on ultra-high-net-worth individuals (billionaires). This policy could raise $343·5 billion per year and was associated with an estimated 29 509 934 averted all-age deaths by 2030 (95% UI 20 273 842–38 603 961), including 3 464 607 (1 235 930–5 609 331) deaths in children younger than 5 years. The smallest numbers of averted deaths were observed for a 3% crypto tax applied to the top 10 000 holders, which would raise $32·6 billion and was associated with 6 591 984 (95% UI 4 884 075–8 374 138) averted all-age deaths by 2030, including 1 312 912 (802 926–1 785 121) deaths in children younger than 5 years (table 4).

For lower tax rates on ultra-high-net-worth individuals, the associated averted all-age deaths were 15 088 176 (95% UI 10 032 879–19 858 520) for the 1% tax rate and 22 963 571 (14 944 443–30 871 693) for the 2% tax rate; for children younger than 5 years, the total averted deaths with the 1% and 2% tax rates were 2 109 865 (95% UI 707 781–3 397 793) and 2 695 480 (673 378–4 610 691), respectively. Corporate tax was associated with 20 131 999 (95% UI 12 457 715–27 510 726) averted deaths overall and 2 338 756 (357 598–4 210 899) averted deaths in children younger than 5 years. Depending on the scheme, tax on interest earnings from LMICs’ debt was associated with between 12 822 756 (95% UI 8 424 196–17 095 511) and 17 709 318 (12 351 645–22 871 053) averted overall deaths, and between 1 784 903 (470 332–2 998 117) and 2 318 724 (778 272–3 758 437) averted deaths in children younger than 5 years. Additional country-specific algebraic simulations further supported the robustness of these results (appendix p 61).

## Discussion

Our study provides the first evidence that implementation of the wealth redistribution policies proposed in recent high-level international agreements to support development—including the Rio de Janeiro G20 Leaders’ Declaration, the Sevilla Commitment, and the OECD/G20 Inclusive Framework on BEPS, among others—could offset the health impacts of the current ODA defunding crisis and be associated with 6·6–29·5 million averted deaths by 2030, depending on policy type, target population, and taxation level.

Our models indicate that ODA could have substantially contributed to the reduced mortality rates in low-income and lower-middle-income countries over the past two decades. Yet, the recent ODA funding cuts could impair these gains and reverse the progress achieved over the past 20 years.

Mitigation strategies based on wealth redistribution policies—whereby ODA is supplemented by revenues from taxing the world's biggest concentrations of wealth—could have a major impact. For example, ensuring that more than 3000 billionaires pay a tax equivalent to 3% of their wealth was associated with 29·5 million averted deaths among the most vulnerable populations by 2030 (while a 1% tax would correspond to 15·1 million averted deaths). Other taxation mechanisms, such as a Tobin tax and a minimum global tax for transnational corporations with revenues above €750 million, were associated with an estimated 24·5 million and 20·1 million averted deaths, respectively.

Wealth redistribution policies have gained considerable momentum in political debates, particularly among high-income ODA-donor countries. In the 2026 World Economic Forum in Davos, nearly 400 millionaires and billionaires from 24 countries called on global leaders to increase taxes on the super-rich.^42^

Wealth taxes have been implemented in a few countries. In 2022, the Spanish central government introduced a solidarity wealth tax, with rates ranging from 1·7% to 3·5% on individuals holding net assets above €3 million.^43^ Other countries, such as Sweden, implemented a wealth tax for several decades before abolishing it in 2007.^44^ In France, a recent draft law proposing a 2% tax on ultra-high-net-worth individuals was rejected by the Senate.^45^

Evidence of the effects of wealth redistribution policies on population mortality remains scarce. To date, only one study has evaluated these policies, showing that—when applied to the US context—they could substantially reduce survival inequities and increase population longevity.^46^

From a neoliberal economic perspective, one could argue that leaving resources in the hands of wealthy individuals or corporations might generate societal benefits—consistent with theories of trickle-down economics^47^—and that such potential benefits were not incorporated into our models. However, such theories are facing increasing conceptual critiques and are only weakly supported by recent empirical findings.^48^, ^49^, ^50^ It is highly implausible that continued wealth concentration at current or higher levels could produce health gains, whether in the short or long term, that are able to outweigh the millions of lives saved estimated in our study—and more so if we consider that health outcomes follow clear socioeconomic gradients.^51^

Wealth redistribution policies from the Global North to the Global South could also be framed as a form of restorative justice, understood as a potential response to historically rooted injustices, including colonial extraction, slavery, and enduring exploitative mechanisms and structural inequalities in the contemporary global economy.^52^, ^53^

Our retrospective and forecasting results show that accounting for governance is important when estimating the health impact of ODA on mortality, because governance fundamentally shapes whether funds are converted into effective, sustained services rather than being lost to inefficiency, instability, uncertainty, poor institutional enforcement, or corruption, among other causes. Strong governance improves planning, procurement, workforce management, monitoring, and cross-sector coordination, all of which increase the fidelity and reach of health interventions and amplify mortality reductions.^54^, ^55^, ^56^ By contrast, weak governance attenuates the expected benefits, although the effects of ODA remain strong and consistent even in these countries (appendix p 46).

The magnitude of the impact of ODA on mortality observed in our findings is consistent with previous estimates derived from diverse methodological approaches. Regarding retrospective impact evaluations, older studies examining USAID funding per capita reported an associated reduction in child mortality of 29 deaths per 1000 livebirths between 2000 and 2016.^26^ More recent research across 133 LMICs showed that high levels of USAID funding per capita were associated with a 15% reduction in overall mortality and a 32% reduction in child mortality between 2001 and 2021.^6^ Similarly, an evaluation of 93 LMICs linked high levels of ODA funding per capita to a 23% reduction in overall mortality and a 39% reduction in child mortality between 2002 and 2021.^11^ Regarding forecasting analyses of ODA defunding, the aforementioned study of 133 LMICs estimated that a near-complete dismantling of USAID programmes could result in 14 million deaths by 2030, including 4·5 million children younger than 5 years.^6^ The other forecasting analysis of 93 LMICs—applying an 11% annual reduction scenario similar to our own—estimated 9·4 million overall deaths and 2·5 million deaths in children younger than 5 years by 2030.^11^ Additional analyses suggest that USAID programmes averted approximately 3·3 million all-cause, all-age deaths annually.^25^ Furthermore, research focusing on a subset of LMICs estimated that eliminating US foreign health assistance between 2025 and 2030 could lead to more than 4 million additional deaths from AIDS, more than 600 000 tuberculosis deaths, and 2·5 million child deaths, alongside millions of unintended pregnancies and unsafe abortions.^12^ Finally, a report has estimated that a 30% reduction in development assistance for health could cause more than 16 million deaths in children younger than 5 years by 2045.^13^ Although the above estimates are derived from different datasets, country samples, study periods, and statistical models, they are nonetheless consistent with and comparable, in terms of effect sizes, to our estimated reductions of 24% and 33% in overall and under-5 mortality, respectively, associated with high ODA funding, as well as the projected impact of current ODA defunding scenarios up to 2030, amounting to 7·6 million deaths, including 1·4 million deaths among children younger than 5 years.

Over the past decades, ODA has supported a wide spectrum of interventions across multiple sectors,^7^ ranging from governance and human rights to public infrastructure such as roads, water and sanitation systems, and electricity networks. ODA has also financed programmes in agriculture and food security, environmental protection, education and vocational training, poverty and inequality reduction, and an extensive array of health interventions—including health promotion, vaccination campaigns, provision of essential medicines and diagnostics, and the strengthening of health systems. Additionally, ODA has played a central role in responding to humanitarian crises caused by conflicts and natural disasters, supporting emergency relief, reconstruction, and conflict resolution efforts. Interventions across these domains can profoundly influence health outcomes by shaping not only direct health-care delivery but also the broader structural and social determinants of health. Poverty reduction initiatives alone, for example, have shown substantial effects in lowering both adult and child mortality. Beyond addressing these upstream determinants, ODA plays a crucial role in sustaining health systems, supporting disease-control and eradication programmes, and strengthening preparedness for infectious disease outbreaks and epidemics.

Overall, there is considerable evidence demonstrating the effectiveness of ODA, as indicated by global studies, meta-analyses, and specific case studies.^57^, ^58^, ^59^, ^60^, ^61^ However, its impact appears to be more pronounced when the objectives of the assistance and the funded interventions are clearly defined, evidence based, and jointly agreed upon by donor and recipient countries.^58^, ^61^ Effectiveness can be further enhanced when programmes are locally owned, supported by strong institutions, and aligned with national priorities. Good governance, clear monitoring frameworks, and sufficient administrative capacity to implement projects properly also seem to play an important role.^58^, ^60^, ^61^ Moreover, interventions tend to be more successful when they are well targeted, sustainable over the long term, and not fragmented or overlapping across multiple uncoordinated donors or implementing agencies.^58^, ^61^, ^62^

The dismantling of USAID has triggered widespread alarm among humanitarian and development organisations, which have condemned both the abruptness and lack of adequate notice and the absence of a structured plan for a phased transition. Simultaneously, major announced aid cuts from the UK, France, Germany, the Netherlands, Belgium, and several other donors signal a systemic funding crisis across the humanitarian and development architecture.^8^, ^9^ This abrupt withdrawal leaves little opportunity for the design or implementation of adaptive strategies, making the most severe consequences largely unavoidable unless alternative public or private sources of funding or donors rapidly step in.

This study has limitations. First, the causal interpretation of the statistical associations presented here warrants careful consideration. Although the application of sensitivity and triangulation analyses, combined with the use of the Bradford Hill criteria, provides evidence for a potential causal interpretation of the findings, the study design cannot entirely preclude residual confounding or the influence of unmeasured factors. Consequently, these results should not be interpreted as evidence of causality. A second limitation concerns the reliance on aggregate-level (ecological) data. Analyses conducted at this level are inherently subject to the risk of ecological fallacy, meaning that associations observed at the country level might not necessarily hold true at the individual or household level. Nevertheless, the aggregate design also offers distinct analytical advantages. It enables the capture of broader, system-level dynamics and potential spillover effects that individual-level studies might fail to detect, which is particularly relevant in the context of national or regional development programmes where indirect benefits and network effects can play a crucial role. Moreover, although this study used a comprehensive set of covariate adjustments—derived from the established literature—and incorporated two-way fixed effects for both country and year, the possibility of residual confounding cannot be fully excluded. Sensitivity analyses based on alternative adjustment sets lend further support to the robustness of the findings and suggest that the estimated effects of ODA funding are unlikely to be substantially biased by omitted variables. Nevertheless, as in all observational analyses, unmeasured or context-specific factors might still contribute to the observed associations. Importantly, the sensitivity and triangulation analyses consistently revealed statistically significant relationships, and the magnitude of the estimated effects aligns with those reported in comparable studies, reinforcing both their internal validity and external plausibility. A further limitation concerns the study's inability to disentangle the specific interventions or causal pathways through which ODA funding per capita influences mortality outcomes. Although extensive sensitivity and triangulation analyses, as well as comparisons with previous empirical work, enhance confidence in the overall effect estimates, they cannot identify the relative contribution of individual ODA-funded initiatives. Only programme-specific evaluations could precisely assess the effectiveness of distinct interventions within the broader ODA portfolio. A further limitation pertains to the inherent uncertainty of the forecasted scenarios. This uncertainty arises primarily from two sources: first, the difficulty in predicting with precision which specific ODA programmes will be scaled back or terminated over the short, medium, and long term; and second, the challenge of incorporating potential exogenous shocks, such as geopolitical instability, economic downturns, or climate-related crises, that could substantially alter future trajectories. Although these factors introduce variability into the projections, the primary objective of this analysis was not to capture every possible permutation of future aid patterns. Crucially, the analysis was not designed to forecast precise all-age or child mortality rates by 2030, but rather to quantify the relative divergence in mortality outcomes between scenarios, isolating the effect of changes in the primary exposure variable: ODA funding. This comparative approach provides a robust framework for assessing the potential human costs associated with large-scale aid withdrawal, while acknowledging the uncertainty that necessarily accompanies forward-looking projections. Consequently, although exogenous factors such as macroeconomic fluctuations, climate-related shocks, or armed conflict might influence future mortality trends and cause deviations from our projections, the relative comparisons between scenarios—particularly the estimated mortality RRs and differences in the number of deaths—are expected to remain essentially stable and robust. These comparative measures capture the directional impact of changes in ODA funding under consistent assumptions, thereby providing a reliable basis for interpreting the potential consequences of large-scale aid reductions. Another important limitation concerns the simplified representation of the implementation mechanisms underlying wealth redistribution policies. Although it was not the objective of our study to detail how global fiscal redistribution mechanisms would operate in practice—specifically, how taxes would be collected across countries and reallocated to ODA—and although we incorporated a bounded exponential function to approximate initial implementation challenges as well as avoidance and evasion dynamics, the real-world implementation of such policies could face considerable delays and setbacks. These factors could, in turn, result in effects over the timeframe up to 2030 that are lower than those estimated by our models.

Despite the limitations inherent to observational and forecasting approaches, the principal strength of this study lies in the breadth and rigour of its analytical framework. A comprehensive series of sensitivity analyses consistently confirmed the robustness of the results across alternative model specifications, variable selections, and estimation strategies. Moreover, the estimations from the triangulation analyses, integrating difference-in-differences supplemented by propensity score matching, provided convergent evidence supporting the potential causal interpretation of the ex-post impact evaluation findings. The integration of these empirical results with externally validated microsimulation models and alternative forecasting analyses further enhanced the internal consistency and robustness of the projected scenarios (appendix p 40).

Our findings indicate that ODA funding is likely to have significantly reduced mortality across the most vulnerable countries over the past two decades and highlight the potentially severe consequences of the current abrupt and substantial reductions in this funding. In a world marked by extreme and widening inequalities and an unprecedented concentration of wealth, our results show the potential of alternative wealth redistribution policies—proposed in recent high-level international agreements to support global development—to mitigate the negative impacts of ODA defunding. Such policies could save millions of lives and substantially advance progress towards the health-related UN Sustainable Development Goals by 2030.

### Contributors

### Data sharing

The data used are public and available from the OECD CRS (https://data-explorer.oecd.org/) the Global Burden of Disease Study (https://vizhub.healthdata.org/gbd-results/), WHO (https://platform.who.int/mortality), and the World Bank (https://data.worldbank.org/region/world).

## Declaration of interests

We declare no competing interests.
