# Supplementary material for: Wealth redistribution policies to mitigate the impact of development assistance defunding: a retrospective evaluation and forecasting analysis up to 2030
Source: Lancet. 2026 Oct 3;408(10562):1346–60. doi: 10.1016/S0140-6736(26)00975-X (PMC13633129; doi:10.1016/S0140-6736(26)00975-X)
Supplement: Supplementary file 1 [file mmc1.pdf]

THE LANCET

Supplementary appendix

This appendix formed part of the original submission and has been peer reviewed. We post it as supplied by the authors.

Supplement to: Barreix Sibils G, Rezende Anderle RV, Ferreira da Silva A, et al. Wealth redistribution policies to mitigate the impact of development assistance defunding: a retrospective evaluation and forecasting analysis up to 2030. *Lancet* 2026; published online Sept 7. https://doi.org/10.1016/S0140-6736(26)00975-X.

Supplementary Materials

Wealth Redistribution Policies to Mitigate the Impact of Development Assistance Defunding: a Retrospective Evaluation and Forecasting Analysis up to 2030

Content

PART I – SUMMARY OF ODA, DATA SOURCES AND GENERAL METHODOLOGY 7
1. JUSTIFICATION OF SCOPE OF STUDY RELATED TO THE ODA 7
1.1. A comprehensive analysis of ODA investment areas beyond health 7
1.2. Choosing to work with the ODA disbursement per capita 7
1.3. The 59 Countries Selected 7
1.4. The Importance of Using Categorical Variables 8
2. DATASET 9
2.1. Data sources 9
2.2. Variables details and Interpolation and extrapolation method 10
2.3. Main variables graph across years 11
PART II - RETROSPECTIVE ANALYSIS 13
3. EMPIRICAL METHODS 13
3.1 Poisson Regression - Fixed effects 13
4. RESULTS 13
4.1. Sensitivity Analysis 16
4.2. Triangulation 40
PART III – FORECASTING ANALYSIS 43
5. PURPOSE OF THE FORECASTING AND ITS APPLICATIONS 43
6. DESCRIPTION OF THE FORECASTING METHODOLOGY 43
6.1. Scenarios 43
6.2. Governance 45
7. DATA SOURCE AND INPUTS 47
8. POISSON MULTIVARIABLE REGRESSION MODELS WITH ROBUST STANDARD ERRORS AND FIXED-EFFECTS 47
9. CALIBRATION, TIME TREND, AND MONTE CARLO SIMULATION 48
9.1. Calibration 48
9.2. Time trend 49
9.3. Monte Carlo Simulation 50
10.VALIDATION 51
11.GRAPH OF AVERTED DEATHS BY SCENARIO 52
12.SUB-GROUP ANALYSIS FOR BY GOVERNANCE STRATA 53
13.SUB-GROUP ANALYSIS FOR SUB-SAHARAN COUNTRIES 57
14. FORECASTING RESULTS WITHOUT GOVERNANCE ADJUSTEMENT 59
15. MAIN LIMITATIONS 61
16. FORECASTING TRIANGULATION 61

REFERENCES ... 63

List of Figures

WEB FIGURE 1 Map of ODA quintile (2002 and 2021) by country.......... 9
WEB FIGURE 2 Interpolated and Extrapolated control variables boxplot for selected countries (N=59), from the period 2002-21.......... 10
WEB FIGURE 3 Main variables average year line graph for selected countries (N=59), from the period 2002-21. 12
WEB FIGURE 4 Evidence of common support in mortality rate distributions for treatment and control groups defined by ODA per capita disbursement levels.......... 40
WEB FIGURE 5 Assessment of the parallel trends assumption in mortality rates between treatment and control groups defined by ODA per capita disbursement levels. 41
WEB FIGURE 6 Mean Governance Ratio by year (59 countries, from 2002 to 2021).......... 46
WEB FIGURE 7 RMSE results of all LHS parameters for the child mortality rate model governance level one.......... 48
WEB FIGURE 8 RMSE results of 100 LHS parameters for the child mortality rate model governance level two.......... 48
WEB FIGURE 9 Q-Q plot of the residuals from the linear regression model fitted to the ASMR per 1,000 inhabitants for the governance level one (left) and two (right).......... 50
WEB FIGURE 10 Q-Q plot of the residuals from the linear regression model fitted to child mortality rate per 1,000 live births for the governance level one (left) and two (right).......... 50
WEB FIGURE 11 Simulated Mortality Rates with Uncertainty Intervals and Projected Values based on WPP2024 (2022–2023).......... 51
WEB FIGURE 12 Simulated Child Mortality Rates with Uncertainty Intervals and Observed Values from UNICEF (2022–2023).......... 52
WEB FIGURE 13 Averted Deaths by scenario 53

List of Tables

WEB TABLE 1 Data variables details.......... 9
WEB TABLE 2 Analytical strategy used according to Bradford Hill`s criteria.......... 14
WEB TABLE 3 Hausman test between fixed effect and random effect Poisson models of main model.......... 17
WEB TABLE 4 Rate Ratios from the Poisson models for the association between Overall ASMR and ODA quintiles (crude, country Fixed Effects, country-year Fixed Effects and fully adjusted), 2002-21 18
WEB TABLE 5 Rate Ratios from the Fixed Effects Poisson models for the association between Overall ASMR and ODA by different ODA specifications (median, terciles, quartiles, quintiles, deciles), 2002-21 19
WEB TABLE 6 Rate Ratios from the Poisson models for the association between Overall ASMR and ODA value per capita (crude, Country-year Fixed Effects, fully adjusted), 2002-21.......... 21
WEB TABLE 7 Rate Ratios from the Poisson models for the association between Overall ASMR and ODA logarithmic of value per capita (crude, Country-year Fixed Effects, fully adjusted), 2002-21 21
WEB TABLE 8 Rate Ratios from the Fixed Effects Poisson models for the association between mortality and ODA quintiles, for Overall ASMR and under-5 children for 103 LMIC´s, 2002-21 22
WEB TABLE 9 Rate Ratios from the Fixed Effects Poisson models for the association between mortality and ODA quintiles using different time Fixed Effects (Main model-specific year dummies, 5-year period dummy, 4-year period dummy, yearly dummies), 2002-21.......... 23

WEB TABLE 10 Rate Ratios from the Fixed Effects Poisson models for the association between mortality and ODA quintiles by different age sub-groups (Neonatal - 0 to 28 days, Infancy - 0 to 1 year, Preschool - 2 to 4 years, Child – Under-5 years, School-a age - 5 to 9 years, Adult - 10 to 54 years, Elderly - 55+ years, Overall-ASMR), 2002-21 25
WEB TABLE 11 Rate Ratios from the Fixed Effects Poisson models for the association between Overall ASMR and ODA quintiles by sex (Male and Female), 2002-21 25
WEB TABLE 12 Rate Ratios from the Fixed Effects Poisson models for the association between mortality and ODA quintiles by age-subgroups for females (Children and adolescents - 0 to 14 years, Youth and young adults - 15 to 29 years, Young to middle-age adults - 30 to to 44 years, Middle-age adults - 45 to 59, Elderly - 60+ years, Overall ASMR), 2002-21 26
WEB TABLE 13 Rate Ratios from the Fixed Effects Poisson models for the association between mortality and ODA quintiles by regions (Sub-Saharan and non-Sub-Saharan countries), 2002-21 27
WEB TABLE 14 Rate Ratios from the Fixed Effects Poisson models for the association between Overall ASMR and ODA quintiles by mortality causes (Tuberculosis, HIV/AIDS, Lower Respiratory Infections, Nutritional Deficiencies, Diarrhoeal Diseases, Neglected Tropical Diseases, Malaria), 2002-21 28
WEB TABLE 15 Rate Ratios from the Fixed Effects Poisson models for the association between mortality and ODA quintiles (for Overall ASMR and children under-5) by different sectors, 2002-21 28
WEB TABLE 16 Rate Ratios from the Fixed Effects Poisson models for the association between mortality and ODA quintiles (for Overall ASMR and children under-5) for DAC country members donors, 2002-21 29
WEB TABLE 17 Negative controls 30
WEB TABLE 18 Overall (ASMR) Mortality model with different time trend specifications ... 31
WEB TABLE 19 Overall (ASMR) Mortality model continuous exposition and year adjusting variable 34
WEB TABLE 20 Overall (ASMR) Mortality model with year lag effects 35
WEB TABLE 21 Overall (ASMR) Mortality model with consolidation effects 36
WEB TABLE 22 Overall (ASMR) Mortality model with year duration effects 37
WEB TABLE 23 Overall (ASMR) Mortality model with no health expenditure variable and with alternative health variables 38
WEB TABLE 24 Overall (ASMR) Mortality model with alternative covariates sets 39
WEB TABLE 25 Propensity Score–Matched Difference-in-Differences Estimates of the impact of intermediate-to-high ODA per capita disbursements on overall and child mortality in low-income countries, 2002–2019. 42
WEB TABLE 26 Rate Ratios from the difference-in-difference fixed effect Poisson models for the association between mortality rates (Overall ASMR and child) with intermediate to high ODA disbursement, in 2002 and 2019, in Low Income countries/territories. 42
WEB TABLE 27 Covariate balance before and after propensity score matching in low-income countries (LIC). 43
WEB TABLE 28 Adjusted rate ratios from multivariable fixed-effects Poisson models with robust standard errors for the association between mortality rates and annual ODA per capita in both governance strata from 2002 to 2021. 46
WEB TABLE 29 Final estimates after model refinement and calibration based on minimum RMSE. 49
WEB TABLE 30 Comparison between Simulated and external sources in 2022 and 2023 across 59 Countries. 51
WEB TABLE 31 Overall main results by governance strata (averted deaths, by year and total), 2026-2030 53

WEB TABLE 32 Child main results by governance strata (averted deaths, by year and total), 2026-2030 55
WEB TABLE 33 Main results stratifications only for 32 Sub-Saharan Countries (averted deaths, by year and total), 2026-2030 57
WEB TABLE 34 Forecasting results comparison with non-adjusted by governance forecasted results 60
WEB TABLE 35 Comparative risk assessment and microsimulation averted deaths results by scenario, for Overall and child age groups. 61

PART I – SUMMARY OF ODA, DATA SOURCES AND GENERAL METHODOLOGY

1. JUSTIFICATION OF SCOPE OF STUDY RELATED TO THE ODA

1.1. A comprehensive analysis of ODA investment areas beyond health

The social determinants of health framework show that there is an upstream influence of the different social dimensions on health outcomes. These include dimensions such as education, access to healthcare, social protection, macroeconomic conditions, access to water and sanitation, among others.

An analysis of the impact of Official Development Assistance (ODA) on mortality only considering the direct ODA healthcare sectors would be an underestimation of what ODA in a broader sense can influence mortality evolution in a country. Because of that, we chose to consider a broader set of ODA sectors, considering that these would be sectors that in a relatively short term could impact mortality. In this sense we left out sectors that in the chain of effects may be too distant to mortality or that their effects may arise in a longer term, greater than the modelling techniques utilized can capture.

We included the following sectors:

- Social infrastructure and services. This includes Education, Health, Population policies/Programmes & reproductive health, Water supply & sanitation, Government and civil society, and Other social infrastructure and services.
- Multi-sector/Cross-cutting. This includes General environment protection, Other multisector such as urban and rural development food security and safety, disaster reduction among others.
- Humanitarian aid. This includes Emergency response, Reconstruction relief and rehabilitation, and Disaster prevention and preparedness.

Also, other sectors' subsets were included in the sensitivity analyses.

1.2. Choosing to work with the ODA disbursement per capita

We use ODA Disbursements per capita as it shows funding delivered to each country relative to population, allowing for meaningful comparisons and better assessment of aid impact.

For this, we took the OECD flow-based ODA disbursement data for the period 2002-2021 for Official Donors of Social infrastructure and services, Multi-sector/Cross-cutting and Humanitarian aid sectors, for all channels and modalities in US$ constant prices[1].

1.3. The 59 Countries Selected

From a total of 204 countries and territories worldwide, we selected 59 low and lower-middle-income countries (LICs and LMCs), excluding Small Island Developing State (SIDS`s), the Syrian Arab Republic and South Sudan with consolidated data available through 2021.

The exclusion of the upper-middle and high-income countries (UMIC`s and HIC`s) is justified for two main reasons. First, we intended to analyse a restricted subgroup of poorer countries and because HIC`s are not the primary focus of ODA donations, which are directed toward low- and middle-income countries. Second, these exhibit very different socioeconomic and health dynamics compared to the countries in focus, which could introduce bias and undermine the comparability of the results.

Excluding SIDS is due to their specific characteristics in terms of geography, population, economy and development challenges, including their ODA receipt dynamics.

Excluding the Syrian Arab Republic is due to the extreme flow of ODA this country received due to war and conflict in comparison to the rest of the sample. Excluding South Sudan is due to the fact this country is considered in the statistics as a country only for about half of the period, before being considered as part of Sudan.

Therefore, this exclusion is crucial for ensuring the internal validity of the study, maintaining a more consistent and relevant scope for the countries under analysis.

Algeria, Burundi, Central African Republic, Eritrea, Venezuela and Yemen were not included due to covariates data missingness.

1.4. The Importance of Using Categorical Variables

We used categorized variables in this study because they offer clear implementation thresholds for policy decisions, reduce the impact of outliers, and allow straightforward dose-response evaluation—especially when the relationship is non-linear and the exposure variable has multiple categories. Specifically, categorizing ODA per capita helps clarify how different funding levels relate to changes in age-standardized mortality rates, a common approach in epidemiology to assess causal links.[2–11]

Additionally, we confirmed results with both continuous and alternative categorizations of the exposure, considering:

1) The unknown functional form of the dose-response relationship.

2) Potential outlier influences continuous models.

3) Categories reflecting meaningful intensity levels of ODA funding, making Rate Ratios easier to interpret, as supported by prior research.[5,8,10,11]

The WEB FIGURE 1 below presents world maps that visually summarize each country's ODA quintile in 2002 and in 2021.

WEB FIGURE 1 Map of ODA quintile (2002 and 2021) by country.

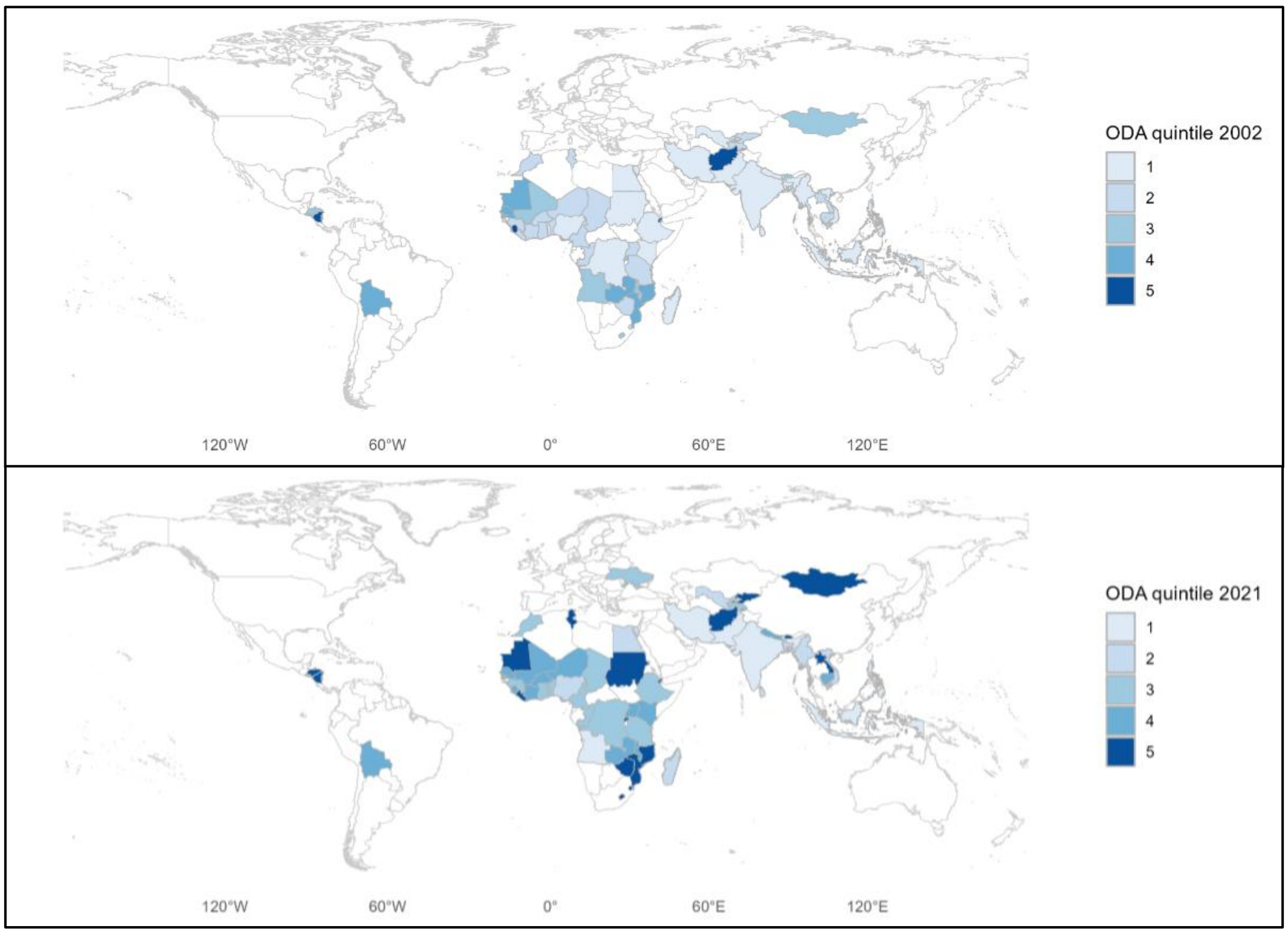

Source: Authors' elaboration based on 2002-2021 ODA data.

2. DATASET

2.1. Data sources

The study's data was sourced from organizations listed in WEB TABLE 1 and aggregated at the country or territory level. For variables with missing values by year or country, we used interpolation for imputation as described in Section 2.2.2 of this supplement.

WEB TABLE 1 Data variables details

Variable	Unit of Analysis	Source	Link	Source variable from the data source
Mortality	Country/Territory level	GBD	LINK 1	
ODA disbursement	Country/Territory level	OECD	LINK 2	
Population estimates	Country/Territory level	GBD	LINK 1	
Economic growth (%)	Country/Territory level	WBDI	LINK 3	GDP (constant 2015 US$)
GDP per capita PPP (Constant US$)	Country/Territory level	WBDI	LINK 3	GDP per capita, PPP (constant 2021 international $)
Conflict/wars index	Country/Territory level	V-Dem	LINK 4	v2casoe_4 (A national state of emergency was in place due to an armed conflict/war, domestically or internationally)

Fertility rate	Country/Territory level	WBDI	LINK 3	Fertility rate, total (births per woman)
Urban population (% population)	Country/Territory level	WBDI	LINK 3	Urban population (% of total population)
Primary education (% population)	Country/Territory level	WBDI	LINK 3	Educational attainment, at least completed primary, population 25+ years, total (%) (cumulative)
Basic drinking water (% population)	Country/Territory level	WBDI	LINK 3	People using at least basic drinking water services (% of population)
Health expenditure per capita PPP	Country/Territory level	WBDI	LINK 3	Current health expenditure per capita, PPP (current international $)
Social protection coverage (% population)	Country/Territory level	WBDI	LINK 3	Coverage of social insurance programs (% of population)
Governance indicator	Country/Territory level	WBGI	LINK 5	Mean of the six governance dimensions ratios (0-100)

Note: ODA= Official Development Assistance. GDP = Gross Domestic Product. PPP = Purchasing Power Parity. GDB = Global Burden of Disease. OECD = Organization for Economic Co-operation and Development. WBDI=World Bank Development Indicators. V-Dem = Varieties of Democracy Project. WBGI=World Bank Governance Indicators

2.1.1. Study Scope: Selected Periods for Retrospective and Forecasting Analyses

The total analysis period spans from 2002 to 2030, divided as follows:

- Retrospective Analysis: This covers the period from 2002 to 2021.
    - the starting year, 2002, was chosen as it marks the first year with available data on ODA disbursements by country.
    - the final year, 2021, was chosen because the GBD data was available until 2021 when the analyses were done.
- Forecast Analysis: This refers to the period from 2026 to 2030.

2.2. Variables details and Interpolation and extrapolation method

2.2.1 Variables details

Economic downturn was computed as a dummy variable that expresses that the GDP (constant 2015 US$) growth in the country/year is lower than zero. The conflict/wars index was directly utilized in the model as the index is generated by V-Dem (v2casoe_4 variable in V-Dem data). As the value of this is higher, we could interpret has a greater security of the existence of conflict-wars in the country/year.

2.2.2 Interpolation and extrapolation method

Some of control variables are not available in total period for the period 2002-21. These were primary education and social protection coverage, which had presence of missing data. Because of this, they were linearly interpolated and extrapolated by maintaining the values of the nearest year with available data. These techniques have been previously employed in panel data studies. [5,8,10,12,13]

No outcome (mortality) or exposure (ODA amount) variables were interpolated. However, using interpolated and extrapolated variables ultimately enhanced model control and precision.

WEB FIGURE 2 Interpolated and Extrapolated control variables boxplot for selected countries (N=59), from the period 2002-21.

Primary education (% population)

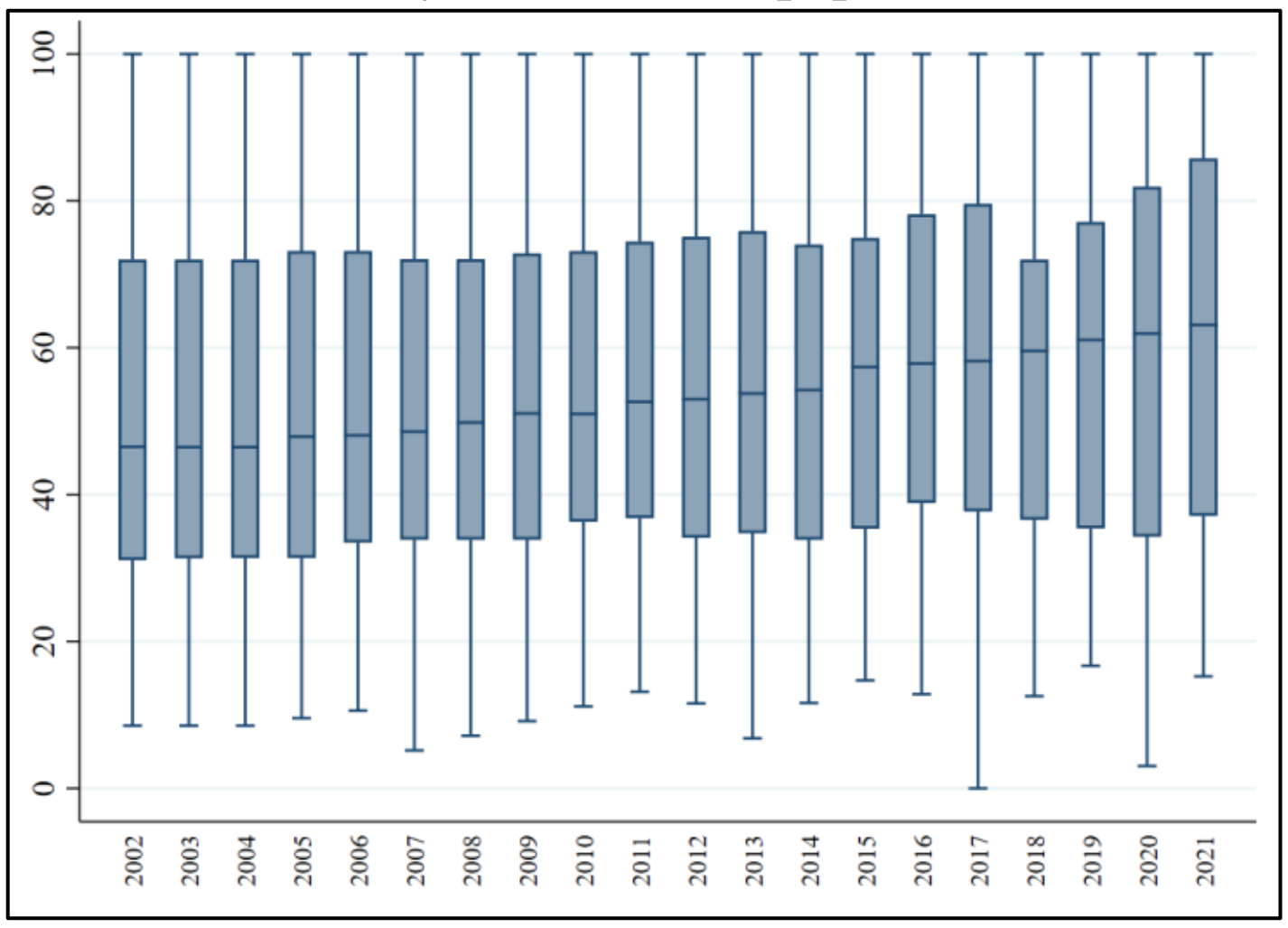

Social protection coverage (% population)

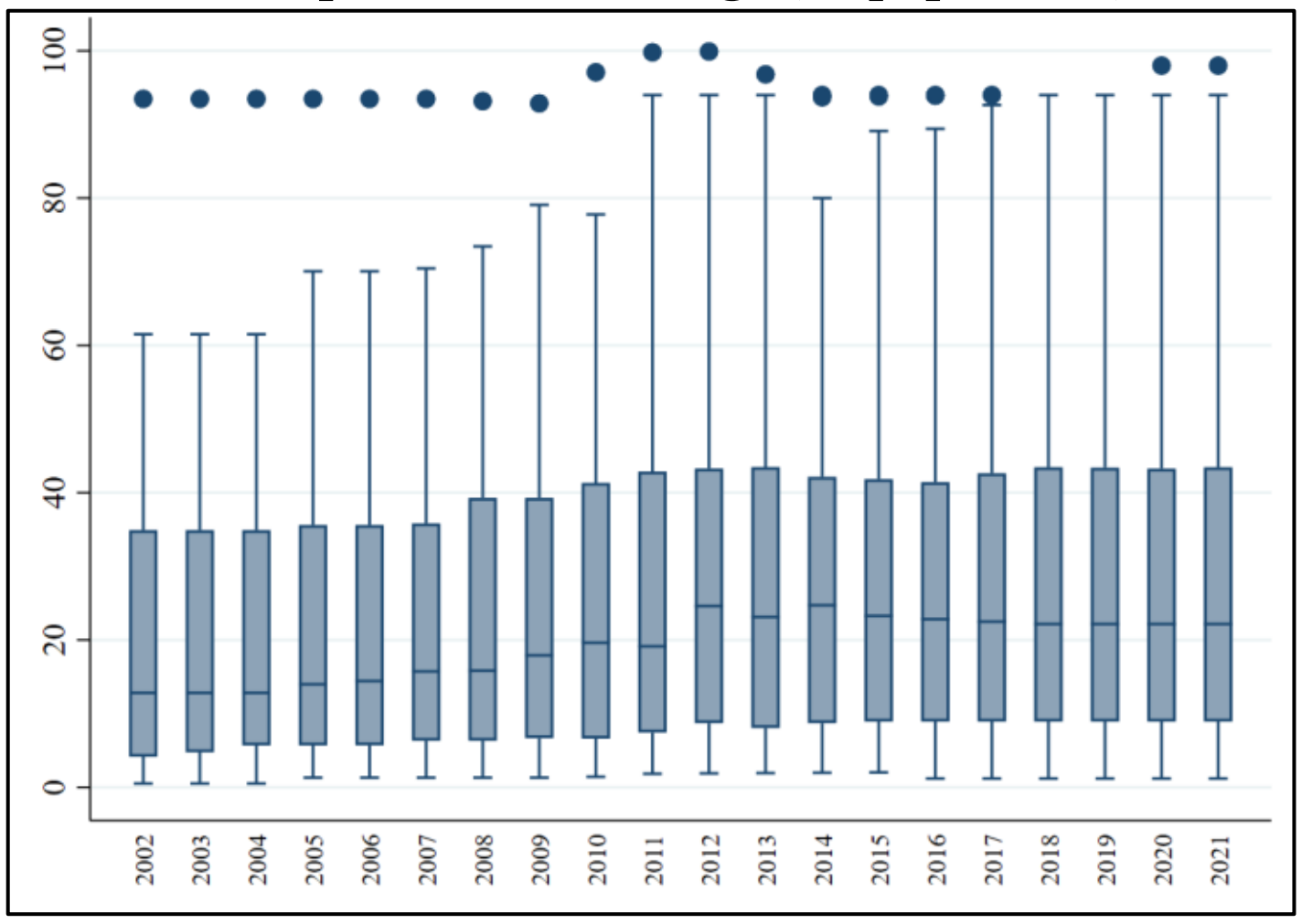

2.3. Main variables graph across years

Below, in Figure 3, we show a graph across the time (years 2002-2021) of the average year variable value (the sum for the country's population variable).

WEB FIGURE 3 Main variables average year line graph for selected countries (N=59), from the period 2002-21.

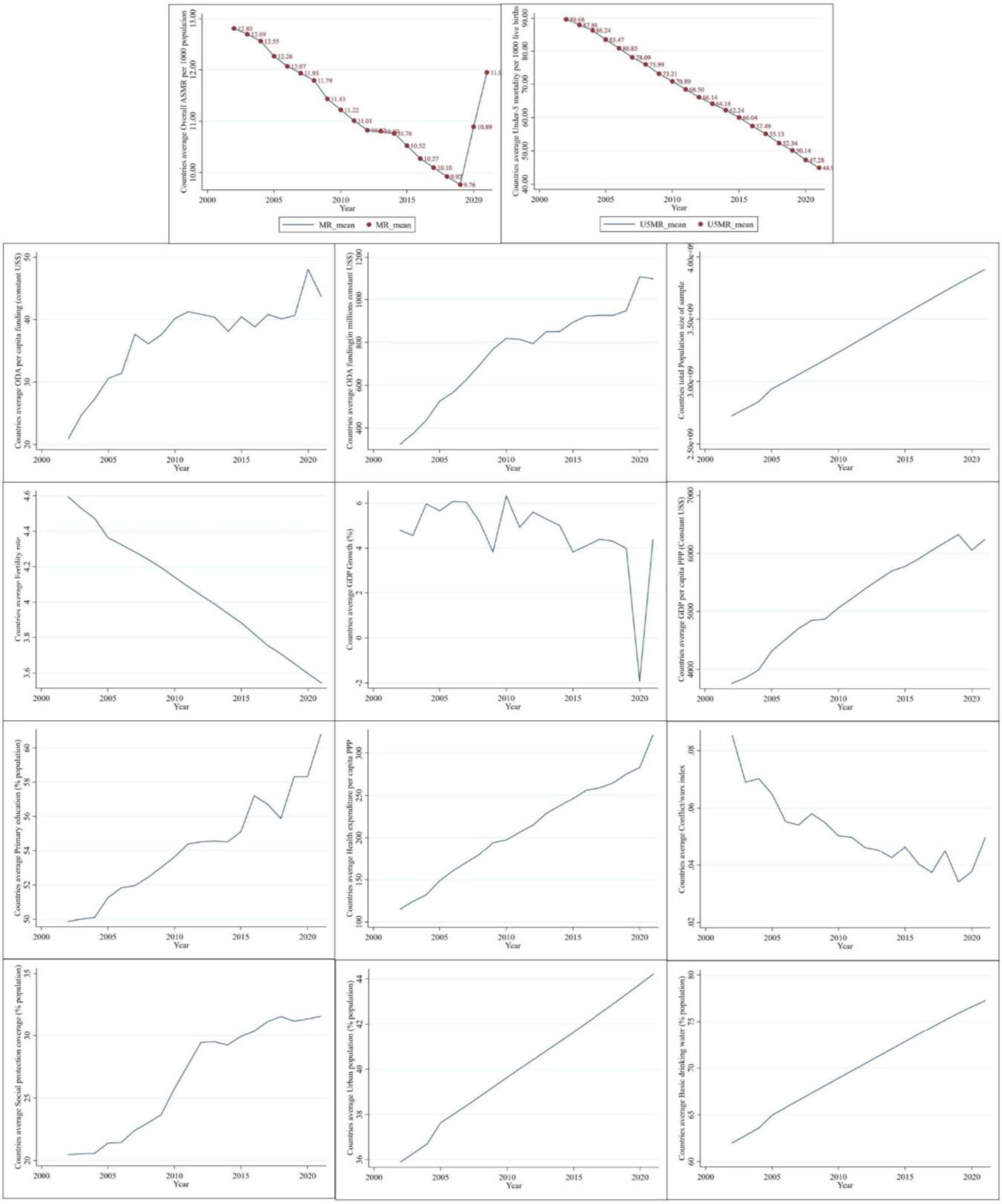

PART II - RETROSPECTIVE ANALYSIS

3. EMPIRICAL METHODS

3.1 Poisson Regression - Fixed effects

We use fixed-effect models with the Poisson approach, applying robust standard errors to assess both past and future effects of ODA amounts on health outcomes. The following equation illustrates the linear relationship between mortality rates and other covariates:

$$log\ (\mu_{it}) = \log(pop_{it}) + \sum \beta_q\ \mathrm{ODA}_{qit} + \sum \beta_s\ T_y + \sum \beta_k\ X_{kit} + \alpha_i + u_{it}$$

where:

• t denotes the year, i represents an individual country, and q indexes the categories of per capita ODA amounts.

• μ_{it} represents the expected number of events of interest— death count—observed in country i in year t. The death numbers used in this study are:

- ▪ Age-standardized (i.e., all ages and all causes) – main outcome,
- ▪ by different age groups, including child mortality (children under-5 per 1,000 live births), infancy (0 to 1 years old), preschool (2 to 4 years old, and school (age 5.9 years old).

• pop_{it} are the population of interest size in the time t for the observation i.

• ODAqit are the ODA per capita in categories (quintile, 5 groups) observed at the country i in year t, with associated coefficients β_q. The categories are defined as follows:

- ▪ q = 1: Extreme low or no ODA per capita (baseline).
- ▪ q = 2: Low (20th percentile).
- ▪ q = 3: Lower-intermediate (40th percentile or median).
- ▪ q = 4: Upper-intermediate (60th percentile).
- ▪ q = 5: High (80th percentile).

• T_y are dummy variables representing previous crisis events with coefficients from the specific years y, with y= 2009, 2015, 2020 and 2021.

• X_{kit} represents different control covariates, each one with a coefficient of β_k (economic downturns; gross domestic product (GDP) per capita at purchasing power parity (GDP pc PPP); armed conflict or wars index; fertility rate; urban population percentage; primary school attendance percentage; people using at least basic drinking water services percentage; health expenditure per capita (PPP); and, social protection coverage percentage.)

• α_i is the fixed effect (time-invariant) term for each country/territory, and u_{it} was the error term.

4. RESULTS

This section summarizes over ten sensitivity and triangulation analyses conducted to confirm our findings' robustness. These analyses were selected based on established causal inference principles in health research, particularly the Bradford Hill criteria.[14] In epidemiology, causality is most often assessed using these nine criteria: strength, consistency, specificity, temporality, biological gradient, plausibility, coherence, experiment, and analogy. Our analytical approach

was designed to maximize causal inference from observed statistical associations by addressing each criterion, as outlined in the WEB TABLE 2 below.

WEB TABLE 2 Analytical strategy used according to Bradford Hill`s criteria

Bradford Hill`s criteria		Analytical strategy used in the paper
**Criteria**	**Meaning**	
1. Strength of the association	The stronger the association between exposure and effect (e.g., high relative risk), the more likely it is to be causal.	The strong effect and statistical significance between high ODA per capita amounts and reductions in overall mortality ensure compliance with Bradford Hill's first criterion.
2. Consistency	If different studies, across various populations and settings, observe the same association, it strengthens the causal hypothesis.	To evaluate the external validity of our estimates, we refitted the model’s using data from 103 LMIC´s countries and territories (WEB TABLE 8). We also stratified the models by age groups (WEB TABLE 10). These heterogeneity analyses confirmed that the association between ODA disbursement and overall mortality reduction is broadly consistent across settings and population segments.<br>Moreover, to examine the stability of the results we develop triangulation analysis (Section 4.2 of this document). The direction and significance of the results remained consistent across models, which strengthens the case for a robust association.
3. Specificity	If a specific exposure leads to a single effect (or a limited number of effects), this supports causality.	The specificity of the models is also demonstrated by the differential effectiveness of ODA per capita on the different mortality causes, highest in the most expected causes such HIVAIDS, Tuberculosis, Neglected Tropical Diseases, among others, in comparison with the mortality for all causes. (WEB TABLE 14) Moreover, the lack of a statistically significant effect on specific causes, selected as negative controls, strengthens the specificity argument (WEB TABLE 17).
4. Temporality	The exposure must precede the effect in time (a fundamental principle for establishing causality).	The use of longitudinal panel data of two decades ensures the evaluation of temporal relationships. Furthermore, we tested alternative specifications for the time variables to assess whether the results were driven by temporal confounding or artefacts of model specification (WEB TABLE 18). The findings were stable across these variations, supporting temporal consistency.<br>These controls, combined with country fixed-effects models and other isolation factors, help refine the relationship between ODA per capita and changes in mortality over time.
5. Biological gradient	(Dose-Response Relationship) – If increased exposure levels correspond to a greater risk of the effect, this supports causality.	The choice of working with categorical variables enabled dose-response analysis between different levels of intervention (from lowest to highest) and mortality. The gradient (dose-response) seen in the effects of coverage levels for each one of the interventions in almost all considered outcomes.<br>Additionally, we estimated models using the continuous measure of ODA per capita disbursement (WEB TABLE 6). The consistency in direction and magnitude of the effects across both categorical and continuous specifications provides strong support for a dose response relationship, in

Bradford Hill`s criteria		Analytical strategy used in the paper
**Criteria**	**Meaning**	
		line with Bradford Hill's biological gradient criterion.
6. Plausibility	The proposed causal relationship should align with existing scientific knowledge and biological mechanisms.	The plausibility of ODA's impact on reducing mortality is outlined in the Research in Context and is well supported by existing literature, with several prior studies demonstrating similar effect.[15–24]
7. Coherence	The relationship should be consistent with what is already known about the disease or effect being studied.	This criterion refers to the replication of a causal association across different studies, populations, time periods, and methodological approaches. Numerous studies examining the relationship between ODA and similar health outcomes are cited in the Discussion section, further supporting this dimension of evidence.<br><br>By applying multiple analytical strategies, including alternative specifications of the exposure variable (from categorical levels to continuous measures), and triangulation using Difference in Difference (DiD) combined with Propensity Score Matching (PSM), we strengthen the coherence and consistency of our findings. The convergence of results across methods that rely on different assumptions and are subject to distinct sources of bias reinforces the overall causal interpretation.
8. Experiment	If experiments (such as clinical trials or natural experiments) confirm the association, this strengthens the causal inference.	While this criterion establishes that randomized experiments generally offer stronger causal inference than other study designs, it also acknowledges that even randomized studies may yield weak causal conclusions depending on their design specifications. More importantly, it does not exclude the possibility of deriving causal inference from quasi-experimental or observational designs. Carefully conducted, robust observational studies that adhere to causality criteria and include comprehensive sensitivity and triangulation analyses can provide a sufficient level of causal inference.[25]<br><br>However, our combination of DiD with PSM (Section 4.2 of this document) serves as a quasi-experimental approach, simulating conditions of a randomized controlled trial by reducing selection bias (through matching) and controlling for time-invariant unobserved confounding (through differencing). This strengthens the internal validity of the results and supports the causal interpretation of the association between ODA and mortality reduction.
9. Analogy	If a similar association has been demonstrated between another factor and a comparable effect, it may suggest causality.	The effects of ODA programs in other countries have been well documented in previous research, as summarized in the Research in Context section of the manuscript.<br><br>Our findings are further supported by the criterion of analogy, given that similar international aid initiatives

Bradford Hill`s criteria		Analytical strategy used in the paper
Criteria	Meaning	
		have shown beneficial impacts on population health outcomes in LMIC´s.[21] The consistent direction and magnitude of our estimates, confirmed through multiple robustness checks, including fixed-effects models, and DiD with PSM, parallel evidence from studies evaluating the effects of other targeted financial assistance programs on health indicators.<br><br>Moreover, the association we observe between higher ODA disbursements and lower overall mortality is analogous to the well-documented impacts of major global health initiatives such as the Global Fund and PEPFAR.[15,21] This alignment with established evidence from comparable large-scale development programs strengthens the plausibility and credibility of our conclusions.

4.1. Sensitivity Analysis

We conducted various sensitivity and auxiliary analyses to ensure the robustness of our findings.

1. We conducted the Hausmann test on overall mortality data and found that fixed effects model was better suited than random effects for analysing how ODA per capita quintiles affect mortality (see WEB TABLE 3).

2. We modify the model to assess pure regression without covariates and fixed effects (see WEB TABLE 4).

3. We tested models using various thresholds—dummy (median), tercile, quartile, and decile methods (WEB TABLE 5)—to assess how different categorizations affects results. We also used continuous variables and their logarithmic transformation (WEB TABLES 6 and 7), both of which produced consistent outcomes with the main model.

4. We tested external validity by refitting the models with the same covariates for 103 countries where data were available, including upper middle-income countries, SIDS, Syria, and South Sudan (see WEB TABLE 8).

5. We performed a series of heterogeneity analyses by disaggregating the results across a broader range of age groups (see WEB TABLE 10). We also conducted stratified analyses by sex (see WEB TABLE 11) and further stratified the females by age sub-groups (see Web Table 12).

6. We also stratified analyses in Sub-Saharan and non-Sub-Saharan countries (see WEB TABLE 13).

7. We conducted the model by different mortality causes as the outcome (see Web Table 14).

8. We analysed other ODA sectors subsets (see WEB TABLE 15), which are:

    a. Only Health
    b. Only Social infrastructure and services
    c. Only Social infrastructure and services and Humanitarian
    d. Only Social infrastructure and services, Multi-sector/Cross-cutting, Humanitarian, and Production sectors
    e. All sectors

9. Also, our main analysis ODA sector subset but given only with DAC country member donors (see WEB TABLE 16).

10. We have added a sensitivity analysis regarding negative controls in the main model, including deaths by transit injuries (see WEB TABLE 17)

11. We have added a sensitivity analysis regarding the main model with time trend alternative specifications (see WEB TABLE 18 and 19).

12. We have added different lag, duration and consolidation effect analyses. In the first one we regress ODA of different year lags. In the second one, duration effects, defined as the average ODA funding per capita in the previous x years. For example, the two-year duration effect would be the average ODA value per capita in the current and previous year, which then is assigned to the corresponding Baseline to High category. Third, consolidation year effects, for which we generate a 6th ODA category which represents the situation when the current ODA quintile is in the high category (i.e., ODA quintile=5) and if in the previous x years is also in the high category. For example, two years consolidation would be to have ODA quintile high in the current and previous year. This expresses the incremental effect of having been in the highest category for more than one year, thus a consolidation in the highest category. (see WEB TABLES 20, 21 and 22)

13. We have added sensitivity analyses related to health-related covariates: we specified a model without health covariate and with alternative ones, including Nurse rate. Hospital bed rate, Universal Health Coverage, Physicians rate, and Skilled birth attendance coverage (see WEB TABLE 23).

14. We have added sensitivity analyses of the main estimation by adjusting the model with several different covariates subsets (see WEB TABLE 24).

In sum, our results remained consistent throughout numerous sensitivity tests, confirming their robustness.

WEB TABLE 3 Hausman test between fixed effect and random effect Poisson models of main model

```
                 ---- Coefficients ----
             |      (b)          (B)            (b-B)     sqrt(diag(V_b-V_B))
             |     fixed        random       Difference          S.E.
-------------+----------------------------------------------------------------
         ODA |
          2  |  -.0798208    -.0798194       -1.41e-06        2.65e-07
          3  |  -.1601354    -.1601335       -1.93e-06        3.80e-07
          4  |  -.2310621    -.2310587       -3.34e-06        5.25e-07
          5  |   -.273248    -.2732429       -5.11e-06        6.56e-07
Economic_D~n |   .0026405     .0026412       -6.99e-07        1.23e-07
   GDP_Level |  -.0558549    -.0558556        7.21e-07        1.66e-07
Conflict_W~s |   .1813356     .1813355        1.69e-07        1.06e-06
Fertili~Rate |   .0277035     .0277047       -1.21e-06        5.77e-07
Urban_Popu~n |  -.0647648     -.064766        1.23e-06        8.46e-07
Primary_Ed~e |  -.0501861    -.0501865        4.09e-07        4.14e-07
Basic_Wate~e |  -.0258055     -.025808        2.49e-06        3.54e-07
Health_Exp~e |   -.057775    -.0577746       -4.35e-07        1.03e-07
Social_Pro~e |  -.0526208    -.0526204       -3.27e-07        2.17e-07
       y2009 |   .0340833     .0340831        1.95e-07        4.57e-08
       y2015 |  -.0224969     -.022497        3.35e-08        3.87e-08
       y2020 |   .0374201     .0374196        5.04e-07        1.08e-07
       y2021 |   .1273251     .1273252       -1.12e-07        1.09e-07
------------------------------------------------------------------------------
                          b = consistent under Ho and Ha; obtained from xtpoisson
           B = inconsistent under Ha, efficient under Ho; obtained from xtpoisson

    Test:  Ho:  difference in coefficients not systematic

                  chi2(17) = (b-B)'[(V_b-V_B)^(-1)](b-B)
                           =      167.83
                Prob>chi2 =      0.0000
                (V_b-V_B is not positive definite)
```

NOTE: Without implementing Robust Standard Errors

WEB TABLE 4 Rate Ratios from the Poisson models for the association between Overall ASMR and ODA quintiles (crude, country Fixed Effects, country-year Fixed Effects and fully adjusted), 2002-21

	(1)<br>Crude	(2)<br>Country FE	(3)<br>Country/Year FE	(4)<br>Fully Adjusted
Baseline (mean 6.38, 0-12.34)	1	1	1	1
	[1.000,1.000]	[1.000,1.000]	[1.000,1.000]	[1.000,1.000]
Low (mean 18.68, 12.35-24.40)	1.071***	0.884***	0.879***	0.923***
	[1.018,1.126]	[0.832,0.940]	[0.824,0.937]	[0.890,0.958]
Lower Intermediate (mean 30.21, 24.41-35.97)	1.222***	0.819***	0.809***	0.852***
	[1.174,1.272]	[0.738,0.909]	[0.727,0.900]	[0.778,0.933]
Upper Intermediate (mean 42.81, 35.98-52.12)	1.251***	0.742***	0.732***	0.794***
	[1.202,1.301]	[0.665,0.829]	[0.655,0.818]	[0.720,0.875]
High (mean 83.03, 52.13 or more)	1.347***	0.712***	0.700***	0.761***
	[1.282,1.415]	[0.620,0.818]	[0.610,0.803]	[0.668,0.866]
y2009			1.028***	1.035***
			[1.009,1.047]	[1.015,1.055]
y2015			0.956***	0.978
			[0.935,0.977]	[0.948,1.008]
y2020			1.001	1.038**
			[0.978,1.025]	[1.003,1.075]
y2021			1.098***	1.136***
			[1.064,1.132]	[1.104,1.168]
Economic downturn				1.003
				[0.963,1.044]
GDP per capita				0.946***
				[0.912,0.981]
Conflict/wars index				1.199***
				[1.054,1.364]
Fertility rate				1.028
				[0.986,1.072]
Urban population				0.937**
				[0.881,0.997]
Primary education				0.951***
				[0.919,0.984]
Basic drinking water				0.975

				[0.934,1.017]
Health expenditure				0.944***
				[0.911,0.978]
Social protection coverage				0.949
				[0.891,1.010]
_cons	0.0106***			
	[0.010,0.011]			
N	1177	1177	1177	1177
N_g		59	59	59

y2009= year dummy 2009… y2021= year dummy 2021
N = number of observations; *N_g* = number of countries
95% confidence intervals in brackets
* $p < 0.10$, ** $p < 0.05$, *** $p < 0.01$

WEB TABLE 5 Rate Ratios from the Fixed Effects Poisson models for the association between Overall ASMR and ODA by different ODA specifications (median, terciles, quartiles, quintiles, deciles), 2002-21

	-1	-2	-3	-4	-5
	Median	Terciles	Quartiles	Quintiles	Deciles
1.value_percapita_dic	1				
	[1.000,1.000]				
2.value_percapita_dic	0.895***				
	[0.847,0.947]				
1.value_percapita_ter		1			
		[1.000,1.000]			
2.value_percapita_ter		0.906**			
		[0.838,0.979]			
3.value_percapita_ter		0.837***			
		[0.759,0.923]			
1.value_percapita_qua			1		
			[1.000,1.000]		
2.value_percapita_qua			0.944***		
			[0.905,0.986]		
3.value_percapita_qua			0.857***		
			[0.795,0.924]		
4.value_percapita_qua			0.814***		
			[0.733,0.903]		
1.value_percapita_qui				1	
				[1.000,1.000]	
2.value_percapita_qui				0.923***	
				[0.890,0.958]	
3.value_percapita_qui				0.852***	
				[0.778,0.933]	
4.value_percapita_qui				0.794***	
				[0.720,0.875]	
5.value_percapita_qui				0.761***	
				[0.668,0.866]	
1.value_percapita_dec					1
					[1.000,1.000]

2.value_percapita_dec					0.986
					[0.946,1.027]
3.value_percapita_dec					0.915***
					[0.867,0.966]
4.value_percapita_dec					0.904***
					[0.853,0.957]
5.value_percapita_dec					0.857***
					[0.783,0.938]
6.value_percapita_dec					0.815***
					[0.734,0.905]
7.value_percapita_dec					0.785***
					[0.704,0.874]
8.value_percapita_dec					0.761***
					[0.675,0.858]
9.value_percapita_dec					0.739***
					[0.641,0.852]
10.value_percapita_dec					0.738***
					[0.622,0.876]
Economic downturn	1.009	1	1.002	1.003	1.001
	[0.966,1.054]	[0.962,1.040]	[0.962,1.044]	[0.963,1.044]	[0.962,1.041]
GDP per capita	0.930***	0.934***	0.936***	0.946***	0.947***
	[0.884,0.979]	[0.889,0.981]	[0.894,0.980]	[0.912,0.981]	[0.915,0.981]
Conflict/wars index	1.168**	1.199***	1.151**	1.199***	1.167**
	[1.020,1.337]	[1.066,1.348]	[1.010,1.313]	[1.054,1.364]	[1.020,1.335]
Fertility rate	1.024	1.018	1.026	1.028	1.025
	[0.977,1.074]	[0.978,1.059]	[0.988,1.066]	[0.986,1.072]	[0.982,1.069]
Urban population	0.915***	0.941	0.930**	0.937**	0.941*
	[0.858,0.975]	[0.869,1.019]	[0.869,0.994]	[0.881,0.997]	[0.883,1.003]
Primary education	0.933***	0.945***	0.940***	0.951***	0.951***
	[0.901,0.965]	[0.918,0.972]	[0.910,0.970]	[0.919,0.984]	[0.921,0.981]
Basic drinking water	0.936**	0.945*	0.943*	0.975	0.975
	[0.883,0.993]	[0.887,1.006]	[0.888,1.000]	[0.934,1.017]	[0.934,1.018]
Health expenditure	0.954**	0.953**	0.953**	0.944***	0.945***
	[0.914,0.995]	[0.913,0.995]	[0.913,0.994]	[0.911,0.978]	[0.910,0.980]
Social protection coverage	0.942	0.941*	0.941*	0.949	0.949
	[0.874,1.016]	[0.878,1.009]	[0.875,1.011]	[0.891,1.010]	[0.891,1.011]
y2009	1.032***	1.031***	1.030***	1.035***	1.035***
	[1.013,1.051]	[1.011,1.051]	[1.010,1.051]	[1.015,1.055]	[1.017,1.054]
y2015	0.975	0.972*	0.975	0.978	0.978
	[0.943,1.007]	[0.940,1.005]	[0.943,1.007]	[0.948,1.008]	[0.949,1.009]
y2020	1.029	1.038**	1.037*	1.038**	1.041**
	[0.989,1.070]	[1.001,1.076]	[1.000,1.075]	[1.003,1.075]	[1.006,1.077]
y2021	1.130***	1.132***	1.132***	1.136***	1.137***
	[1.101,1.160]	[1.102,1.163]	[1.102,1.162]	[1.104,1.168]	[1.105,1.171]
N	1177	1177	1177	1177	1177
N_g	59	59	59	59	59

MEDIAN: 1.value_percapita_dic= lower, 2.value_percapita_dic = upper

TERCILE: 1.value_percapita_ter= tercile 1,…, 3.value_percapita_ter= tercile 3
QUARTILE: 1.value_percapita_qua= quartile1,…, 4.value_percapita_qua= quartile 4.
QUINTILE: 1.value_percapita_qui = quintile 1,…, 5.value_percapita_qui = quintile 5
DECILE: 1.value_percapita_dec= decile 1,…, 10.value_percapita_dec=decile 10
y2009= year dummy 2009… y2021= year dummy 2021
N = number of observations; *N_g* = number of countries
95% confidence intervals in brackets
* $p < 0.10$, ** $p < 0.05$, *** $p < 0.01$

WEB TABLE 6 Rate Ratios from the Poisson models for the association between Overall ASMR and ODA value per capita (crude, Country-year Fixed Effects, fully adjusted), 2002-21

	(1)<br>Crude	(2)<br>Fixed Effects	(3)<br>Fully Adjusted
ODA per capita	1.004***	0.995***	0.998***
	[1.003,1.005]	[0.992,0.999]	[0.997,0.999]
y2009		1.031***	1.006
		[1.015,1.047]	[0.999,1.013]
y2015		0.950***	1.003
		[0.929,0.971]	[0.988,1.019]
y2020		0.993	1.109***
		[0.970,1.016]	[1.068,1.150]
y2021		1.086***	1.246***
		[1.057,1.116]	[1.200,1.294]
Economic downturn			1.018
			[0.984,1.054]
GDP per capita			1.000
			[1.000,1.000]
Conflict/wars index			1.112**
			[1.004,1.232]
Fertility rate			1.127***
			[1.061,1.196]
Urban population			0.987***
			[0.978,0.996]
Primary education			1.000
			[0.999,1.001]
Basic drinking water			0.999
			[0.994,1.004]
Health expenditure			1.000
			[1.000,1.000]
Social protection coverage			1.000**
			[1.000,1.001]
_cons	0.0106***		
	[0.010,0.011]		
N	1177	1177	1177
N_g		59	59

y2009= year dummy 2009… y2021= year dummy 2021
N = number of observations; *N_g* = number of countries
95% confidence intervals in brackets
* $p < 0.10$, ** $p < 0.05$, *** $p < 0.01$

WEB TABLE 7 Rate Ratios from the Poisson models for the association between Overall ASMR and ODA logarithmic of value per capita (crude, Country-year Fixed Effects, fully adjusted), 2002-21

	(1)<br>Crude	(2)<br>Fixed Effects	(3)<br>Fully Adjusted
Log of ODA per capita	1.084***	0.872***	0.944***
	[1.071,1.097]	[0.827,0.920]	[0.917,0.972]

y2009		1.040***	1.011***
		[1.022,1.058]	[1.003,1.019]
y2015		0.958***	1.004
		[0.942,0.973]	[0.988,1.019]
y2020		1.003	1.107***
		[0.970,1.037]	[1.066,1.149]
y2021		1.102***	1.243***
		[1.060,1.145]	[1.197,1.292]
Economic downturn			1.017
			[0.981,1.054]
GDP per capita			1.000
			[1.000,1.000]
Conflict/wars index			1.100*
			[0.991,1.220]
Fertility rate			1.134***
			[1.069,1.202]
Urban population			0.989***
			[0.981,0.996]
Primary education			1.000
			[0.999,1.001]
Basic drinking water			0.999
			[0.995,1.004]
Health expenditure			1.000
			[1.000,1.000]
Social protection coverage			1.000**
			[1.000,1.001]
_cons	0.00960***		
	[0.009,0.010]		
N	1177	1177	1177
N_g		59	59

y2009= year dummy 2009… y2021= year dummy 2021

N = number of observations; *N_g* = number of countries

95% confidence intervals in brackets

* $p < 0.10$, ** $p < 0.05$, *** $p < 0.01$

WEB TABLE 8 Rate Ratios from the Fixed Effects Poisson models for the association between mortality and ODA quintiles, for Overall ASMR and under-5 children for 103 LMIC´s, 2002-21

	Overall - ASMR	Child – Under-5 years
1.ODA	1	1
	[1.000,1.000]	[0.796,0.981]
2.ODA	0.922***	0.883**
	[0.870,0.977]	[0.589,0.814]
3.ODA	0.843***	0.692***
	[0.762,0.933]	[0.525,0.776]
4.ODA	0.795***	0.638***
	[0.717,0.881]	[0.478,0.980]
5.ODA	0.860**	0.684**
	[0.740,1.000]	[0.478,0.980]
Economic downturn	1.036*	0.984
	[0.998,1.075]	[0.895,1.082]
GDP per capita	0.937***	0.820***
	[0.913,0.962]	[0.774,0.869]
Conflict/wars index	0.983	1.171
	[0.797,1.213]	[0.951,1.444]
Fertility rate	1.025	1.090***
	[0.991,1.062]	[1.050,1.131]
Urban population	0.879***	0.852***

	[0.839,0.922]	[0.791,0.918]
Primary education	0.958*	0.920*
	[0.917,1.002]	[0.837,1.012]
Basic drinking water	0.965**	0.856***
	[0.936,0.994]	[0.798,0.918]
Health expenditure	0.967*	0.853***
	[0.932,1.003]	[0.760,0.959]
Social protection coverage	0.964**	0.892***
	[0.931,0.998]	[0.833,0.955]
y2009	1.030	1.024
	[0.977,1.085]	[0.972,1.079]
y2015	0.967***	0.905***
	[0.943,0.992]	[0.860,0.952]
y2020	0.992	0.753***
	[0.945,1.042]	[0.710,0.798]
y2021	1.115***	0.728***
	[1.041,1.194]	[0.682,0.777]
N	2054	2054
N_g	103	103

1.ODA= quintile 1, 2.ODA= quintile 2, 3.ODA= quintile 3, 4.ODA= quintile 4, 5.ODA= quintile 5
y2009= year dummy 2009… y2021= year dummy 2021
N = number of observations; *N_g* = number of countries
95% confidence intervals in brackets
* $p < 0.10$, ** $p < 0.05$, *** $p < 0.01$

WEB TABLE 9 Rate Ratios from the Fixed Effects Poisson models for the association between mortality and ODA quintiles using different time Fixed Effects (Main model-specific year dummies, 5-year period dummy, 4-year period dummy, yearly dummies), 2002-21

	(1) Main model (specific year dummies)	(2) 5-year period dummy	(3) 4-year period dummy	(4) yearly dummies
Baseline (mean 6.38, 0-12.34)	1	1	1	1
	[1.000,1.000]	[1.000,1.000]	[1.000,1.000]	[1.000,1.000]
Low (mean 18.68, 12.35-24.40)	0.923***	0.953**	0.960**	0.961**
	[0.890,0.958]	[0.916,0.992]	[0.923,0.998]	[0.925,0.998]
Lower Intermediate (mean 30.21, 24.41-35.97)	0.852***	0.921**	0.927**	0.928**
	[0.778,0.933]	[0.861,0.986]	[0.867,0.992]	[0.868,0.991]
Upper Intermediate (mean 42.81, 35.98-52.12)	0.794***	0.873***	0.883***	0.884***
	[0.720,0.875]	[0.809,0.942]	[0.821,0.950]	[0.828,0.943]
High (mean 83.03, 52.13 or more)	0.761***	0.861***	0.863***	0.867***
	[0.668,0.866]	[0.787,0.943]	[0.792,0.941]	[0.805,0.934]
Economic downturn	1.003	1.037***	1.028**	1.013
	[0.963,1.044]	[1.012,1.061]	[1.004,1.052]	[0.988,1.038]
GDP per capita	0.946***	0.993	0.986	0.989
	[0.912,0.981]	[0.941,1.048]	[0.947,1.027]	[0.951,1.028]
Conflict/wars index	1.199***	1.241***	1.226***	1.110**
	[1.054,1.364]	[1.082,1.422]	[1.075,1.398]	[1.018,1.211]
Fertility rate	1.028	0.989	0.991	0.993
	[0.986,1.072]	[0.947,1.034]	[0.945,1.039]	[0.943,1.045]
Urban population	0.937**	0.993	0.992	0.970
	[0.881,0.997]	[0.925,1.065]	[0.931,1.058]	[0.922,1.020]
Primary education	0.951***	0.988	0.992	1.004
	[0.919,0.984]	[0.951,1.027]	[0.953,1.034]	[0.961,1.049]
Basic drinking water	0.975	0.996	0.995	1.000

	[0.934,1.017]	[0.954,1.040]	[0.953,1.040]	[0.955,1.048]
Health expenditure	0.944***	1.012	1.025*	1.035***
	[0.911,0.978]	[0.974,1.051]	[0.997,1.053]	[1.014,1.056]
Social protection coverage	0.949	0.975	0.974	0.975
	[0.891,1.010]	[0.922,1.030]	[0.924,1.026]	[0.926,1.026]
y2009	1.035***			
	[1.015,1.055]			
y2015	0.978			
	[0.948,1.008]			
y2020	1.038**			
	[1.003,1.075]			
y2021	1.136***			
	[1.104,1.168]			
1.year_group_5		1		
		[1.000,1.000]		
2.year_group_5		0.934***		
		[0.911,0.958]		
3.year_group_5		0.871***		
		[0.831,0.912]		
4.year_group_5		0.863***		
		[0.832,0.896]		
1.year_group_4			1	
			[1.000,1.000]	
2.year_group_4			0.952***	
			[0.932,0.972]	
3.year_group_4			0.886***	
			[0.856,0.917]	
4.year_group_4			0.845***	
			[0.804,0.887]	
5.year_group_4			0.862***	
			[0.831,0.895]	
y2002				1
				[1.000,1.000]
y2003				0.995
				[0.987,1.002]
y2004				0.987*
				[0.972,1.002]
y2005				0.965***
				[0.954,0.977]
y2006				0.954***
				[0.939,0.969]
y2007				0.950***
				[0.925,0.976]
y2008				0.940***
				[0.909,0.971]
y2009				0.903***
				[0.870,0.937]
y2010				0.889***
				[0.854,0.924]
y2011				0.871***
				[0.836,0.907]
y2012				0.856***
				[0.820,0.893]
y2013				0.859***
				[0.819,0.900]
y2014				0.852***
				[0.806,0.902]
y2015				0.836***
				[0.794,0.880]
y2016				0.817***
				[0.777,0.859]
y2017				0.802***
				[0.764,0.841]
y2018				0.789***
				[0.753,0.827]
y2019				0.776***
				[0.740,0.814]

y2020				0.864*** [0.822,0.908]
y2021				0.952** [0.909,0.996]
N	1177	1177	1177	1177
N_g	59	59	59	59

y2001= year dummy 2009… y2022= year dummy 2021; 1.year_group_5= 2002-06, 2.year_group_5= 2007-11, 3.year_group_5=2012-16, 4.year_group_5=2017-21; 1.year_group_5= 2002-05, 2.year_group_5= 2006-09, 3.year_group_5=2010-13, 4.year_group_5=2014-17, 5.year_group_5=2018-21

N = number of observations; *N_g* = number of countries

95% confidence intervals in brackets

* $p < 0.10$, ** $p < 0.05$, *** $p < 0.01$

WEB TABLE 10 Rate Ratios from the Fixed Effects Poisson models for the association between mortality and ODA quintiles by different age sub-groups (Neonatal - 0 to 28 days, Infancy - 0 to 1 year, Preschool - 2 to 4 years, Child – Under-5 years, School-a age - 5 to 9 years, Adult - 10 to 54 years, Elderly - 55+ years, Overall-ASMR), 2002-21

	(1) Neonatal - 0 to 28 days	(2) Infancy - 0 to 1 year	(3) Preschool - 2 to 4 years	(4) Child – Under-5 years	(5) School-age - 5 to 9 years	(6) Adult - 10 to 54 years	(7) Elderly - 55+ years	(8) Overall-ASMR
Baseline (mean 6.38, 0-12.34)	1 [1.000,1.000]	1 [1.000,1.000]	1 [1.000,1.000]	1 [1.000,1.000]	1 [1.000,1.000]	1 [1.000,1.000]	1 [1.000,1.000]	1 [1.000,1.000]
Low (mean 18.68, 12.35-24.40)	0.931*** [0.882,0.982]	0.928** [0.868,0.993]	0.908 [0.799,1.032]	0.921** [0.851,0.998]	0.921*** [0.870,0.974]	0.923** [0.863,0.987]	0.920*** [0.898,0.943]	0.923*** [0.890,0.958]
Lower Intermediate (mean 30.21, 24.41-35.97)	0.845*** [0.775,0.921]	0.800*** [0.705,0.906]	0.718*** [0.566,0.910]	0.772*** [0.662,0.902]	0.804*** [0.704,0.920]	0.795*** [0.703,0.900]	0.892*** [0.845,0.942]	0.852*** [0.778,0.933]
Upper Intermediate (mean 42.81, 35.98-52.12)	0.843*** [0.763,0.931]	0.765*** [0.664,0.882]	0.650*** [0.510,0.827]	0.723*** [0.611,0.855]	0.755*** [0.649,0.879]	0.663*** [0.572,0.769]	0.870*** [0.807,0.939]	0.794*** [0.720,0.875]
High (mean 83.03, 52.13 or more)	0.832** [0.700,0.989]	0.723*** [0.565,0.925]	0.569*** [0.392,0.827]	0.670*** [0.504,0.890]	0.728*** [0.576,0.920]	0.603*** [0.493,0.738]	0.852*** [0.783,0.928]	0.761*** [0.668,0.866]
Economic downturn	0.994 [0.937,1.055]	0.979 [0.912,1.051]	0.954 [0.840,1.085]	0.974 [0.895,1.060]	0.941 [0.851,1.041]	1.032 [0.971,1.096]	0.992 [0.965,1.020]	1.003 [0.963,1.044]
GDP per capita	0.958** [0.919,0.999]	0.952* [0.900,1.006]	0.870** [0.767,0.988]	0.936* [0.876,1.001]	0.824*** [0.779,0.873]	0.920*** [0.890,0.951]	0.983 [0.947,1.019]	0.946*** [0.912,0.981]
Conflict/wars index	1.220*** [1.063,1.399]	1.352*** [1.153,1.587]	1.514*** [1.166,1.965]	1.409*** [1.164,1.706]	1.531*** [1.238,1.893]	1.356** [1.068,1.722]	1.092 [0.945,1.263]	1.199*** [1.054,1.364]
Fertility rate	0.978 [0.911,1.050]	0.986 [0.890,1.094]	1.028 [0.822,1.285]	0.988 [0.867,1.126]	0.928 [0.815,1.056]	1.071 [0.977,1.174]	1.065*** [1.039,1.092]	1.028 [0.986,1.072]
Urban population	0.928 [0.849,1.015]	0.889* [0.777,1.017]	0.906 [0.738,1.112]	0.871* [0.756,1.003]	0.985 [0.830,1.169]	0.913 [0.809,1.030]	0.959 [0.877,1.048]	0.937** [0.881,0.997]
Primary education	0.851*** [0.763,0.949]	0.844*** [0.762,0.934]	0.819*** [0.710,0.944]	0.841*** [0.757,0.934]	0.884*** [0.811,0.964]	0.966 [0.899,1.038]	0.992 [0.961,1.024]	0.951*** [0.919,0.984]
Basic drinking water	0.958 [0.893,1.028]	0.943 [0.873,1.019]	0.886* [0.783,1.003]	0.912** [0.839,0.992]	0.977 [0.879,1.087]	0.974 [0.881,1.077]	0.949*** [0.917,0.982]	0.975 [0.934,1.017]
Health expenditure	0.878*** [0.835,0.924]	0.845*** [0.796,0.896]	0.721*** [0.609,0.854]	0.818*** [0.759,0.882]	0.742*** [0.643,0.856]	0.906*** [0.852,0.964]	0.971** [0.948,0.994]	0.944*** [0.911,0.978]
Social protection coverage	0.966 [0.899,1.037]	0.957 [0.878,1.043]	0.905 [0.780,1.050]	0.942 [0.852,1.042]	0.982 [0.876,1.102]	0.991 [0.909,1.081]	0.967 [0.928,1.008]	0.949 [0.891,1.010]
y2009	1.067*** [1.021,1.116]	1.078*** [1.022,1.138]	1.091 [0.980,1.214]	1.082** [1.012,1.158]	1.128** [1.001,1.271]	1.072*** [1.022,1.124]	1.013** [1.002,1.025]	1.035*** [1.015,1.055]
y2015	0.950*** [0.918,0.983]	0.926*** [0.888,0.966]	0.842*** [0.811,0.875]	0.902*** [0.864,0.941]	0.907*** [0.888,0.927]	0.955*** [0.931,0.980]	0.987 [0.970,1.005]	0.978 [0.948,1.008]
y2020	0.826*** [0.787,0.866]	0.782*** [0.740,0.826]	0.751*** [0.661,0.852]	0.772*** [0.721,0.825]	0.739*** [0.640,0.854]	0.961 [0.908,1.016]	1.100*** [1.063,1.138]	1.038** [1.003,1.075]
y2021	0.783*** [0.741,0.827]	0.721*** [0.680,0.765]	0.654*** [0.574,0.746]	0.702*** [0.655,0.753]	0.676*** [0.559,0.817]	1.060*** [1.023,1.097]	1.210*** [1.181,1.240]	1.136*** [1.104,1.168]
N	1177	1177	1177	1177	1177	1177	1177	1177
N_g	59	59	59	59	59	59	59	59

y2009= year dummy 2009… y2021= year dummy 2021

N = number of observations; *N_g* = number of countries

95% confidence intervals in brackets

* $p < 0.10$, ** $p < 0.05$, *** $p < 0.01$

WEB TABLE 11 Rate Ratios from the Fixed Effects Poisson models for the association between Overall ASMR and ODA quintiles by sex (Male and Female), 2002-21

	(1) Male	(2) Female
Baseline (mean 6.38, 0-12.34)	1	1
	[1.000,1.000]	[1.000,1.000]

Low (mean 18.68, 12.35-24.40)	0.973	0.977
	[0.911,1.040]	[0.919,1.038]
Lower Intermediate (mean 30.21, 24.41-35.97)	0.885*	0.867**
	[0.781,1.003]	[0.764,0.985]
Upper Intermediate (mean 42.81, 35.98-52.12)	0.811***	0.772***
	[0.702,0.936]	[0.665,0.895]
High (mean 83.03, 52.13 or more)	0.774**	0.727***
	[0.635,0.943]	[0.585,0.903]
Economic downturn	0.992	1.015
	[0.916,1.075]	[0.930,1.108]
GDP per capita	0.987	0.995
	[0.957,1.018]	[0.961,1.031]
Conflict/wars index	1.150	1.091
	[0.937,1.411]	[0.878,1.356]
Fertility rate	1.073*	1.092*
	[0.992,1.159]	[0.992,1.203]
Urban population	0.849***	0.847***
	[0.782,0.921]	[0.788,0.911]
Primary education	0.974	0.965
	[0.903,1.051]	[0.895,1.041]
Basic drinking water	0.897***	0.900***
	[0.827,0.974]	[0.835,0.969]
Health expenditure	0.969*	0.964**
	[0.939,1.001]	[0.934,0.995]
Social protection coverage	0.928	0.949
	[0.847,1.016]	[0.862,1.044]
y2009	1.016*	1.007
	[0.999,1.034]	[0.992,1.023]
y2015	0.960**	0.960**
	[0.929,0.992]	[0.922,1.000]
y2020	1.093**	1.017
	[1.008,1.184]	[0.935,1.105]
y2021	1.157***	1.058
	[1.068,1.252]	[0.980,1.143]
N	988	988
N_g	59	59

y2009= year dummy 2009… y2021= year dummy 2021

N = number of observations; *N_g* = number of countries

95% confidence intervals in brackets

* $p < 0.10$, ** $p < 0.05$, *** $p < 0.01$

WEB TABLE 12 Rate Ratios from the Fixed Effects Poisson models for the association between mortality and ODA quintiles by age-subgroups for females (Children and adolescents - 0 to 14 years, Youth and young adults - 15 to 29 years, Young to middle-age adults - 30 to to 44 years, Middle-age adults - 45 to 59, Elderly - 60+ years, Overall ASMR), 2002-21

	(1) Children and adolescents - 0 to 14 years	(2) Youth and young adults - 15 to 29 years	(3) Young to middle-age adults - 30 to 44 years	(4) Middle-age adults - 45 to 59	(5) Elderly - 60+ years	(6) Overall-ASMR
Baseline (mean 6.38, 0-12.34)	1	1	1	1	1	1
	[1.000,1.000]	[1.000,1.000]	[1.000,1.000]	[1.000,1.000]	[1.000,1.000]	[1.000,1.000]
Low (mean 18.68, 12.35-24.40)	0.962	0.963	0.927**	0.945**	0.924***	0.977
	[0.868,1.066]	[0.908,1.023]	[0.861,0.999]	[0.899,0.993]	[0.887,0.962]	[0.919,1.038]
Lower Intermediate (mean 30.21, 24.41-35.97)	0.855*	0.832***	0.753***	0.845***	0.908***	0.867**
	[0.716,1.021]	[0.726,0.954]	[0.649,0.874]	[0.755,0.945]	[0.854,0.964]	[0.764,0.985]

Upper Intermediate (mean 42.81, 35.98-52.12)	0.782**	0.689***	0.544***	0.728***	0.882***	0.772***
	[0.636,0.963]	[0.576,0.826]	[0.449,0.659]	[0.635,0.835]	[0.821,0.948]	[0.665,0.895]
High (mean 83.03, 52.13 or more)	0.719*	0.622***	0.492***	0.683***	0.855***	0.727***
	[0.504,1.026]	[0.467,0.830]	[0.360,0.673]	[0.561,0.830]	[0.781,0.936]	[0.585,0.903]
Economic downturn	0.913	1.055	1.103	1.027	1.000	1.015
	[0.790,1.055]	[0.940,1.184]	[0.969,1.254]	[0.949,1.111]	[0.969,1.032]	[0.930,1.108]
GDP per capita	0.883***	0.839***	0.896***	0.942**	1.009	0.995
	[0.836,0.932]	[0.800,0.880]	[0.850,0.944]	[0.899,0.987]	[0.975,1.045]	[0.961,1.031]
Conflict/wars index	1.391***	1.291**	1.335*	1.080	1.054	1.091
	[1.166,1.659]	[1.051,1.586]	[0.985,1.808]	[0.889,1.313]	[0.869,1.277]	[0.878,1.356]
Fertility rate	0.974	1.027	1.181**	1.136***	1.049***	1.092*
	[0.906,1.047]	[0.960,1.098]	[1.011,1.379]	[1.046,1.234]	[1.014,1.085]	[0.992,1.203]
Urban population	0.850***	0.884*	0.817**	0.897**	0.982	0.847***
	[0.793,0.911]	[0.773,1.011]	[0.688,0.971]	[0.811,0.991]	[0.906,1.064]	[0.788,0.911]
Primary education	0.849***	0.945	0.968	0.977	0.982	0.965
	[0.763,0.944]	[0.870,1.028]	[0.869,1.077]	[0.932,1.024]	[0.953,1.011]	[0.895,1.041]
Basic drinking water	0.931	1.035	1.002	0.926*	0.952*	0.900***
	[0.852,1.017]	[0.934,1.148]	[0.905,1.108]	[0.854,1.004]	[0.901,1.006]	[0.835,0.969]
Health expenditure	0.756***	0.766***	0.858***	0.979	0.977**	0.964**
	[0.707,0.808]	[0.700,0.837]	[0.801,0.920]	[0.913,1.049]	[0.959,0.995]	[0.934,0.995]
Social protection coverage	0.937	1.001	0.983	0.986	0.972	0.949
	[0.835,1.051]	[0.889,1.128]	[0.866,1.116]	[0.906,1.073]	[0.934,1.010]	[0.862,1.044]
y2009	1.089*	1.117*	1.093***	0.992	1.013*	1.007
	[0.993,1.194]	[0.989,1.262]	[1.022,1.170]	[0.951,1.035]	[0.999,1.027]	[0.992,1.023]
y2015	0.858***	0.874***	0.901***	0.990	0.983*	0.960**
	[0.806,0.912]	[0.849,0.901]	[0.843,0.963]	[0.931,1.053]	[0.964,1.002]	[0.922,1.000]
y2020	0.698***	0.742***	0.835***	1.046	1.057***	1.017
	[0.613,0.796]	[0.660,0.835]	[0.737,0.945]	[0.970,1.129]	[1.022,1.094]	[0.935,1.105]
y2021	0.628***	0.799***	0.921	1.105***	1.120***	1.058
	[0.587,0.673]	[0.758,0.843]	[0.831,1.019]	[1.056,1.155]	[1.082,1.159]	[0.980,1.143]
N	985	988	988	988	988	988
N_g	59	59	59	59	59	59

y2009= year dummy 2009… y2021= year dummy 2021

N = number of observations; *N_g* = number of countries

95% confidence intervals in brackets

* $p < 0.10$, ** $p < 0.05$, *** $p < 0.01$

WEB TABLE 13 Rate Ratios from the Fixed Effects Poisson models for the association between mortality and ODA quintiles by regions (Sub-Saharan and non-Sub-Saharan countries), 2002-21

ODA Quintiles	Overall (ASMR)			Child (< 5 years)		
	All Countries Sample	SSAA Countries	Non-SSAA Countries	All Countries Sample	SSAA Countries	Non-SSAA Countries
Baseline (mean 6.38, 0-12.34)	1	1	1	1	1	1
Low (mean 18.68, 12.35-24.40)	0.92 (0.89-0.96; p=0.000)	0.93 (0.87-1.00; p=0.039)	0.91 (0.87-0.96; p=0.000)	0.92 (0.85-1.00; p=0.045)	0.95 (0.83-1.08; p=0.429)	0.90 (0.83-0.96; p=0.021)
Lower Intermediate (mean 30.21, 24.41-35.97)	0.85 (0.78-0.93; p=0.001)	0.83 (0.73-0.93; p=0.002)	0.93 (0.88-0.98; p=0.006)	0.77 (0.66-0.90; p=0.001)	0.77 (0.64-0.94; p=0.009)	0.86 (0.75-0.99; p=0.033)
Upper Intermediate (mean 42.81, 35.98-52.12)	0.79 (0.72-0.88; p=0.000)	0.75 (0.67-0.83; p=0.000)	0.95 (0.87-1.03; p=0.210)	0.72 (0.61-0.86; p=0.00)	0.70 (0.58-0.85; p=0.000)	0.93 (0.72-1.21; p=0.612)
High (mean 83.03, 52.13 or more)	0.76 (0.67-0.87; p=0.000)	0.70 (0.61-0.80 p=0.000)	0.94 (0.85-1.04; p=0.230)	0.67 (0.50-0.89; p=0.006)	0.60 (0.47-0.77; p=0.000)	1.06 (0.83-1.38; p=0.618)
**Number of observations**	1177	640	537	1177	640	537
**Number of countries**	59	32	27	59	32	27

Data are rate ratio coefficients (95% CI). ASMR=age-standardised mortality rate. ODA=Official Development Assistance. SSAA=Sub-Saharan.

WEB TABLE 14 Rate Ratios from the Fixed Effects Poisson models for the association between Overall ASMR and ODA quintiles by mortality causes (Tuberculosis, HIV/AIDS, Lower Respiratory Infections, Nutritional Deficiencies, Diarrhoeal Diseases, Neglected Tropical Diseases, Malaria), 2002-21

	Tuberculosis	HIV/AIDS	Lower Respiratory Infections	Nutritional Deficiencies	Diarrhoeal Diseases	Neglected Tropical Diseases	Malaria
Baseline (mean 6.38, 0-12.34)	1	1	1	1	1	1	1
	[1.000,1.000]	[1.000,1.000]	[1.000,1.000]	[1.000,1.000]	[1.000,1.000]	[1.000,1.000]	[1.000,1.000]
Low (mean 18.68, 12.35-24.40)	0.905**	0.835***	0.916**	0.862	0.939	0.881*	0.883*
	[0.831,0.986]	[0.752,0.928]	[0.844,0.994]	[0.676,1.100]	[0.797,1.105]	[0.768,1.010]	[0.771,1.011]
Lower Intermediate (mean 30.21, 24.41-35.97)	0.765**	0.585***	0.771***	0.645**	0.705***	0.718***	0.714***
	[0.619,0.946]	[0.485,0.707]	[0.659,0.903]	[0.432,0.962]	[0.542,0.916]	[0.582,0.885]	[0.585,0.871]
Upper Intermediate (mean 42.81, 35.98-52.12)	0.768**	0.366***	0.720***	0.604**	0.641***	0.582***	0.569***
	[0.609,0.969]	[0.298,0.449]	[0.616,0.841]	[0.403,0.904]	[0.488,0.843]	[0.461,0.735]	[0.454,0.713]
High (mean 83.03, 52.13 or more)	0.723**	0.295***	0.678***	0.556**	0.587***	0.513***	0.484***
	[0.555,0.942]	[0.217,0.402]	[0.545,0.844]	[0.347,0.892]	[0.410,0.839]	[0.380,0.691]	[0.364,0.644]
Economic downturn	0.998	1.180***	0.991	0.949	0.926	0.965	0.965
	[0.892,1.117]	[1.053,1.321]	[0.917,1.072]	[0.784,1.148]	[0.813,1.054]	[0.873,1.066]	[0.871,1.069]
GDP per capita	0.877***	0.857**	0.936*	0.747***	0.910***	0.879**	0.861**
	[0.838,0.918]	[0.735,1.000]	[0.874,1.003]	[0.659,0.846]	[0.848,0.976]	[0.773,0.999]	[0.746,0.994]
Conflict/wars index	1.195	2.331***	1.326***	1.547*	1.468**	1.827***	1.826***
	[0.937,1.525]	[1.470,3.698]	[1.095,1.605]	[0.960,2.494]	[1.089,1.978]	[1.331,2.506]	[1.352,2.467]
Fertility rate	1.050	1.066	1.051	1.038	1.225***	1.412***	1.439***
	[0.926,1.191]	[0.840,1.351]	[0.952,1.161]	[0.800,1.347]	[1.089,1.379]	[1.165,1.711]	[1.178,1.759]
Urban population	0.867**	0.682**	0.852***	0.867	0.842	0.881	0.901
	[0.761,0.987]	[0.494,0.941]	[0.779,0.931]	[0.688,1.092]	[0.677,1.046]	[0.744,1.043]	[0.760,1.069]
Primary education	0.885**	0.908	0.863***	0.833*	0.848	0.790***	0.786***
	[0.788,0.993]	[0.763,1.080]	[0.794,0.937]	[0.687,1.010]	[0.693,1.038]	[0.696,0.896]	[0.703,0.879]
Basic drinking water	0.849***	0.950	0.866***	0.964	0.716***	0.893*	0.873**
	[0.788,0.915]	[0.827,1.092]	[0.800,0.937]	[0.757,1.227]	[0.637,0.804]	[0.792,1.007]	[0.775,0.984]
Health expenditure	0.824***	0.865	0.892***	0.756***	0.720***	0.899**	0.897*
	[0.768,0.883]	[0.671,1.117]	[0.836,0.952]	[0.618,0.924]	[0.680,0.762]	[0.813,0.995]	[0.800,1.004]
Social protection coverage	0.885**	1.057	0.896*	0.816*	0.885**	0.960	0.975
	[0.783,1.000]	[0.872,1.282]	[0.795,1.010]	[0.662,1.006]	[0.787,0.996]	[0.823,1.119]	[0.837,1.136]
y2009	1.092**	1.228***	1.043*	1.105	1.177***	1.134***	1.145***
	[1.015,1.175]	[1.104,1.366]	[0.996,1.093]	[0.950,1.284]	[1.052,1.316]	[1.062,1.212]	[1.069,1.226]
y2015	0.919***	0.799***	0.943**	0.880***	0.853***	0.847***	0.834***
	[0.884,0.956]	[0.741,0.862]	[0.899,0.988]	[0.825,0.939]	[0.761,0.957]	[0.768,0.934]	[0.754,0.922]
y2020	0.817***	0.587***	0.776***	0.675***	0.757***	0.914	0.939
	[0.747,0.894]	[0.491,0.703]	[0.731,0.823]	[0.577,0.790]	[0.680,0.842]	[0.798,1.048]	[0.814,1.082]
y2021	0.789***	0.550***	0.720***	0.579***	0.664***	0.834***	0.853**
	[0.747,0.833]	[0.447,0.678]	[0.674,0.769]	[0.501,0.670]	[0.615,0.716]	[0.738,0.943]	[0.748,0.973]
N	1177	1177	1177	1177	1177	1177	1060
N_g	59	59	59	59	59	59	53

y2009= year dummy 2009… y2021= year dummy 2021

N = number of observations; *N_g* = number of countries

95% confidence intervals in brackets

* $p < 0.10$, ** $p < 0.05$, *** $p < 0.01$

WEB TABLE 15 Rate Ratios from the Fixed Effects Poisson models for the association between mortality and ODA quintiles (for Overall ASMR and children under-5) by different sectors, 2002-21

		Health				Social infrastructure and services				Social infrastructure and services + Humanitarian			
		RR	p-value	Lower	Upper	RR	p-value	Lower	Upper	RR	p-value	Lower	Upper
Overall (ASMR)	ODA per capita quintile												
	Low	0.94	0.00	0.91	0.97	0.92	0.00	0.88	0.96	0.94	0.00	0.91	0.98
	Intermediate	0.89	0.00	0.82	0.95	0.85	0.00	0.78	0.93	0.87	0.00	0.80	0.95
	High	0.82	0.00	0.76	0.88	0.77	0.00	0.70	0.85	0.82	0.00	0.75	0.89
	Extreme high	0.77	0.00	0.71	0.84	0.72	0.00	0.64	0.80	0.77	0.00	0.68	0.88
	Economic downturn	1.01	0.80	0.96	1.05	1.00	0.80	0.96	1.03	1.00	0.87	0.96	1.04
	GDP per capita	0.94	0.00	0.90	0.98	0.95	0.00	0.91	0.98	0.94	0.01	0.90	0.98
	Conflict/wars index	1.12	0.04	1.01	1.24	1.09	0.25	0.94	1.26	1.19	0.01	1.05	1.35
	Fertility rate	1.01	0.72	0.96	1.06	1.01	0.78	0.96	1.05	1.02	0.28	0.98	1.07
	Urban population	0.97	0.45	0.90	1.05	0.95	0.12	0.88	1.01	0.93	0.03	0.87	0.99
	Primary education	0.95	0.00	0.91	0.98	0.95	0.00	0.92	0.98	0.95	0.00	0.92	0.98
	Basic drinking water	0.96	0.16	0.90	1.02	0.96	0.16	0.92	1.01	0.96	0.13	0.92	1.01
	Health expenditure	0.95	0.04	0.91	1.00	0.95	0.01	0.91	0.98	0.95	0.01	0.91	0.98
	Social protection coverage	0.95	0.13	0.89	1.02	0.95	0.11	0.89	1.01	0.95	0.10	0.88	1.01
Child (< 5 years)	ODA per capita quintile	RR	p-value	Lower	Upper	RR	p-value	Lower	Upper	RR	p-value	Lower	Upper
	Low	0.91	0.00	0.86	0.96	0.91	0.01	0.85	0.98	0.94	0.12	0.88	1.01
	Intermediate	0.83	0.00	0.75	0.91	0.78	0.00	0.69	0.87	0.81	0.00	0.70	0.93
	High	0.70	0.00	0.62	0.79	0.69	0.00	0.61	0.77	0.75	0.00	0.65	0.87
	Extreme high	0.62	0.00	0.54	0.72	0.59	0.00	0.50	0.68	0.69	0.00	0.54	0.88
	Economic downturn	0.98	0.70	0.89	1.08	0.96	0.39	0.89	1.05	0.98	0.65	0.90	1.07
	GDP per capita	0.93	0.04	0.86	1.00	0.94	0.12	0.87	1.02	0.93	0.05	0.87	1.00
	Conflict/wars index	1.20	0.03	1.02	1.41	1.19	0.11	0.96	1.47	1.40	0.00	1.16	1.69
	Fertility rate	0.96	0.57	0.84	1.10	0.96	0.54	0.85	1.09	0.98	0.81	0.86	1.12
	Urban population	0.94	0.45	0.82	1.10	0.88	0.17	0.73	1.06	0.87	0.09	0.74	1.02
	Primary education	0.86	0.01	0.76	0.96	0.84	0.00	0.76	0.93	0.84	0.00	0.76	0.93
	Basic drinking water	0.91	0.04	0.83	1.00	0.90	0.02	0.83	0.98	0.90	0.02	0.82	0.98
	Health expenditure	0.83	0.00	0.76	0.91	0.82	0.00	0.76	0.89	0.82	0.00	0.76	0.89
	Social protection coverage	0.94	0.22	0.85	1.04	0.94	0.24	0.85	1.04	0.94	0.24	0.85	1.04

		Social infrastructure and services + Multi-sector/Cross-cutting + Humanitarian (main model)				Social infrastructure and services + Multi-sector/Cross-cuttin + Humanitarian + Production sectors				All sectors			
		RR	p-value	Lower	Upper	RR	p-value	Lower	Upper	RR	p-value	Lower	Upper
Overall (ASMR)	ODA per capita quintile												
	Low	0.92	0.00	0.89	0.96	0.93	0.00	0.90	0.96	0.96	0.1	0.92	1.01
	Intermediate	0.85	0.00	0.78	0.93	0.85	0.00	0.78	0.92	0.93	0.1	0.86	1.02
	High	0.79	0.00	0.72	0.88	0.79	0.00	0.72	0.87	0.94	0.2	0.87	1.02
	Extreme high	0.76	0.00	0.67	0.87	0.76	0.00	0.68	0.86	0.98	0.5	0.91	1.05
	Economic downturn	1.00	0.90	0.96	1.04	1.00	0.88	0.96	1.04	1.01	0.6	0.97	1.06
	GDP per capita	0.95	0.00	0.91	0.98	0.95	0.00	0.91	0.98	0.93	0.0	0.89	0.98
	Conflict/wars index	1.20	0.01	1.05	1.36	1.18	0.01	1.04	1.35	1.20	0.0	1.06	1.35
	Fertility rate	1.03	0.20	0.99	1.07	1.03	0.22	0.98	1.07	1.03	0.2	0.98	1.09
	Urban population	0.94	0.04	0.88	1.00	0.93	0.02	0.87	0.99	0.92	0.0	0.86	0.99
	Primary education	0.95	0.00	0.92	0.98	0.95	0.01	0.92	0.99	0.94	0.0	0.90	0.97
	Basic drinking water	0.97	0.23	0.93	1.02	0.97	0.23	0.93	1.02	0.94	0.0	0.88	1.00
	Health expenditure	0.94	0.00	0.91	0.98	0.94	0.00	0.91	0.98	0.95	0.0	0.91	0.99
	Social protection coverage	0.95	0.10	0.89	1.01	0.95	0.10	0.89	1.01	0.94	0.1	0.87	1.01
Child (< 5 years)	ODA per capita quintile	RR	p-value	Lower	Upper	RR	p-value	Lower	Upper	RR	p-value	Lower	Upper
	Low	0.92	0.05	0.85	1.00	0.93	0.09	0.85	1.01	0.99	0.8	0.89	1.09
	Intermediate	0.77	0.00	0.66	0.90	0.76	0.00	0.66	0.87	0.96	0.6	0.81	1.13
	High	0.72	0.00	0.61	0.86	0.70	0.00	0.61	0.81	1.00	1.0	0.86	1.16
	Extreme high	0.67	0.01	0.50	0.89	0.66	0.00	0.51	0.86	1.03	0.7	0.89	1.20
	Economic downturn	0.97	0.53	0.89	1.06	0.96	0.28	0.90	1.03	1.00	1.0	0.91	1.10
	GDP per capita	0.94	0.05	0.88	1.00	0.94	0.08	0.87	1.01	0.92	0.0	0.87	0.98
	Conflict/wars index	1.41	0.00	1.16	1.71	1.35	0.00	1.14	1.59	1.41	0.0	1.13	1.75
	Fertility rate	0.99	0.86	0.87	1.13	0.98	0.79	0.87	1.11	0.99	0.9	0.86	1.14
	Urban population	0.87	0.06	0.76	1.00	0.85	0.06	0.72	1.01	0.85	0.1	0.69	1.03
	Primary education	0.84	0.00	0.76	0.93	0.85	0.00	0.76	0.94	0.82	0.0	0.75	0.90
	Basic drinking water	0.91	0.03	0.84	0.99	0.91	0.02	0.83	0.98	0.87	0.0	0.80	0.95
	Health expenditure	0.82	0.00	0.76	0.88	0.82	0.00	0.76	0.89	0.83	0.0	0.77	0.90
	Social protection coverage	0.94	0.25	0.85	1.04	0.95	0.25	0.86	1.04	0.92	0.2	0.82	1.04

All models included 59 countries and 1177 observations

WEB TABLE 16 Rate Ratios from the Fixed Effects Poisson models for the association between mortality and ODA quintiles (for Overall ASMR and children under-5) for DAC country members donors, 2002-21

		Official Donors (main model)				DAC countries			
		RR	p-value	Lower	Upper	RR	p-value	Lower	Upper
Overall (ASMR)	**ODA per capita quintile**								
	**Low**	0.92	0.00	0.89	0.96	0.97	0.04	0.94	1.00
	**Intermediate**	0.85	0.00	0.78	0.93	0.90	0.01	0.84	0.97
	**High**	0.79	0.00	0.72	0.88	0.86	0.00	0.80	0.93
	**Extreme high**	0.76	0.00	0.67	0.87	0.83	0.00	0.75	0.91
	**Economic downturn**	1.00	0.90	0.96	1.04	1.01	0.69	0.96	1.06
	**GDP per capita**	0.95	0.00	0.91	0.98	0.94	0.00	0.89	0.98
	**Conflict/wars index**	1.20	0.01	1.05	1.36	1.23	0.00	1.08	1.39
	**Fertility rate**	1.03	0.20	0.99	1.07	1.03	0.20	0.98	1.08
	**Urban population**	0.94	0.04	0.88	1.00	0.93	0.02	0.87	0.99
	**Primary education**	0.95	0.00	0.92	0.98	0.94	0.00	0.91	0.97
	**Basic drinking water**	0.97	0.23	0.93	1.02	0.95	0.08	0.90	1.01
	**Health expenditure**	0.94	0.00	0.91	0.98	0.95	0.02	0.91	0.99
	**Social protection coverage**	0.95	0.10	0.89	1.01	0.94	0.10	0.87	1.01
Child (< 5 years)	**ODA per capita quintile**	RR	p-value	Lower	Upper	RR	p-value	Lower	Upper
	**Low**	0.92	0.05	0.85	1.00	0.95	0.13	0.89	1.02
	**Intermediate**	0.77	0.00	0.66	0.90	0.83	0.01	0.73	0.95
	**High**	0.72	0.00	0.61	0.86	0.83	0.01	0.72	0.95
	**Extreme high**	0.67	0.01	0.50	0.89	0.78	0.03	0.62	0.98
	**Economic downturn**	0.97	0.53	0.89	1.06	0.99	0.76	0.90	1.08
	**GDP per capita**	0.94	0.05	0.88	1.00	0.93	0.02	0.87	0.99
	**Conflict/wars index**	1.41	0.00	1.16	1.71	1.43	0.00	1.18	1.72
	**Fertility rate**	0.99	0.86	0.87	1.13	0.99	0.92	0.87	1.14
	**Urban population**	0.87	0.06	0.76	1.00	0.86	0.05	0.74	1.00
	**Primary education**	0.84	0.00	0.76	0.93	0.83	0.00	0.75	0.92
	**Basic drinking water**	0.91	0.03	0.84	0.99	0.89	0.01	0.82	0.97
	**Health expenditure**	0.82	0.00	0.76	0.88	0.82	0.00	0.76	0.89
	**Social protection coverage**	0.94	0.25	0.85	1.04	0.93	0.18	0.83	1.04
All models included 59 countries and 1177 observations									

WEB TABLE 17 Negative controls

	Transit injuries mortality
Baseline (mean 6.38, 0-12.34)	1
	[1.000,1.000]
Low (mean 18.68, 12.35-24.40)	0.992
	[0.925,1.064]
Lower Intermediate (mean 30.21, 24.41-35.97)	0.954
	[0.865,1.052]
Upper Intermediate (mean 42.81, 35.98-52.12)	0.929
	[0.842,1.026]
High (mean 83.03, 52.13 or more)	0.911
	[0.814,1.020]
Economic_Downturn	0.946**
	[0.896,1.000]
GDP_Level	1.060**
	[1.009,1.114]
Conflict_Wars	1.091
	[0.954,1.247]
Fertility_Rate	0.971
	[0.925,1.020]

Urban_Population	0.944
	[0.876,1.017]
Primary_Education_Coverage	0.951
	[0.884,1.022]
Basic_Water_Coverage	0.884***
	[0.836,0.935]
Health_Expenditure	0.961
	[0.902,1.023]
Social_Protection_Coverage	1.002
	[0.952,1.054]
y2009	1.029***
	[1.017,1.041]
y2015	0.962***
	[0.941,0.983]
y2020	0.946***
	[0.919,0.973]
y2021	0.906***
	[0.873,0.940]
N	1177
N_g	59

N = number of observations; *N_g* = number of countries
95% confidence intervals in brackets
* $p < 0.10$, ** $p < 0.05$, *** $p < 0.01$

WEB TABLE 18 Overall (ASMR) Mortality model with different time trend specifications

	(1) Main model (ODA in quintiles)	(2) 5-year grouping	(3) 4-year grouping	(4) All years year fixed effect dummies	(5) Year variable as continuous
Baseline (mean 6.38, 0-12.34)	1	1	1	1	1
	[1.000,1.000]	[1.000,1.000]	[1.000,1.000]	[1.000,1.000]	[1.000,1.000]
Low (mean 18.68, 12.35-24.40)	0.923***	0.953**	0.960**	0.961**	0.953**
	[0.890,0.958]	[0.916,0.992]	[0.923,0.998]	[0.925,0.998]	[0.918,0.989]
Lower Intermediate (mean 30.21, 24.41-35.97)	0.852***	0.921**	0.927**	0.928**	0.917**
	[0.778,0.933]	[0.861,0.986]	[0.867,0.992]	[0.868,0.991]	[0.853,0.986]
Upper Intermediate (mean 42.81, 35.98-52.12)	0.794***	0.873***	0.883***	0.884***	0.867***
	[0.720,0.875]	[0.809,0.942]	[0.821,0.950]	[0.828,0.943]	[0.799,0.942]
High (mean 83.03, 52.13 or more)	0.761***	0.861***	0.863***	0.867***	0.854***
	[0.668,0.866]	[0.787,0.943]	[0.792,0.941]	[0.805,0.934]	[0.772,0.946]
Economic_Downturn	1.003	1.037***	1.028**	1.013	1.050***
	[0.963,1.044]	[1.012,1.061]	[1.004,1.052]	[0.988,1.038]	[1.021,1.078]
GDP_Level	0.946***	0.993	0.986	0.989	0.969

	[0.912,0.981]	[0.941,1.048]	[0.947,1.027]	[0.951,1.028]	[0.927,1.012]
Conflict_Wars	1.199***	1.241***	1.226***	1.110**	1.265***
	[1.054,1.364]	[1.082,1.422]	[1.075,1.398]	[1.018,1.211]	[1.083,1.478]
Fertility_Rate	1.028	0.989	0.991	0.993	0.989
	[0.986,1.072]	[0.947,1.034]	[0.945,1.039]	[0.943,1.045]	[0.951,1.029]
Urban_Population	0.937**	0.993	0.992	0.970	0.995
	[0.881,0.997]	[0.925,1.065]	[0.931,1.058]	[0.922,1.020]	[0.920,1.076]
Primary_Education_Coverage	0.951***	0.988	0.992	1.004	0.986
	[0.919,0.984]	[0.951,1.027]	[0.953,1.034]	[0.961,1.049]	[0.952,1.020]
Basic_Water_Coverage	0.975	0.996	0.995	1.000	1.001
	[0.934,1.017]	[0.954,1.040]	[0.953,1.040]	[0.955,1.048]	[0.959,1.045]
Health_Expenditure	0.944***	1.012	1.025*	1.035***	1.012
	[0.911,0.978]	[0.974,1.051]	[0.997,1.053]	[1.014,1.056]	[0.971,1.054]
Social_Protection_Coverage	0.949	0.975	0.974	0.975	0.977
	[0.891,1.010]	[0.922,1.030]	[0.924,1.026]	[0.926,1.026]	[0.925,1.032]
y2009	1.035***				
	[1.015,1.055]				
y2015	0.978				
	[0.948,1.008]				
y2020	1.038**				
	[1.003,1.075]				
y2021	1.136***				
	[1.104,1.168]				
1.year_group_5		1			
		[1.000,1.000]			
2.year_group_5		0.934***			
		[0.911,0.958]			
3.year_group_5		0.871***			
		[0.831,0.912]			
4.year_group_5		0.863***			
		[0.832,0.896]			
1.year_group_4			1		
			[1.000,1.000]		
2.year_group_4			0.952***		

	[0.932,0.972]	
3.year_group_4	0.886***	
	[0.856,0.917]	
4.year_group_4	0.845***	
	[0.804,0.887]	
5.year_group_4	0.862***	
	[0.831,0.895]	
2002.year		1
		[1.000,1.000]
2003.year		0.995
		[0.987,1.002]
2004.year		0.987*
		[0.972,1.002]
2005.year		0.965***
		[0.954,0.977]
2006.year		0.954***
		[0.939,0.969]
2007.year		0.950***
		[0.925,0.976]
2008.year		0.940***
		[0.909,0.971]
2009.year		0.903***
		[0.870,0.937]
2010.year		0.889***
		[0.854,0.924]
2011.year		0.871***
		[0.836,0.907]
2012.year		0.856***
		[0.820,0.893]
2013.year		0.859***
		[0.819,0.900]
2014.year		0.852***
		[0.806,0.902]
2015.year		0.836***
		[0.794,0.880]
2016.year		0.817***

				[0.777,0.859]	
2017.year				0.802***	
				[0.764,0.841]	
2018.year				0.789***	
				[0.753,0.827]	
2019.year				0.776***	
				[0.740,0.814]	
2020.year				0.864***	
				[0.822,0.908]	
2021.year				0.952**	
				[0.909,0.996]	
year					0.991***
					[0.988,0.994]
N	1177	1177	1177	1177	1177
N_g	59	59	59	59	59

N = number of observations; *N_g* = number of countries
95% confidence intervals in brackets
* $p < 0.10$, ** $p < 0.05$, *** $p < 0.01$

WEB TABLE 19 Overall (ASMR) Mortality model continuous exposition and year adjusting variable

	(1)<br>Main model (continuous)	(2)<br>With continuous adjusting year variable
ODA value per capita	0.998***	0.998***
	[0.997,0.999]	[0.997,0.999]
Economic_Downturn	1.018	1.054***
	[0.984,1.054]	[1.024,1.085]
GDP_pc_ppp	1.000	1.000
	[1.000,1.000]	[1.000,1.000]
Conflict_Wars	1.112**	1.240***
	[1.004,1.232]	[1.063,1.446]
Fertility_rate	1.127***	1.038
	[1.061,1.196]	[0.950,1.136]

URBAN_f	0.987***	0.997
	[0.978,0.996]	[0.986,1.007]
educ_primary	1.000	1.001
	[0.999,1.001]	[0.999,1.002]
water_basid	0.999	0.998
	[0.994,1.004]	[0.993,1.003]
SHXPDCHEXPPCD_i_ma3	1.000	1.000
	[1.000,1.000]	[1.000,1.001]
social_prot_cov	1.000**	1.000
	[1.000,1.001]	[1.000,1.001]
y2009	1.006	
	[0.999,1.013]	
y2015	1.003	
	[0.988,1.019]	
y2020	1.109***	
	[1.068,1.150]	
y2021	1.246***	
	[1.200,1.294]	
year		0.993**
		[0.986,1.000]
N	1177	1177
N_g	59	59

y2009= year dummy 2009… y2021= year dummy 2021
N = number of observations; N_g = number of countries
95% confidence intervals in brackets
* $p < 0.10$, ** $p < 0.05$, *** $p < 0.01$

WEB TABLE 20 Overall (ASMR) Mortality model with year lag effects

	(1) Main model	(2) 1 year lag	(3) 2-year lag	(4) 3-year lag	(5) 4-year lag	(6) 5-year lag
Baseline (mean 6.38, 0-12.34)	1	1	1	1	1	1
	[1.000,1.000]	[1.000,1.000]	[1.000,1.000]	[1.000,1.000]	[1.000,1.000]	[1.000,1.000]
Low (mean 18.68, 12.35-24.40)	0.923***	0.934***	0.936***	0.940***	0.959***	0.972
	[0.890,0.958]	[0.904,0.965]	[0.911,0.961]	[0.918,0.962]	[0.937,0.983]	[0.937,1.009]
Lower Intermediate (mean 30.21, 24.41-35.97)	0.852***	0.866***	0.871***	0.886***	0.910***	0.927**
	[0.778,0.933]	[0.796,0.942]	[0.805,0.943]	[0.825,0.952]	[0.849,0.976]	[0.866,0.992]
	0.794***	0.804***	0.812***	0.822***	0.851***	0.881***

Upper Intermediate (mean 42.81, 35.98-52.12)	[0.720,0.875]	[0.731,0.884]	[0.739,0.893]	[0.753,0.896]	[0.794,0.913]	[0.822,0.945]
High (mean 83.03, 52.13 or more)	0.761***	0.778***	0.776***	0.796***	0.833***	0.869***
	[0.668,0.866]	[0.690,0.879]	[0.687,0.878]	[0.717,0.884]	[0.760,0.913]	[0.799,0.945]
Economic_Downturn	1.003	0.993	0.974*	0.986	0.985	0.985
	[0.963,1.044]	[0.956,1.031]	[0.948,1.000]	[0.957,1.015]	[0.958,1.013]	[0.960,1.010]
GDP_Level	0.946***	0.944**	0.947**	0.948*	0.947	0.932*
	[0.912,0.981]	[0.903,0.987]	[0.898,1.000]	[0.892,1.007]	[0.885,1.013]	[0.859,1.013]
Conflict_Wars	1.199***	1.177***	1.204***	1.166***	1.183***	1.198***
	[1.054,1.364]	[1.043,1.328]	[1.077,1.346]	[1.047,1.298]	[1.070,1.309]	[1.073,1.338]
Fertility_Rate	1.028	1.027	1.019	1.005	0.997	0.997
	[0.986,1.072]	[0.991,1.064]	[0.984,1.054]	[0.967,1.044]	[0.958,1.037]	[0.960,1.034]
Urban_Population	0.937**	0.943*	0.966	0.977	0.991	0.999
	[0.881,0.997]	[0.883,1.007]	[0.909,1.027]	[0.918,1.040]	[0.932,1.054]	[0.940,1.061]
Primary_Education_Coverage	0.951***	0.951***	0.952***	0.953***	0.951***	0.956***
	[0.919,0.984]	[0.922,0.982]	[0.922,0.983]	[0.925,0.982]	[0.924,0.979]	[0.931,0.982]
Basic_Water_Coverage	0.975	0.967	0.961	0.961	0.953*	0.947*
	[0.934,1.017]	[0.919,1.018]	[0.908,1.018]	[0.911,1.014]	[0.903,1.006]	[0.896,1.001]
Health_Expenditure	0.944***	0.951**	0.952*	0.953*	0.949**	0.943**
	[0.911,0.978]	[0.910,0.994]	[0.904,1.002]	[0.903,1.006]	[0.901,0.999]	[0.897,0.992]
Social_Protection_Coverage	0.949	0.955	0.965	0.963	0.967	0.979
	[0.891,1.010]	[0.899,1.015]	[0.908,1.026]	[0.913,1.016]	[0.919,1.017]	[0.934,1.025]
y2009	1.035***	1.031***	1.037***	1.038***	1.044***	1.049***
	[1.015,1.055]	[1.011,1.051]	[1.020,1.053]	[1.022,1.054]	[1.031,1.058]	[1.039,1.060]
y2015	0.978	0.973	0.986	0.979	0.987	0.993
	[0.948,1.008]	[0.940,1.007]	[0.963,1.009]	[0.952,1.006]	[0.965,1.009]	[0.976,1.012]
y2020	1.038**	1.037**	1.055***	1.049***	1.055***	1.056***
	[1.003,1.075]	[1.001,1.074]	[1.030,1.081]	[1.025,1.074]	[1.029,1.082]	[1.035,1.078]
y2021	1.136***	1.138***	1.131***	1.135***	1.139***	1.143***
	[1.104,1.168]	[1.105,1.171]	[1.103,1.160]	[1.105,1.167]	[1.107,1.172]	[1.108,1.179]
N	1177	1118	1059	1000	941	882
N_g	59	59	59	59	59	59

y2009= year dummy 2009… y2021= year dummy 2021

N = number of observations; *N_g* = number of countries

95% confidence intervals in brackets

* $p < 0.10$, ** $p < 0.05$, *** $p < 0.01$

WEB TABLE 21 Overall (ASMR) Mortality model with consolidation effects

	(1) Main model	(2) 1-year consolidation	(3) 2-year consolidation	(4) 3-year consolidation	(5) 4-year consolidation
Baseline (mean 6.38, 0-12.34)	1	1	1	1	1
	[1.000,1.000]	[1.000,1.000]	[1.000,1.000]	[1.000,1.000]	[1.000,1.000]
Low (mean 18.68, 12.35-24.40)	0.923***	0.923***	0.923***	0.922***	0.922***
	[0.890,0.958]	[0.889,0.957]	[0.889,0.957]	[0.889,0.957]	[0.889,0.957]
Lower Intermediate (mean 30.21, 24.41-35.97)	0.852***	0.852***	0.851***	0.851***	0.851***
	[0.778,0.933]	[0.778,0.932]	[0.777,0.932]	[0.777,0.932]	[0.777,0.932]
Upper Intermediate (mean 42.81, 35.98-52.12)	0.794***	0.793***	0.793***	0.793***	0.793***
	[0.720,0.875]	[0.719,0.875]	[0.719,0.875]	[0.719,0.876]	[0.719,0.875]
High (mean 83.03, 52.13 or more)	0.761***	0.780***	0.776***	0.771***	0.767***
	[0.668,0.866]	[0.693,0.876]	[0.688,0.875]	[0.681,0.872]	[0.675,0.870]
Consolidation category		0.742***	0.730***	0.726***	0.728***
		[0.640,0.861]	[0.623,0.856]	[0.617,0.854]	[0.620,0.854]
Economic_Downturn	1.003	1.003	1.003	1.003	1.003
	[0.963,1.044]	[0.963,1.045]	[0.963,1.045]	[0.963,1.045]	[0.963,1.045]
GDP_Level	0.946***	0.945***	0.945***	0.945***	0.945***
	[0.912,0.981]	[0.912,0.981]	[0.911,0.980]	[0.911,0.980]	[0.911,0.980]
Conflict_Wars	1.199***	1.198***	1.198***	1.198***	1.198***
	[1.054,1.364]	[1.052,1.363]	[1.052,1.363]	[1.052,1.364]	[1.052,1.364]
Fertility_Rate	1.028	1.028	1.027	1.026	1.026
	[0.986,1.072]	[0.986,1.072]	[0.985,1.071]	[0.985,1.070]	[0.985,1.069]
Urban_Population	0.937**	0.938**	0.939**	0.938**	0.938**
	[0.881,0.997]	[0.883,0.998]	[0.882,0.999]	[0.881,0.999]	[0.881,0.999]
Primary_Education_Coverage	0.951***	0.952***	0.952***	0.952***	0.952***
	[0.919,0.984]	[0.919,0.986]	[0.919,0.986]	[0.919,0.985]	[0.919,0.985]
Basic_Water_Coverage	0.975	0.976	0.978	0.978	0.978

	[0.934,1.017]	[0.936,1.018]	[0.936,1.021]	[0.937,1.022]	[0.936,1.021]
Health_Expenditure	0.944***	0.944***	0.945***	0.944***	0.945***
	[0.911,0.978]	[0.911,0.979]	[0.911,0.980]	[0.911,0.979]	[0.911,0.979]
Social_Protection_Coverage	0.949	0.948*	0.948*	0.948*	0.948*
	[0.891,1.010]	[0.891,1.010]	[0.890,1.009]	[0.890,1.009]	[0.890,1.009]
y2009	1.035***	1.035***	1.035***	1.035***	1.035***
	[1.015,1.055]	[1.016,1.055]	[1.016,1.055]	[1.015,1.055]	[1.016,1.055]
y2015	0.978	0.977	0.977	0.978	0.978
	[0.948,1.008]	[0.947,1.008]	[0.947,1.008]	[0.948,1.008]	[0.948,1.008]
y2020	1.038**	1.037**	1.037**	1.038**	1.038**
	[1.003,1.075]	[1.001,1.073]	[1.002,1.074]	[1.002,1.075]	[1.002,1.076]
y2021	1.136***	1.136***	1.136***	1.136***	1.136***
	[1.104,1.168]	[1.104,1.168]	[1.104,1.168]	[1.104,1.169]	[1.104,1.170]
N	1177	1177	1177	1177	1177
N_g	59	59	59	59	59

y2009= year dummy 2009… y2021= year dummy 2021
N = number of observations; *N_g* = number of countries
95% confidence intervals in brackets
* $p < 0.10$, ** $p < 0.05$, *** $p < 0.01$

WEB TABLE 22 Overall (ASMR) Mortality model with year duration effects

	Main model	2 year duration	3 year duration	4 year duration	5 year duration	6 year duration
Baseline (mean 6.38, 0-12.34)	1.0000000	1.0000000	1.0000000	1.0000000	1.0000000	1.0000000
	[1.0000000,1.0000000]	[1.0000000,1.0000000]	[1.0000000,1.0000000]	[1.0000000,1.0000000]	[1.0000000,1.0000000]	[1.0000000,1.0000000]
Low (mean 18.68, 12.35-24.40)	0.9232818***	0.9339294***	0.9298706***	0.9312701***	0.9339590***	0.9457531***
	[0.8899598,0.9578514]	[0.8941668,0.9754602]	[0.8960523,0.9649653]	[0.9014346,0.9620931]	[0.9076824,0.9609963]	[0.9239975,0.9680208]
Lower Intermediate (mean 30.21, 24.41-35.97)	0.8520284***	0.8615017***	0.8546913***	0.8561374***	0.8574649***	0.8815729***
	[0.7782605,0.9327885]	[0.7910751,0.9381982]	[0.7854299,0.9300604]	[0.7878457,0.9303488]	[0.7971228,0.9223750]	[0.8229871,0.9443292]
Upper Intermediate (mean 42.81, 35.98-52.12)	0.7936902***	0.8079670***	0.8035718***	0.8115223***	0.8246076***	0.8487259***
	[0.7196989,0.8752884]	[0.7356490,0.8873943]	[0.7255660,0.8899640]	[0.7269456,0.9059391]	[0.7437105,0.9143043]	[0.7635515,0.9434015]
High (mean 83.03, 52.13 or more)	0.7609041***	0.7656342***	0.7563430***	0.7607156***	0.7671415***	0.7994823***
	[0.6684249,0.8661781]	[0.6682107,0.8772617]	[0.6552282,0.8730619]	[0.6600020,0.8767977]	[0.6642903,0.8859169]	[0.7008852,0.9119496]
Economic_Downturn	1.0026440	0.9955462	0.9762250	0.9736300**	0.9741208**	0.9763817**
	[0.9626850,1.0442616]	[0.9560200,1.0367065]	[0.9480274,1.0052613]	[0.9493969,0.9984817]	[0.9513907,0.9973940]	[0.9545240,0.9987398]
GDP_Level	0.9456763***	0.9447629***	0.9541095*	0.9559804	0.9564076	0.9437626
	[0.9117996,0.9808117]	[0.9052133,0.9860404]	[0.9095398,1.0008632]	[0.9045329,1.0103541]	[0.8985331,1.0180097]	[0.8703761,1.0233367]
Conflict_Wars	1.1988175***	1.1860832***	1.2021354***	1.1778979***	1.2046822***	1.1807479***
	[1.0535215,1.3641518]	[1.0479898,1.3423732]	[1.0684544,1.3525421]	[1.0523595,1.3184120]	[1.0844876,1.3381982]	[1.0718785,1.3006750]
Fertility_Rate	1.0280908	1.0247716	1.0212406	1.0171432	1.0112864	1.0029803
	[0.9858633,1.0721270]	[0.9845737,1.0666107]	[0.9785035,1.0658444]	[0.9722067,1.0641567]	[0.9686894,1.0557566]	[0.9613730,1.0463883]
Urban_Population	0.9372879**	0.9445208*	0.9621304	0.9690669	0.9903161	0.9977579
	[0.8812814,0.9968538]	[0.8841444,1.0090202]	[0.9041384,1.0238419]	[0.9133765,1.0281528]	[0.9359574,1.0478319]	[0.9420689,1.0567389]
Primary_Education_Coverage	0.9510524***	0.9519411***	0.9585586**	0.9626560**	0.9655772**	0.9652575**
	[0.9193114,0.9838894]	[0.9199583,0.9850358]	[0.9239410,0.9944732]	[0.9283050,0.9982780]	[0.9337014,0.9985411]	[0.9385543,0.9927205]
Basic_Water_Coverage	0.9745246	0.9674994	0.9714816	0.9729175	0.9754851	0.9636486
	[0.9341616,1.0166316]	[0.9213408,1.0159704]	[0.9268650,1.0182460]	[0.9302032,1.0175931]	[0.9347097,1.0180393]	[0.9187359,1.0107568]
Health_Expenditure	0.9438623***	0.9475941**	0.9456676**	0.9460843**	0.9427450**	0.9393306***
	[0.9107284,0.9782016]	[0.9088614,0.9879775]	[0.9030916,0.9902508]	[0.9011261,0.9932856]	[0.8992898,0.9882999]	[0.8967193,0.9839668]
Social_Protection_Coverage	0.9487397	0.9554561	0.9582668	0.9623924	0.9667942	0.9789967
	[0.8908448,1.0103972]	[0.8964354,1.0183627]	[0.9017120,1.0183687]	[0.9130668,1.0143827]	[0.9194391,1.0165882]	[0.9345764,1.0255283]
y2009	1.0346708***	1.0352352***	1.0343099***	1.0398244***	1.0461625***	1.0472772***
	[1.0151478,1.0545692]	[1.0168260,1.0539777]	[1.0165758,1.0523535]	[1.0242651,1.0556200]	[1.0326710,1.0598303]	[1.0345088,1.0602032]
y2015	0.9777542	0.9783685	0.9810610	0.9854099	0.9894846	0.9941121
	[0.9481250,1.0083094]	[0.9497720,1.0078260]	[0.9541624,1.0087178]	[0.9612718,1.0101541]	[0.9679772,1.0114698]	[0.9758761,1.0126888]
y2020	1.0381291**	1.0387850**	1.0527471***	1.0570806***	1.0609515***	1.0622405***
	[1.0025004,1.0750240]	[1.0022070,1.0766980]	[1.0219332,1.0844902]	[1.0320410,1.0827277]	[1.0383606,1.0840339]	[1.0410301,1.0838831]
y2021	1.1357862***	1.1359589***	1.1331401***	1.1357919***	1.1364558***	1.1424745***
	[1.1043400,1.1681277]	[1.1048967,1.1678943]	[1.1034019,1.1636799]	[1.1053306,1.1670927]	[1.1059660,1.1677861]	[1.1085749,1.1774106]
N	1177	1118	1059	1000	941	882
N_g	5.90e+01	5.90e+01	5.90e+01	5.90e+01	5.90e+01	5.90e+01

y2009= year dummy 2009… y2021= year dummy 2021
N = number of observations; *N_g* = number of countries
95% confidence intervals in brackets
* $p < 0.10$, ** $p < 0.05$, *** $p < 0.01$

WEB TABLE 23 Overall (ASMR) Mortality model with no health expenditure variable and with alternative health variables

	(1) Main model (MM)	(2) MM without health variable	(3) MM with nurse rate	(4) MM with bed rate	(5) MM with UHC index	(6) MM with physicians' rate	(7) MM with Skilled Birth Attendance Coverage
Baseline (mean 6.38, 0-12.34)	1 [1.000,1.000]	1 [1.000,1.000]	1 [1.000,1.000]	1 [1.000,1.000]	1 [1.000,1.000]	1 [1.000,1.000]	1 [1.000,1.000]
Low (mean 18.68, 12.35-24.40)	0.923*** [0.890,0.958]	0.935*** [0.901,0.970]	0.940*** [0.905,0.975]	0.935*** [0.901,0.970]	0.932*** [0.902,0.964]	0.932*** [0.899,0.965]	0.922*** [0.887,0.959]
Lower Intermediate (mean 30.21, 24.41-35.97)	0.852*** [0.778,0.933]	0.860*** [0.786,0.942]	0.869*** [0.790,0.957]	0.861*** [0.788,0.940]	0.863*** [0.787,0.948]	0.858*** [0.785,0.938]	0.860*** [0.783,0.945]
Upper Intermediate (mean 42.81, 35.98-52.12)	0.794*** [0.720,0.875]	0.803*** [0.728,0.885]	0.815*** [0.736,0.903]	0.801*** [0.726,0.883]	0.803*** [0.733,0.880]	0.801*** [0.727,0.883]	0.805*** [0.731,0.887]
High (mean 83.03, 52.13 or more)	0.761*** [0.668,0.866]	0.770*** [0.675,0.879]	0.782*** [0.683,0.895]	0.768*** [0.673,0.877]	0.778*** [0.696,0.870]	0.767*** [0.672,0.875]	0.779*** [0.693,0.875]
Economic_Downturn	1.003 [0.963,1.044]	0.998 [0.958,1.039]	0.996 [0.954,1.040]	0.999 [0.958,1.041]	0.992 [0.952,1.035]	1.004 [0.966,1.044]	1.002 [0.963,1.042]
GDP_Level	0.946*** [0.912,0.981]	0.910*** [0.896,0.924]	0.910*** [0.897,0.924]	0.913*** [0.898,0.929]	0.960** [0.929,0.991]	0.910*** [0.896,0.924]	0.940*** [0.918,0.962]
Conflict_Wars	1.199*** [1.054,1.364]	1.207*** [1.065,1.368]	1.194*** [1.047,1.362]	1.206*** [1.064,1.368]	1.168** [1.028,1.329]	1.192*** [1.048,1.355]	1.180*** [1.045,1.333]
Fertility_Rate	1.028 [0.986,1.072]	1.041* [0.996,1.087]	1.034 [0.991,1.079]	1.042* [0.996,1.091]	1.017 [0.982,1.054]	1.043* [0.998,1.090]	1.035** [1.001,1.070]
Urban_Population	0.937** [0.881,0.997]	0.938** [0.882,0.997]	0.939** [0.885,0.995]	0.931** [0.873,0.992]	0.929** [0.874,0.987]	0.942** [0.890,0.997]	0.941** [0.887,0.999]
Primary_Education_Coverage	0.951*** [0.919,0.984]	0.954*** [0.923,0.987]	0.952*** [0.919,0.985]	0.954*** [0.922,0.986]	0.965** [0.937,0.993]	0.962*** [0.935,0.989]	0.958*** [0.930,0.987]
Basic_Water_Coverage	0.975 [0.934,1.017]	0.967* [0.929,1.006]	0.969 [0.931,1.009]	0.970 [0.932,1.009]	0.972 [0.934,1.012]	0.970 [0.931,1.011]	0.963* [0.923,1.006]
Health_Expenditure	0.944*** [0.911,0.978]						
Social_Protection_Coverage	0.949 [0.891,1.010]	0.943** [0.893,0.997]	0.942** [0.891,0.997]	0.941** [0.887,0.997]	0.956 [0.896,1.019]	0.941** [0.891,0.994]	0.941* [0.883,1.002]
y2009	1.035*** [1.015,1.055]	1.028*** [1.014,1.043]	1.028*** [1.013,1.043]	1.028*** [1.012,1.044]	1.029*** [1.010,1.048]	1.024*** [1.007,1.042]	1.010 [0.993,1.027]
y2015	0.978 [0.948,1.008]	0.973** [0.949,0.999]	0.974** [0.949,0.999]	0.975* [0.950,1.001]	0.979 [0.953,1.006]	0.977* [0.953,1.001]	0.986 [0.953,1.020]
y2020	1.038** [1.003,1.075]	1.034* [0.997,1.072]	1.036* [0.998,1.075]	1.035* [0.998,1.073]	1.052*** [1.015,1.090]	1.033* [0.997,1.071]	1.047*** [1.012,1.083]
y2021	1.136*** [1.104,1.168]	1.125*** [1.089,1.163]	1.127*** [1.091,1.165]	1.128*** [1.092,1.165]	1.144*** [1.103,1.188]	1.131*** [1.092,1.171]	1.145*** [1.114,1.177]
Nurse_rate			0.959** [0.921,0.998]				
Hospital_bed_rate				0.973 [0.934,1.014]			
Universal_Health_Coveage					0.925*** [0.899,0.9		

					52]		
Physicians_rate						0.913***	
						[0.899,0.928]	
Skilled_birth_attendance_coverage							0.930***
							[0.911,0.949]
N	1177	1177	1177	1177	1177	1177	1177
N_g	59	59	59	59	59	59	59

y2009= year dummy 2009… y2021= year dummy 2021

N = number of observations; *N_g* = number of countries

95% confidence intervals in brackets

* $p < 0.10$, ** $p < 0.05$, *** $p < 0.01$

WEB TABLE 24 Overall (ASMR) Mortality model with alternative covariates sets

	Main model	Alt. Model 1	Alt. Model 2	Alt. Model 3	Alt. Model 4
Baseline (mean 6.38, 0-12.34)	1	1	1	1	1
	[1.000,1.000]	[1.000,1.000]	[1.000,1.000]	[1.000,1.000]	[1.000,1.000]
Low (mean 18.68, 12.35-24.40)	0.923***	0.935***	0.932***	0.916***	0.927***
	[0.890,0.958]	[0.899,0.973]	[0.900,0.964]	[0.881,0.952]	[0.893,0.962]
Lower Intermediate (mean 30.21, 24.41-35.97)	0.852***	0.864***	0.860***	0.840***	0.871***
	[0.778,0.933]	[0.784,0.952]	[0.783,0.944]	[0.770,0.918]	[0.791,0.959]
Upper Intermediate (mean 42.81, 35.98-52.12)	0.794***	0.805***	0.796***	0.771***	0.807***
	[0.720,0.875]	[0.727,0.892]	[0.725,0.874]	[0.700,0.849]	[0.736,0.886]
High (mean 83.03, 52.13 or more)	0.761***	0.771***	0.772***	0.737***	0.775***
	[0.668,0.866]	[0.674,0.881]	[0.690,0.862]	[0.651,0.834]	[0.695,0.864]
Economic_Downturn	1.003	0.999	0.994	1.006	1.003
	[0.963,1.044]	[0.959,1.042]	[0.956,1.034]	[0.967,1.046]	[0.961,1.046]
GDP_Level	0.946***	0.941***	0.981		
	[0.912,0.981]	[0.911,0.973]	[0.948,1.014]		
Conflict_Wars	1.199***	1.202***	1.166**	1.203**	1.206**
	[1.054,1.364]	[1.051,1.375]	[1.021,1.332]	[1.045,1.386]	[1.036,1.402]
Fertility_Rate	1.028	1.045*	1.031	1.072**	1.059***
	[0.986,1.072]	[0.993,1.101]	[0.984,1.080]	[1.004,1.145]	[1.029,1.089]
Urban_Population	0.937**	0.932**	0.922***	0.920**	0.942*
	[0.881,0.997]	[0.876,0.991]	[0.867,0.980]	[0.855,0.990]	[0.882,1.006]
Primary_Education_Coverage	0.951***	0.952***			
	[0.919,0.984]	[0.921,0.985]			
Basic_Water_Coverage	0.975	0.969	0.974		
	[0.934,1.017]	[0.928,1.013]	[0.937,1.013]		
Health_Expenditure	0.944***				
	[0.911,0.978]				
Social_Protection_Coverage	0.949	0.943*	0.950	0.935**	0.936**
	[0.891,1.010]	[0.888,1.000]	[0.890,1.014]	[0.883,0.990]	[0.884,0.991]
Gini_index		0.970*	0.977	0.968*	0.971**
		[0.936,1.005]	[0.945,1.011]	[0.935,1.002]	[0.945,0.998]
Sanitation_Coverage		0.973	0.993	0.945***	
		[0.931,1.017]	[0.954,1.033]	[0.909,0.982]	
Nurse_rate		0.956**			
		[0.920,0.994]			
Secondary_Education			0.946***	0.954**	
			[0.913,0.980]	[0.914,0.996]	
Universal_Health_Coverage			0.927***		
			[0.896,0.958]		
Physician_rate				0.918***	
				[0.901,0.935]	
Literacy_rate					0.940***
					[0.923,0.957]
Electricity_Coverage					0.902***

					[0.874,0.930]
Skilled_birth_attendance_Coverage					0.952***
					[0.920,0.984]
y2009	1.035***	1.014	1.024***	1.001	0.993
	[1.015,1.055]	[0.988,1.041]	[1.010,1.039]	[0.964,1.040]	[0.964,1.022]
y2015	0.978	0.978	0.981	0.979	0.989
	[0.948,1.008]	[0.947,1.009]	[0.952,1.011]	[0.948,1.010]	[0.939,1.041]
y2020	1.038**	1.043**	1.054***	1.041**	1.063***
	[1.003,1.075]	[1.005,1.082]	[1.017,1.093]	[1.002,1.083]	[1.028,1.099]
y2021	1.136***	1.137***	1.152***	1.144***	1.166***
	[1.104,1.168]	[1.108,1.168]	[1.110,1.196]	[1.109,1.180]	[1.138,1.195]
N	1177	1177	1177	1177	1177
N_g	59	59	59	59	59

y2009= year dummy 2009… y2021= year dummy 2021
N = number of observations; *N_g* = number of countries
95% confidence intervals in brackets
* $p < 0.10$, ** $p < 0.05$, *** $p < 0.01$

4.2. Triangulation

Difference-in-difference with Propensity Score Matching

We assessed the impact of ODA disbursements per capita on mortality outcomes using a Difference-in-Differences (DiD) design, supplemented by Propensity Score Matching (PSM) as an additional robustness check. To enhance comparability between groups at baseline, the analysis was limited to LIC countries. The final sample consisted of 340 countries/years, classified into two groups: those receiving none or low levels of ODA disbursements (n = 79, control group) and those receiving intermediate to high levels (n = 261, treatment group). Mortality indicators were examined for the years 2002 and 2019.

We selected 2002 as the baseline year because it corresponds to the first year with available ODA disbursement information, while 2019 was chosen to minimize potential distortions associated with COVID-19-related mortality trends. The definition of ODA per capita categories mirrors that used in our primary regression models: countries in the lowest two quintiles (none or low disbursements) were assigned to the control group (coded as 0), while countries in the third through fifth quintiles (intermediate to high and consolidated disbursements) were assigned to the treatment group (coded as 1).

WEB FIGURES 4 and 5 demonstrate that the crucial assumptions for PSM and DiD, common support and parallel trends, are met, thereby reinforcing confidence in the estimated treatment effects. Although the trajectories diverge in the later years, the early-period trends are highly similar between treatment and control countries, which supports the parallel trends assumption in the pre-treatment period.

WEB FIGURE 4 Evidence of common support in mortality rate distributions for treatment and control groups defined by ODA per capita disbursement levels.

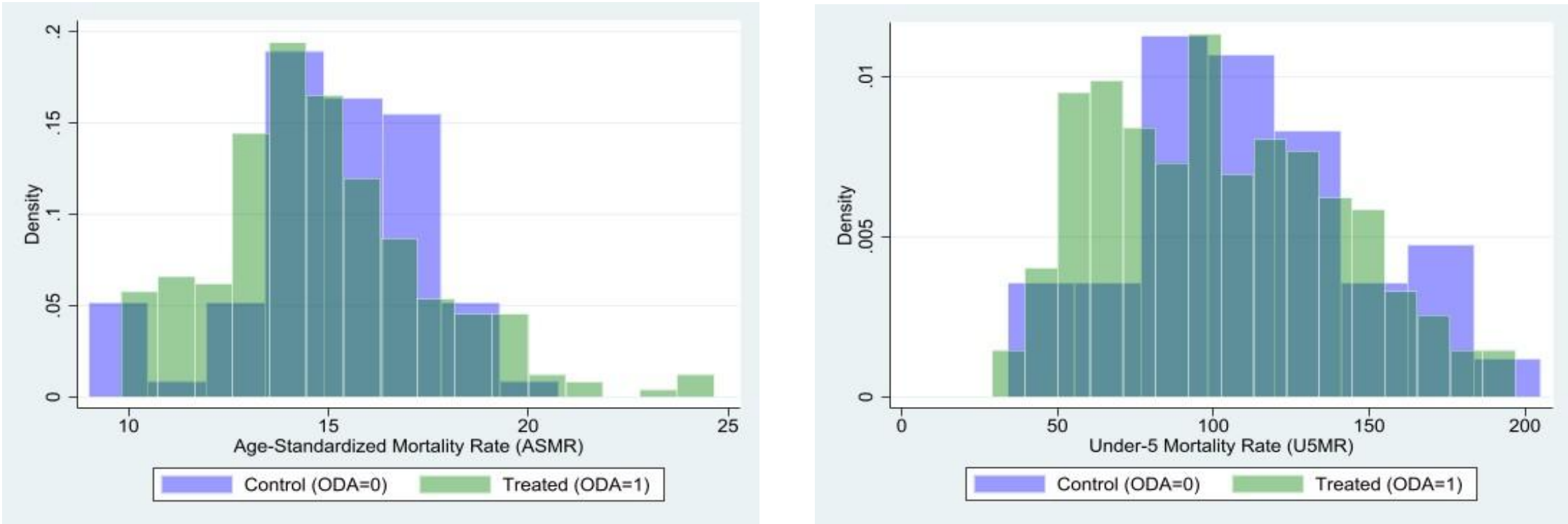

WEB FIGURE 5 Assessment of the parallel trends assumption in mortality rates between treatment and control groups defined by ODA per capita disbursement levels.

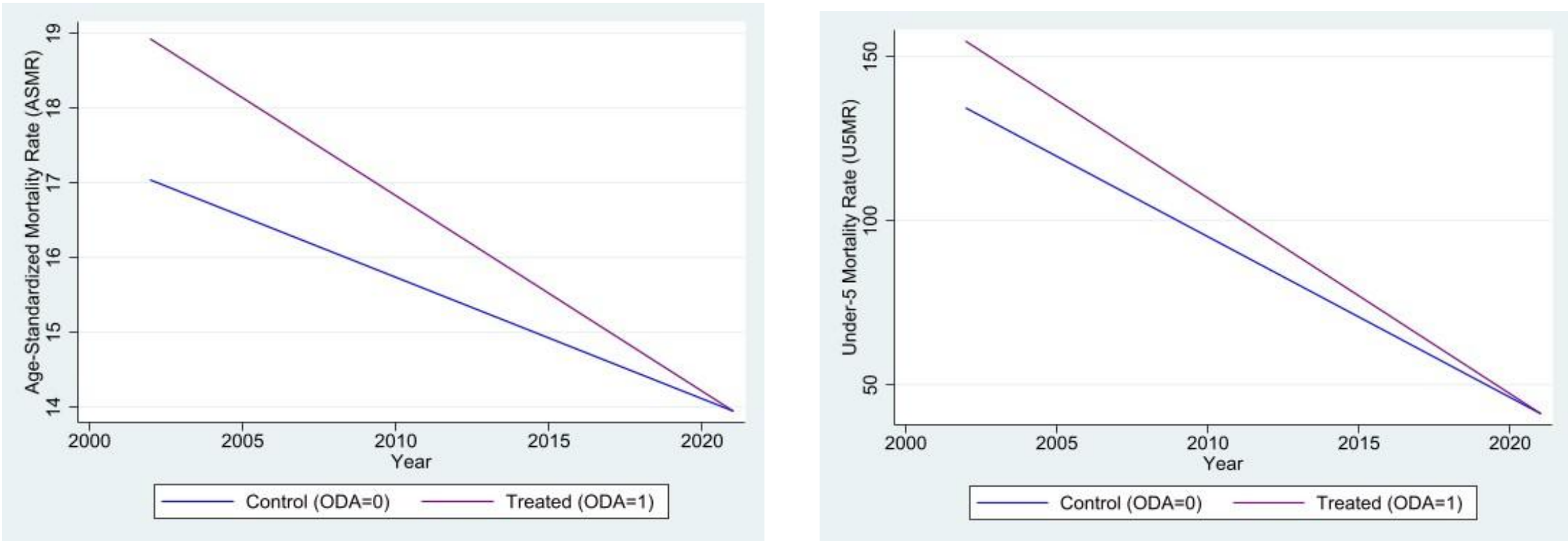

Note: Since pre-treatment ODA disbursement data are not available, 2005 was defined as the first usable year in the series, allowing us to compare mortality trajectories in the periods preceding and following this reference point. Notably, the observed parallel trends remain consistent irrespective of the particular early-year cutoff selected.

We applied two complementary strategies to estimate the DiD effects combined with PSM. First, we employed the standard implementation using the STATA diff command. Second, we followed the stepwise approach recommended in the World Bank DiD handbook, which estimates DiD effects through fixed-effects Poisson panel models and reports results as rate ratios (RR).

WEB TABLE 26 summarizes the DiD results incorporating PSM. After adjusting for observable country/territory characteristics through kernel matching, the initial difference (2002) indicates that countries with higher ODA per capita disbursements exhibited higher overall mortality (ASMR) and child mortality compared with those receiving little or no ODA. By 2019 (the second difference), mortality declined in both groups; however, the reduction was substantially larger among countries with higher ODA support, resulting in a statistically significant DiD estimate.

WEB TABLE 26 presents the estimates from the fixed-effects Poisson panel models. These results corroborate the DiD findings, showing that higher ODA per capita levels were associated with greater reductions in overall and child mortality relative to countries/territories with minimal or no ODA disbursements. The estimated rate [(RR = 0.986; 95% CI: 0.979–0.992); (RR = 0.962; 95% CI: 0.949–0.976)] reflects a modest but statistically meaningful protective effect.

Taken together, these analyses suggest that ODA disbursements contributed to measurable improvements in overall and child mortality rates. The convergence of results from both PSM-adjusted DiD estimates and Poisson panel models strengthens the credibility of the findings presented in the main manuscript and underscores the robustness added through methodological triangulation.

WEB TABLE 25 Propensity Score–Matched Difference-in-Differences Estimates of the impact of intermediate-to-high ODA per capita disbursements on overall and child mortality in low-income countries, 2002–2019.

	**Overall**<br>ASMR	**Child**<br>Under-5 years
Before (2002)		
Control	17.490	136.947
Treated	19.647	160.935
1st Difference (T-C)	2.157***	23.987***
	(0.000)	(0.003)
After (2019)		
Control	10.454	46.103
Treated	12.251	68.425
2nd Difference (T-C)	-1.798***	22.322***
	(0.001)	(0.003)
**Diff-in-Diff**	**-0.359***<br>(0.000)**	**-1.665***<br>(0.005)**

WEB TABLE 26 Rate Ratios from the difference-in-difference fixed effect Poisson models for the association between mortality rates (Overall ASMR and child) with intermediate to high ODA disbursement, in 2002 and 2019, in Low Income countries/territories.

	**ASMR**	**Child**
**ODA per capita (dummies)**		
ODA_dd	4.31342e+12***	3.39503e+33***
	[4480481,4.153e+18]	[3.062e+21,3.764e+45]
ODA Intermediate to high	0.986***	0.962***
	[0.979,0.992]	[0.949,0.976]
**Control Variables**		
Economic downturn	0.942	0.779
	[0.802,1.106]	[0.507,1.197]
GDP per capita	0.879***	0.791
	[0.810,0.955]	[0.570,1.098]
Conflict/wars index	1.298	1.602**
	[0.876,1.924]	[1.004,2.556]
Fertility rate	1.005	0.922
	[0.883,1.144]	[0.656,1.295]
Urban population	0.920	0.844
	[0.823,1.029]	[0.654,1.090]
Primary education	0.888**	0.846
	[0.802,0.983]	[0.604,1.185]
Basic drinking water	1.002	1.133
	[0.895,1.121]	[0.905,1.419]
Health expenditure	0.940	0.752*
	[0.869,1.017]	[0.550,1.029]
Social protection coverage	0.906*	0.953
	[0.816,1.006]	[0.796,1.141]
**Number of observations**	116	116
**Number of countries**	58	58

Source: Author's data analysis for 116 observations – 58 countries/territories in the World, over 2 years (from 2002 and 2019).

Note: GDP=Gross Domestic Product; Data are in Rate Ratio (RR) coefficients (95% CI) unless otherwise specified. The confidence intervals are in parentheses. The symbols '***' , '**' and '*' denote significance at 1%, 5%, and 10% respectively.

Covariate balance before and after propensity score matching

WEB TABLE 27 Covariate balance before and after propensity score matching in low-income countries (LIC).

Covariates	Before matching			After matching		
	Control	Treated	Diff. (p-value)[1]	Control	Treated	Diff. (p-value)[1]
Economic growth (%)	3.91	5.44	0.041	4.72	4.86	0.812
GDP per capita PPP	2,985	4,342	0.002	3,694	3,812	0.761
Conflict index	0.13	0.07	0.036	0.1	0.09	0.658
Fertility rate	5.12	4.23	0.018	4.61	4.56	0.903
Urban population (%)	29.74	41.21	0.000	35.52	36.13	0.824
Primary education (%)	42.31	57.46	0.001	49.02	50.21	0.781
Basic drinking water (%)	53.02	69.37	0.000	61.04	62.73	0.733
Health expenditure per capita	82.14	141.88	0.000	112.51	116.43	0.645
Social protection (%)	15.03	25.41	0.003	20.02	20.96	0.704

Note: [1] Differences between treatment and control groups were assessed using two-sample t-tests of mean equality. The null hypothesis (H_0) is that the mean value of each covariate is equal in the treated and control groups (i.e., no systematic differences between groups). Before matching, statistically significant p-values indicate imbalance between groups. After propensity score matching, the differences become statistically non-significant and the covariate means become very similar across groups, suggesting that the matching procedure substantially improved covariate balance.

PART III – FORECASTING ANALYSIS

5. PURPOSE OF THE FORECASTING AND ITS APPLICATIONS

The main goal is to simulate the dismantling of policies currently being implemented within ODA funding for 59 LICs and LMCs that are particularly vulnerable to the consequences of this disruption. Our focus is on evaluating alternative scenarios for the redistribution of ODA funds that could help prevent a significant number of avoidable deaths.

6. DESCRIPTION OF THE FORECASTING METHODOLOGY

6.1. Scenarios

The current defunding scenario is defined as the ODA that would result from an ODA defunding from 2025 to 2030. For computing this, we use real ODA data until 2023, as it was the last year with ODA Creditor Report System (CRS) flow-based consolidated data. We then assumed a defunding rate of 6.33% to compute 2024 ODA, and later 15.8% from 2024 to obtain the 2025 ODA. For the following years we applied an annual decrease of 11.1% until 2030.

For computing the different funding scenarios, we assumed certain adjusting progression years to fully reach the total entering fund by 2030. For this, we applied a bounded exponential growth function with diminishing returns that represents a high entering at the beginning with diminishing strength until reaching the total set (the bound) in 2030.

This is represented by the following equation:

$$y(t) = A * (1 - e^{(b*t)})$$

Being,

- A is the fund sum we want to reach by 2030
- b the diminishing return (which was set to -1)
- t the time number

Then, conceptually, the current year ODA is the sum of the ODA of the past year plus the proportional share of new funds (i.e., proportional in terms of the past year ODA proportion over the overall ODA). However, when a country is in quintile 5, its mortality cannot go down, we set the rule that we will not add more funds to a country when it reaches quintile 5 in the previous year, but we leave its ODA as in the previous year. Because of this, when the total ODA of a year is greater than the one of 2023 (i.e., the total funds entering in the year are enough to cover what the total defunded funds are) we take this into consideration.

Thus, the country's quintile ODA per capita computation for each scenario was done in the following way, for each different funding amount:

1. We computed the 2023 proportion of ODA that each country represented over the 2023 total ODA (we use this proportion as it was the last year ODA distribution available in the CRS).
   I. We multiplied this proportion by the total ODA available in the current year after having discounted the total ODA defunding of the year. This gives us the ODA share of the country after the defund.
2. We set the rule that if the total funding of the year is greater than the total defunding (i.e., the total ODA of the year is greater than the ODA in 2023), no country will continue receiving ODA once it reaches the quintile 5 (i.e., despite it does not increase its ODA, it maintains the same level of the previous year).
3. Then:
   I. *If in the current year the total funding is lower than the total defunding,* the country´s new ODA is set as the sum of the ODA share after the defund, plus its share (as computed in step 1) of new entering funds.
   II. *If in the current year the total funding is greater than the total defunding,* we computed the previous year's country´s ODA proportion, only considering the countries that were not on ODA quintile 5 in the previous year.
      i. We multiplied this proportion by the total ODA entering funds (minus the funds needed to maintain the countries in previous year quintile 5), to compute the country´s assigned share of entering funds.
      ii. The new country ODA is the sum of the ODA after having discounted its defunding (and for the countries with previous year quintile 5, its total ODA of the previous year) plus its assigned share of new entering funds.
4. The ODA per capita is computed by dividing the resulting ODA by the total population.
5. The ODA per capita is assigned to the corresponding quintile.

We can also express the computing procedure as*:*

Being:

- *i = country*
- *t = year*
- *p t = proportion defunding with respect to ODA 2023 (e.g., if ODA 2023 = 1000 and p 2024 = 0.4, then ODA 2024 = 600)*
- *D t = ODA 2023 – ODA 2023 * (1- p t)*
- *F t = total entering funds in year t*
- *ODA t = total ODA in year t*
- *q t = ODA per capita quintile in year t*

If $ODA_t \leq ODA_{2023}$:

$ODA_{it} = ODAb_{it} + R_{it}$

- $ODAb_{it} = Prop_i * ODAb_t$
  - $Prop_i = ODA_i\ 2023 / ODA\ 2023$
  - $ODAb_t = ODA\ 2023 - D_t = ODA\ 2023 * (1 - p_t)$
- $R_{it} = Prop_i * F_t$

If $ODA_t > ODA_{2023}$ **(we appy the "quintile 5" rule)**:

$ODA_{it} = ODAb_{it} + ODALost_{it} + R_{it}$

- If $q_{t-1} < 5$:
  - $ODAb_{it} = Prop_i * (ODA_{2023} - D_t) = Prop_i * (ODA_{2023} * (1 - p_t))$
  - $ODALost_{it} = 0$
  - $R_{it} = Prop'_i * F'_t = (ODA_{i\,t-1} / \sum ODA'_{it-1}) * (F_t - \sum ODALost_i)$
    - $ODA'_{it-1} = ODA_{it-1}$ for i´s for which $q_{t-1} < 5$
- If $q_{t-1} = 5$:
  - $ODAb_{it} = Prop_i * (ODA_{2023} - D_t) = Prop_i * (ODA_{2023} * (1 - p_t))$
  - $ODALost_{it} = ODA_{it-1} - ODAb_{it}$
  - $R_{it} = Prop''_i * F'_t = (0 * (F_t - \sum ODALost_i) = 0$

6.2. Governance

Accounting for governance is essential when estimating the health impact of ODA on mortality because governance fundamentally shapes whether funds are converted into effective, sustained services rather than being lost to inefficiency, instability, uncertainty, poor institutional enforcement or corruption, among other causes. Strong governance improves planning, procurement, workforce management, monitoring, and cross-sector coordination, all of which increases the fidelity and reach of health interventions and amplifies mortality reductions; weak governance, by contrast, attenuates expected benefits.[26–28] Ignoring this heterogeneity risks producing misleading average effects that overstate impact in poorly governed settings and understate it where institutions function well, undermining both causal inference and policy relevance. Because of this the forecasting analysis is conducted taking governance conditions of countries into consideration.

The forecasting has been implemented considering the ODA (also covariates and fixed effects) effects based on the Rate Ratios estimated in two governance strata variable regressions (estimated with analogous design and specification to the main retrospective estimations).

This has been conducted as follows:

1. Using World Bank Worldwide Governance Indicators we generated a governance related implementation feasibility proxy variable. [29]
2. This data includes 6 governance related variables (i.e., Voice and Accountability, Political Stability and Absence of Violence/Terrorism, Government Effectiveness, Regulatory Quality, Rule of Law, and Control of Corruption). Each of these can be expressed as a 0-100 ratio where countries with better governance conditions have larger ratios. We took the simple average of the six dimensions for each country/year in the sample. Then we dichotomized the ratios given by the median of the sample (i.e., 59 countries from 2002 to 2021).
3. With the country/year dyads categorized in two groups (i.e., worst-off and better-off governance conditions) we generated two stratified regression analyses with the same approach as the main regression analysis (with the only difference that we conducted unconditional fixed effects Poisson instead of the conditional option so to be able to extract the country fixed effects needed for the forecasting analyses).

4. We used the ODA effects (and the covariates and fixed effects) given by the Rate Ratios (and its associated 95% Confidence Intervals) of these two stratified regressions to conduct forecasting analyses.

This made that the forecasting effects generated with a distinct effect given the country's government condition.

A graph of the evolution of the Governance ratio can be seen in Figure 6. The results of the two governance strata regression are shown in Table 28.

WEB FIGURE 6 Mean Governance Ratio by year (59 countries, from 2002 to 2021)

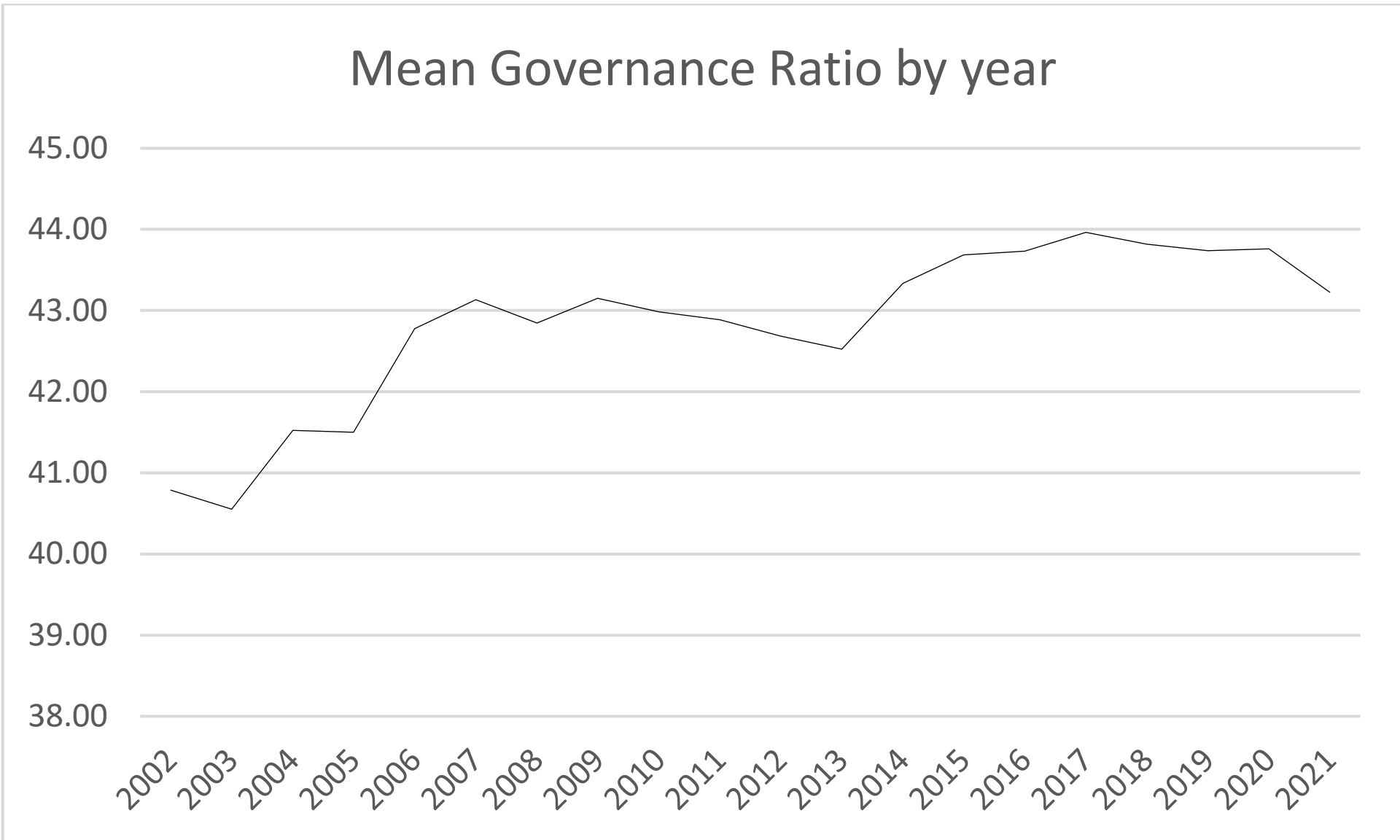

WEB TABLE 28 Adjusted rate ratios from multivariable fixed-effects Poisson models with robust standard errors for the association between mortality rates and annual ODA per capita in both governance strata from 2002 to 2021.

	Overall (ASMR), Governance strata Lower	Overall (ASMR), Governance strata Upper	Under-five, Governance strata Lower	Under-five, Governance strata Upper
Baseline (mean 6.38, 0-12.34)	1	1	1	1
	[1.000,1.000]	[1.000,1.000]	[1.000,1.000]	[1.000,1.000]
Low (mean 18.68, 12.35-24.40)	0.955***	0.907***	0.946**	0.803***
	[0.929,0.983]	[0.862,0.955]	[0.899,0.996]	[0.745,0.865]
Lower Intermediate (mean 30.21, 24.41-35.97)	0.850***	0.873***	0.772***	0.694***
	[0.809,0.894]	[0.820,0.929]	[0.705,0.845]	[0.621,0.775]
Upper Intermediate (mean 42.81, 35.98-52.12)	0.810***	0.787***	0.771***	0.599***
	[0.764,0.859]	[0.732,0.845]	[0.699,0.851]	[0.536,0.669]

High (mean 83.03, 52.13 or more)	0.817***	0.765***	0.837**	0.550***
	[0.754,0.884]	[0.708,0.828]	[0.718,0.977]	[0.482,0.629]
Economic downturn	1.011	0.982	0.967	0.899*
	[0.969,1.056]	[0.941,1.025]	[0.898,1.040]	[0.795,1.018]
GDP per capita	0.922***	0.975**	0.964	0.906***
	[0.897,0.948]	[0.954,0.995]	[0.912,1.019]	[0.860,0.954]
Conflict/wars index	1.190***	1.156*	1.478***	0.777
	[1.043,1.357]	[0.983,1.361]	[1.145,1.909]	[0.530,1.139]
Fertility rate	0.988	1.064***	0.951	1.031
	[0.951,1.026]	[1.024,1.106]	[0.873,1.035]	[0.936,1.135]
Urban population	0.955***	0.793***	0.839***	0.688***
	[0.924,0.987]	[0.708,0.889]	[0.770,0.913]	[0.596,0.795]
Primary education	0.920***	0.989	0.793***	0.957
	[0.892,0.948]	[0.934,1.048]	[0.736,0.855]	[0.847,1.082]
Basic drinking water	0.948***	0.960**	0.917**	0.768***
	[0.912,0.986]	[0.925,0.997]	[0.854,0.985]	[0.694,0.849]
Health expenditure	0.981	0.919***	0.908**	0.808***
	[0.953,1.010]	[0.896,0.943]	[0.844,0.978]	[0.750,0.869]
Social protection coverage	0.893***	1.019	0.854***	1.037
	[0.865,0.923]	[0.992,1.047]	[0.807,0.904]	[0.984,1.091]
y2009	1.010	1.049***	1.028	1.162***
	[0.980,1.040]	[1.031,1.067]	[0.971,1.088]	[1.089,1.241]
y2015	0.961**	0.996	0.903***	0.935**
	[0.929,0.995]	[0.967,1.026]	[0.865,0.943]	[0.880,0.994]
y2020	1.090***	1.035	0.823***	0.808***
	[1.046,1.137]	[0.989,1.082]	[0.736,0.920]	[0.706,0.925]
y2021	1.182***	1.125***	0.748***	0.694***
	[1.149,1.216]	[1.085,1.167]	[0.672,0.833]	[0.635,0.759]
N	588	589	588	589

* $p < 0.10$, ** $p < 0.05$, *** $p < 0.01$

7. DATA SOURCE AND INPUTS

The panel data used in this study combined aggregated demographic, socioeconomic, health, and ODA info from several sources (all sources are publicly available and listed in the WEB TABLE 1). In the absence of reference values from the literature, we categorized all ODA information using the same quintile levels applied in the retrospective analysis. This classification captures the context in which ODA operates, enabling a more accurate interpretation of funding patterns. As input for the modelling and forecasting phases, all control variables applied in the retrospective analysis were included in the models and the governance variable.

8. POISSON MULTIVARIABLE REGRESSION MODELS WITH ROBUST STANDARD ERRORS AND FIXED-EFFECTS

To better capture the effects of interest in our study, we employed a Generalized Linear Model (GLM) with a Poisson distribution, incorporating robust standard errors to account for potential misspecification of the variance structure due to the panel data design.[5,6,8,10,11] Then, let Y_{it} be the observed count of deaths (overall or under-5) and assuming $Y_{it} \sim Poisson(\mu_{it})$, the linear predictor of the model, considering the population offset and country fixed effect, is

$$\log(\mu_{it}) = \log(pop_{it}) + \beta 0 + \boldsymbol{X_{it}\beta} + \alpha_i + \theta_t, (1)$$

where μ_{it} is the expected number of the event of interest, pop_{it} and $\boldsymbol{X_{it}}$ are the population of interest size and the vector of covariates in time t for the observation i, respectively, β_0 is the intercept, $\boldsymbol{\beta}$ is the corresponding vector of regression coefficients, α_i denotes the unit-specific fixed effect (assumed time-invariant), and θ_t is the time-trend.

9. CALIBRATION, TIME TREND, AND MONTE CARLO SIMULATION

9.1. Calibration

In the forecasting analysis, all parameter estimates and their respective standard errors were derived from the retrospective dataset and the model. The intercept calibration procedure was only necessary for the children's mortality models. We calibrated the intercept, β_0 parameter of the regression model. The calibration process was based on data from 2015 to 2021. We applied Monte Carlo simulations with the current defunding scenario, varying the value of β_0 to find the one with lower Root Mean Squared Error (RMSE).

To sample the calibration parameters, we utilized a Latin Hypercube Sample (LHS) method,[30] based on the *tgp* package in R.[31] First, we identified convergence zones for the parameters with small LHS samples and a lower number of simulations. After identifying these zones, we sampled 100 parameters and simulated each one a thousand times to compare their mean results with the mortality rates of 2015 to 2021. We made this for each level of governance. The RMSE results for each sample can be assessed in the web figures below.

WEB FIGURE 7 RMSE results of all LHS parameters for the child mortality rate model governance level one.

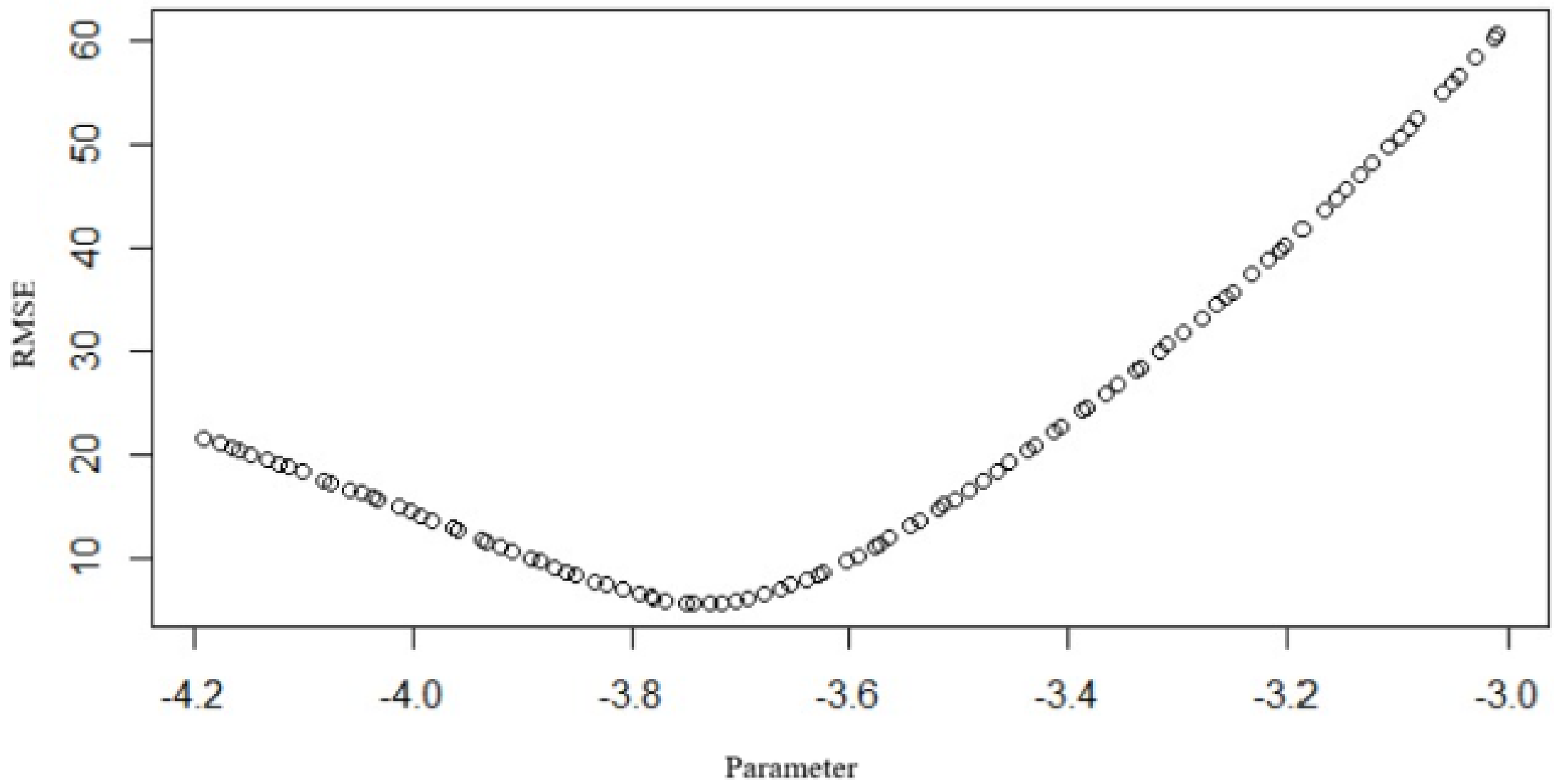

WEB FIGURE 8 RMSE results of 100 LHS parameters for the child mortality rate model governance level two.

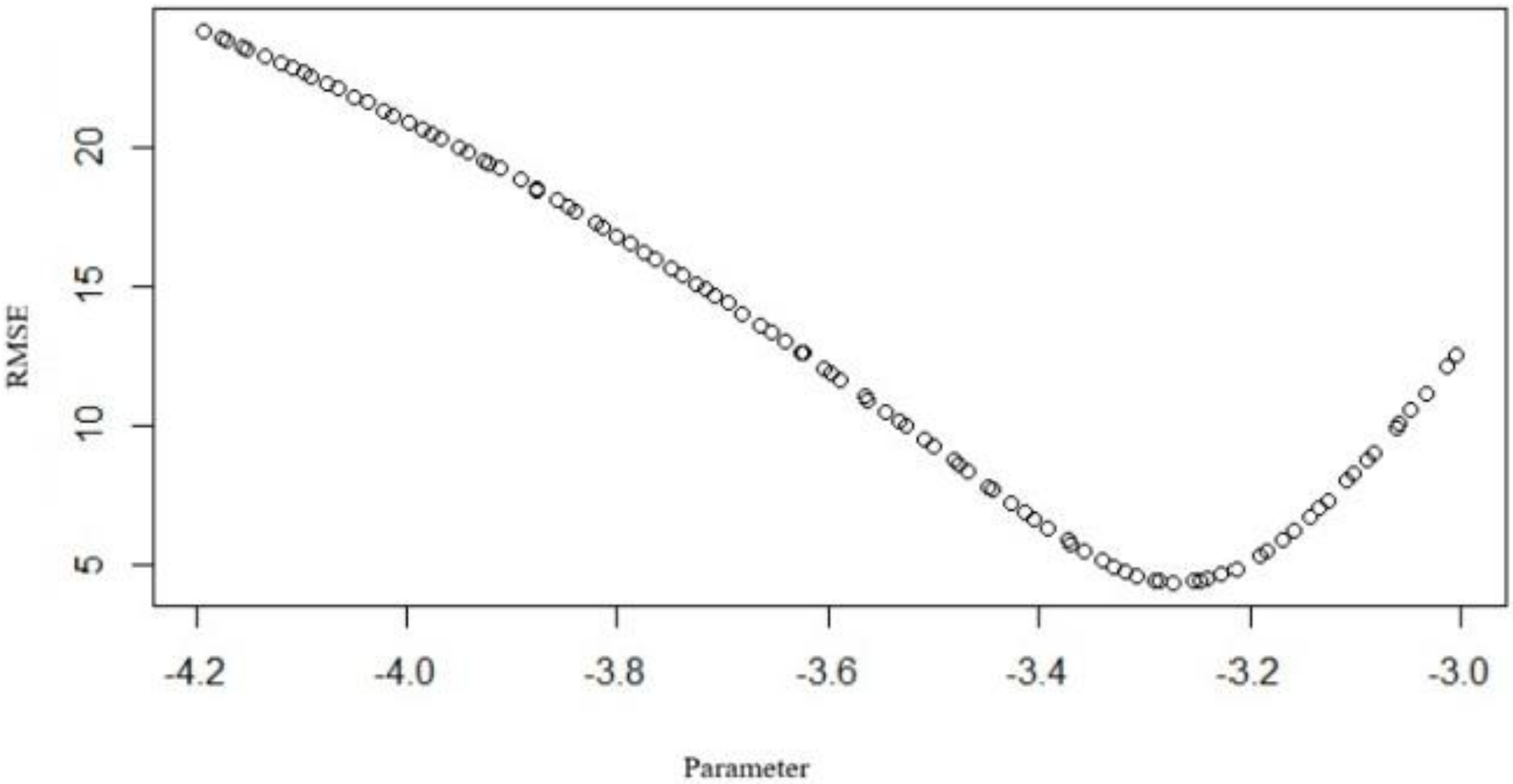

The WEB TABLE 29 presents the top six parameters with the lowest RMSE values. The calibrated values of β_0 for the ASMR and child mortality rate models were determined to be -5.755 and -4.382, respectively.

WEB TABLE 29 Final estimates after model refinement and calibration based on minimum RMSE.

Child mortality rate governance level one		Child mortality rate governance level two	
**Parameter**	**RMSE**	**Parameter**	**RMSE**
-3.728	5.755	-3.271	4.382
-3.742	5.766	-3.252	4.415
-3.749	5.809	-3.284	4.435
-3.717	5.831	-3.245	4.459
-3.703	6.020	-3.289	4.470
-3.767	6.047	-3.238	4.520

9.2. Time trend

To estimate the underlying time trend in mortality rates, we follow the same strategy as Cavalcanti et al.. [32] We fitted a linear regression model using the logarithm of the mortality rate per 1,000 inhabitants as the dependent variable and the calendar year as the independent variable. We made this for each set of countries at governance level one and two.

We excluded the years 2020 and 2021 due to the direct impact of the COVID-19 pandemic on death counts, which could distort the long-term trend. After this, we applied a logarithmic transformation to the mortality rate to stabilize the variance, better capture proportional (multiplicative) changes, and improve the model's fit within a standard Gaussian linear regression framework using the mortality rate per 1,000 inhabitants for the ASMR model and the mortality rate per 1,000 live births in the child mortality rate model, as the dependent variable and the calendar year as the independent variable.

In this framework, the estimated coefficient associated with the year variable can be interpreted as the average annual rate of change in the log mortality rate.

The estimated coefficient associated with the year variable is -0.0219, with a standard error of 0.001 for governance level one, and -0.0168, with a standard error of 0.0007 for governance level two, indicating a statistically significant decreasing trend in the log mortality rate over time. A residual analysis was conducted to assess the adequacy of the linear regression assumptions. In addition, the residuals appear to be symmetrically distributed around zero, supporting the appropriateness of the linear regression model.

WEB FIGURE 9 Q-Q plot of the residuals from the linear regression model fitted to the ASMR per 1,000 inhabitants for the governance level one (left) and two (right).

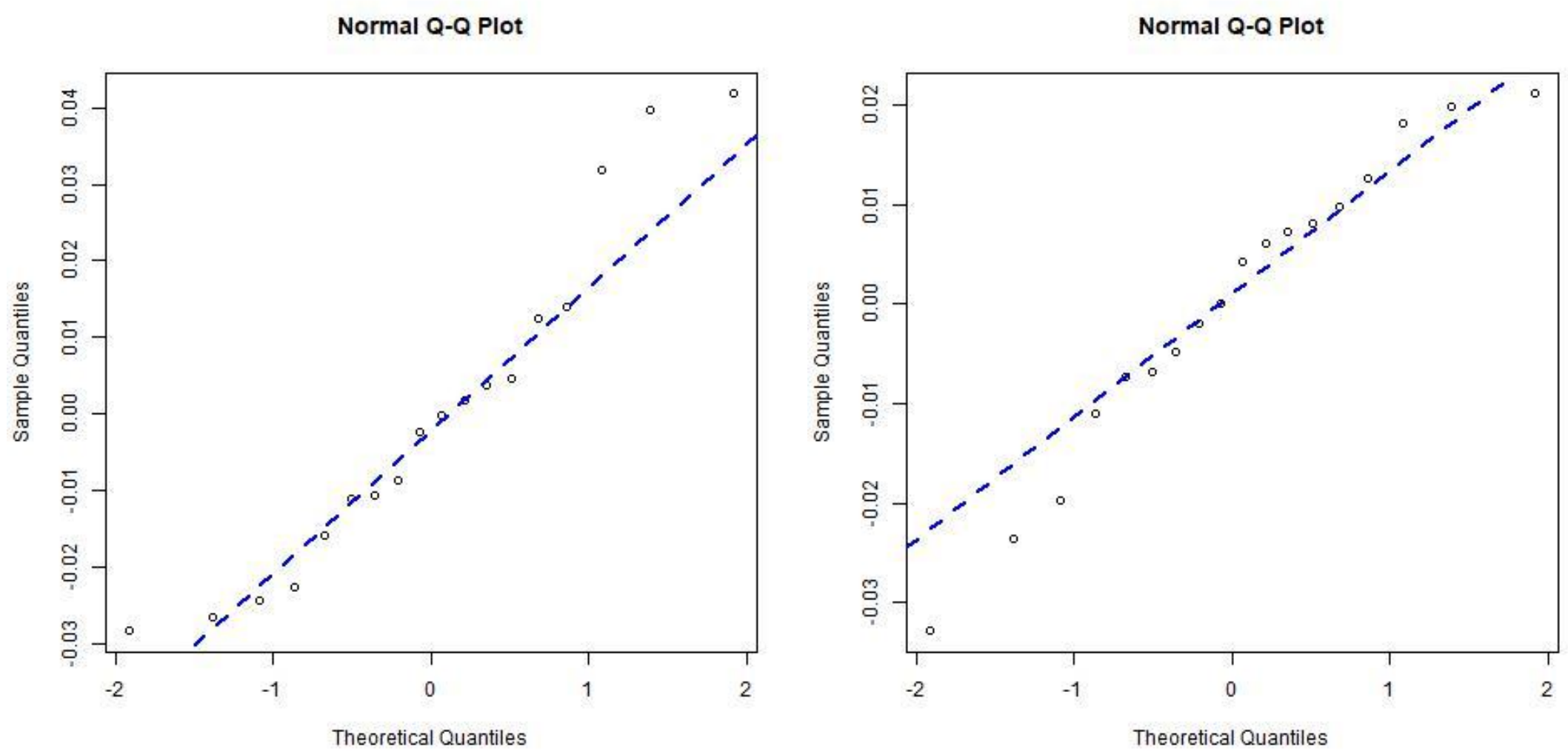

The same procedure was applied to the child mortality rate, resulting in an estimated coefficient of -0.043with a standard error of 0.0011, for governance level one, and -0.0383 with a standard error of 0.0013 for governance level two. As in the previous model, the regression assumptions were assessed and validated.

WEB FIGURE 10 Q-Q plot of the residuals from the linear regression model fitted to child mortality rate per 1,000 live births for the governance level one (left) and two (right).

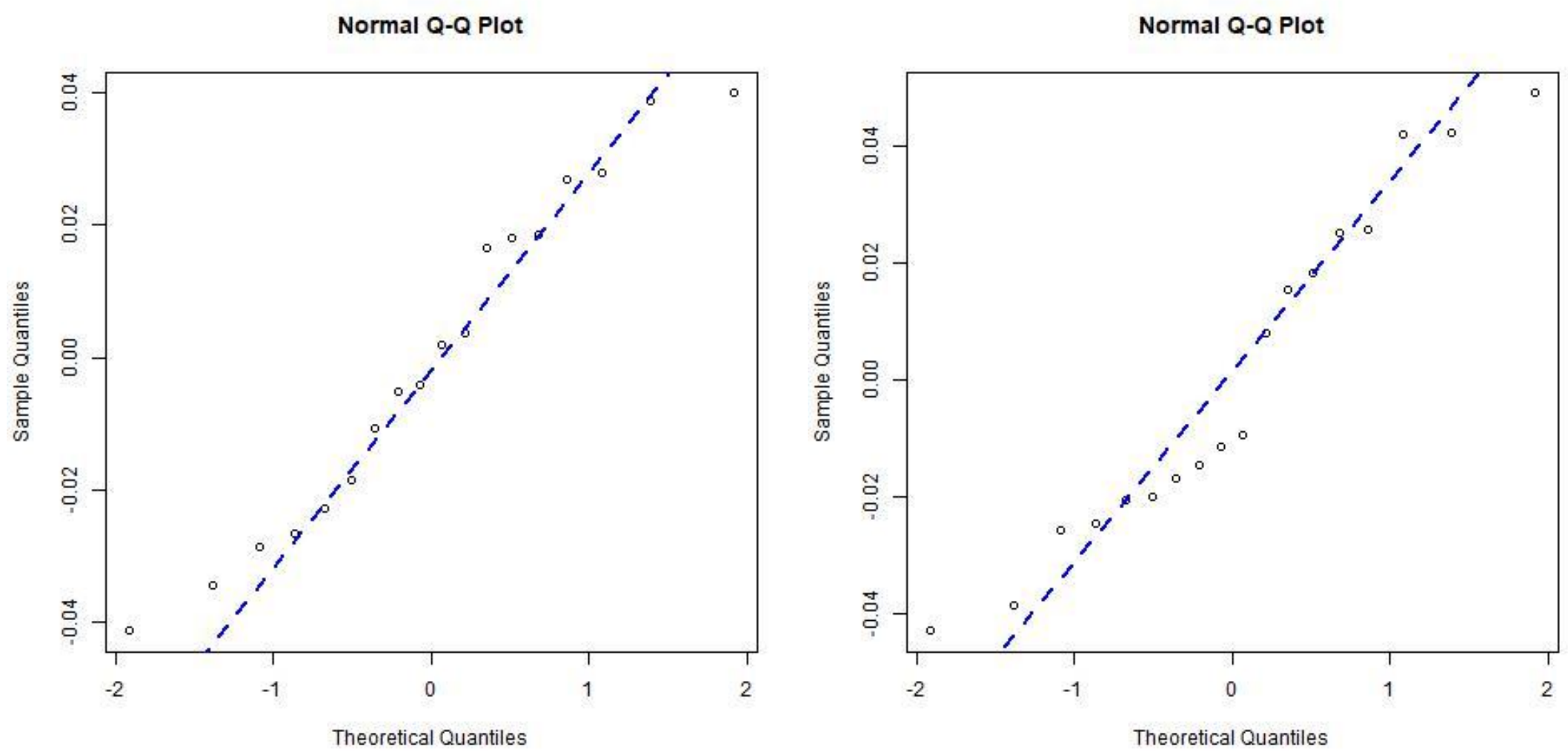

9.3. Monte Carlo Simulation

We employed 1,000 Monte Carlo replications. This number was selected after confirming the stability of simulation outputs, which consistently converged between 300 and 500 replications.[33] All computational procedures were carried out using the free statistical software R

(version 4.2.1) in a Linux environment. For each MC replication and policy scenario, the following prediction procedure was applied:

1. Based on the governance stratification regression estimates, along with the time trend and their respective confidence intervals, we randomly selected a value for each parameter based on the normal distribution, with the mean and standard error being the point estimate and standard error from the regression analysis.
2. Using the synthetic cohort, we calculated the expected mean number of deaths for each observation, defined at the country-year level.
3. A count outcome was then generated by drawing from a Poisson distribution, with the previously estimated mean used as the distribution's parameter.
4. Steps 1 to 3 were repeated until the desired number of MC replications was reached.

It is important to highlight that Step 1 of the algorithm plays a key role in introducing randomness and uncertainty into the study, which is a crucial aspect in microsimulation approaches. The final predictions and corresponding uncertainty intervals for each outcome variable were derived from the simulation results: the mean of the 1,000 replications was used as the point estimate, while the 2.5th and 97.5th percentiles were used to construct 95% confidence intervals.

10.VALIDATION

To validate our approach, we implement two different strategies based on the specificities of each external data source. For the ASMR model, we predict expected values based on the United Nations World Population Prospects 2024 (WPP2024) association with our data. For the child mortality rate, we compare the existing values of the UNICEF data source. A comparison between the simulated results and the values obtained from external sources can be assessed in the WEB TABLE 30.

WEB TABLE 30 Comparison between Simulated and external sources in 2022 and 2023 across 59 Countries.

	ASMR			Child Mortality Rate	
**Year**	**Model**	**WPP2024**	**WPP-Prediction**	**Model**	**Unicef**
2022	11.2 (9.89 - 12.8)	7,16	11.0 (10.6 – 11.4)	53.2 (45.6 - 61.2)	48.53
2023	11.0 (9.66 - 12.5)	7.01	10.9 (10.5 – 11.2)	50.7 (43.5 - 58.6)	46.92

For the ASMR model, we employ an alternative validation approach. We utilize the Crude Death Rate (deaths per 1,000 population) provided by the WPP2024 as a source of external data for validation (https://population.un.org/wpp). There are historical discrepancies between this data and the data used in this study, which can be attributed to differences in how each is calculated. Nevertheless, we leverage the historical correlation to predict values for 2022 and 2023. We achieve this by employing a linear regression model, where the mortality rates used in this study serve as the dependent variable, while the WPP2024 mortality rates and a time trend for the observed period act as independent variables. We applied the model using data from 2002 to 2021 and used it to forecast the values for 2022 and 2023, characterizing an external validation. The results can be appreciated in WEB FIGURE 11.

WEB FIGURE 11 Simulated Mortality Rates with Uncertainty Intervals and Projected Values based on WPP2024 (2022–2023).

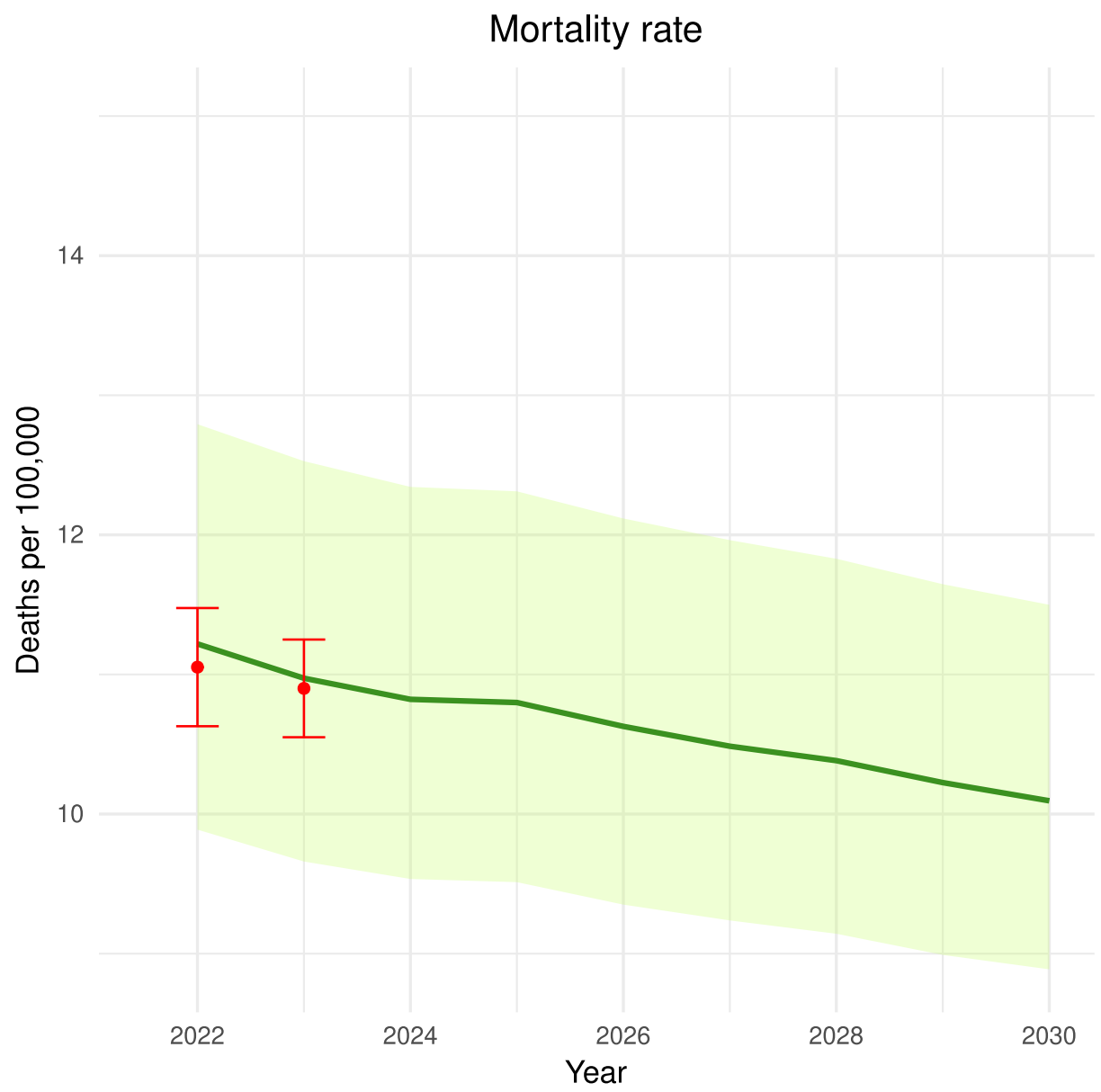

For the child mortality rate model, we compared the child mortality rates simulated by our model with the official child mortality rate estimates available from UNICEF (source https://data.unicef.org/topic/childsurvival/under-five-mortality/#data) for the 59 countries included in the study. The comparison focused on the years 2022 and 2023. The values observed from UNICEF fall within the uncertainty intervals of our model and with similar trend, indicating strong agreement between the simulated and empirical data.

WEB FIGURE 12 Simulated Child Mortality Rates with Uncertainty Intervals and Observed Values from UNICEF (2022–2023).

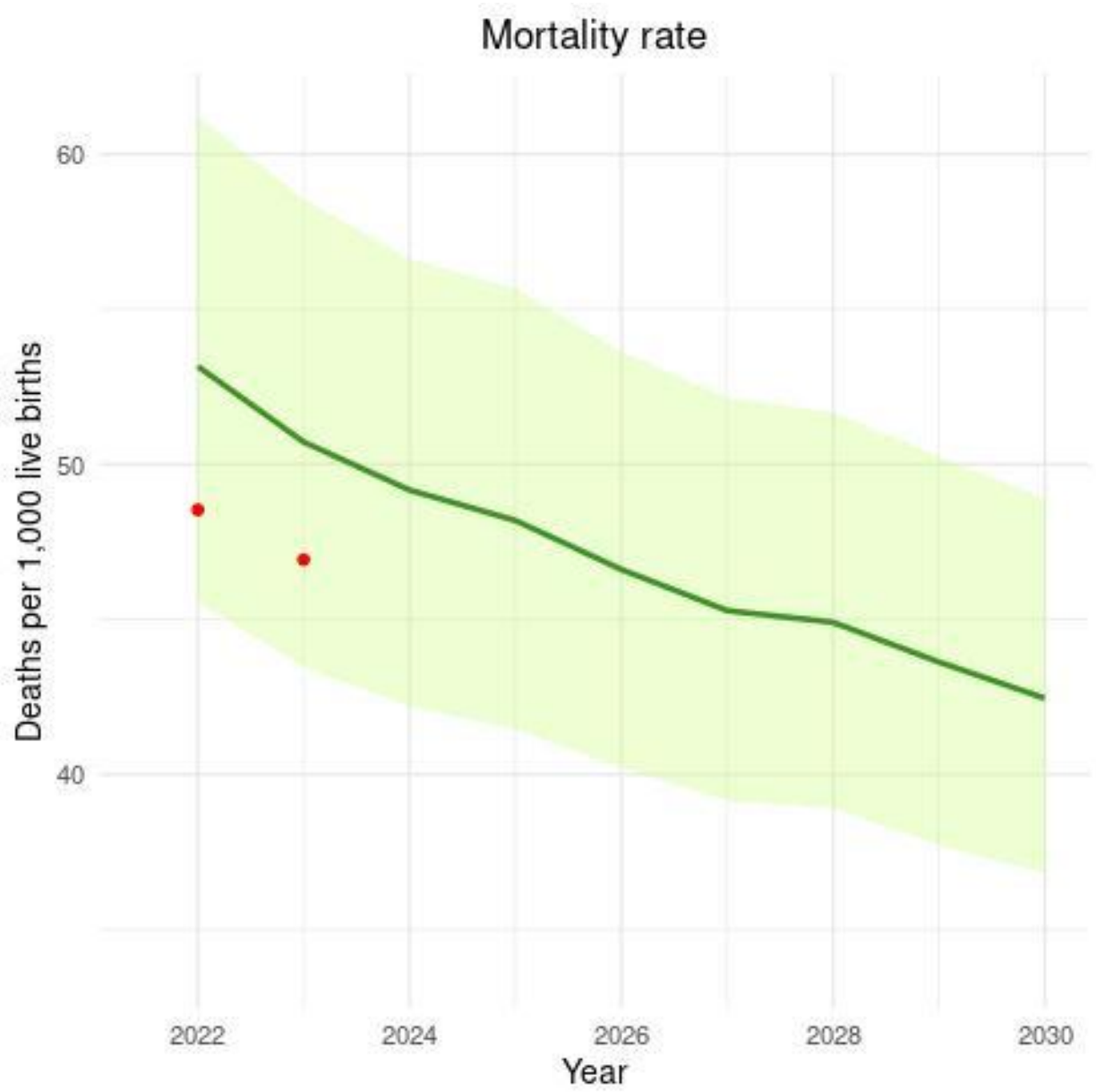

11.GRAPH OF AVERTED DEATHS BY SCENARIO

WEB FIGURE 13 Averted Deaths by scenario

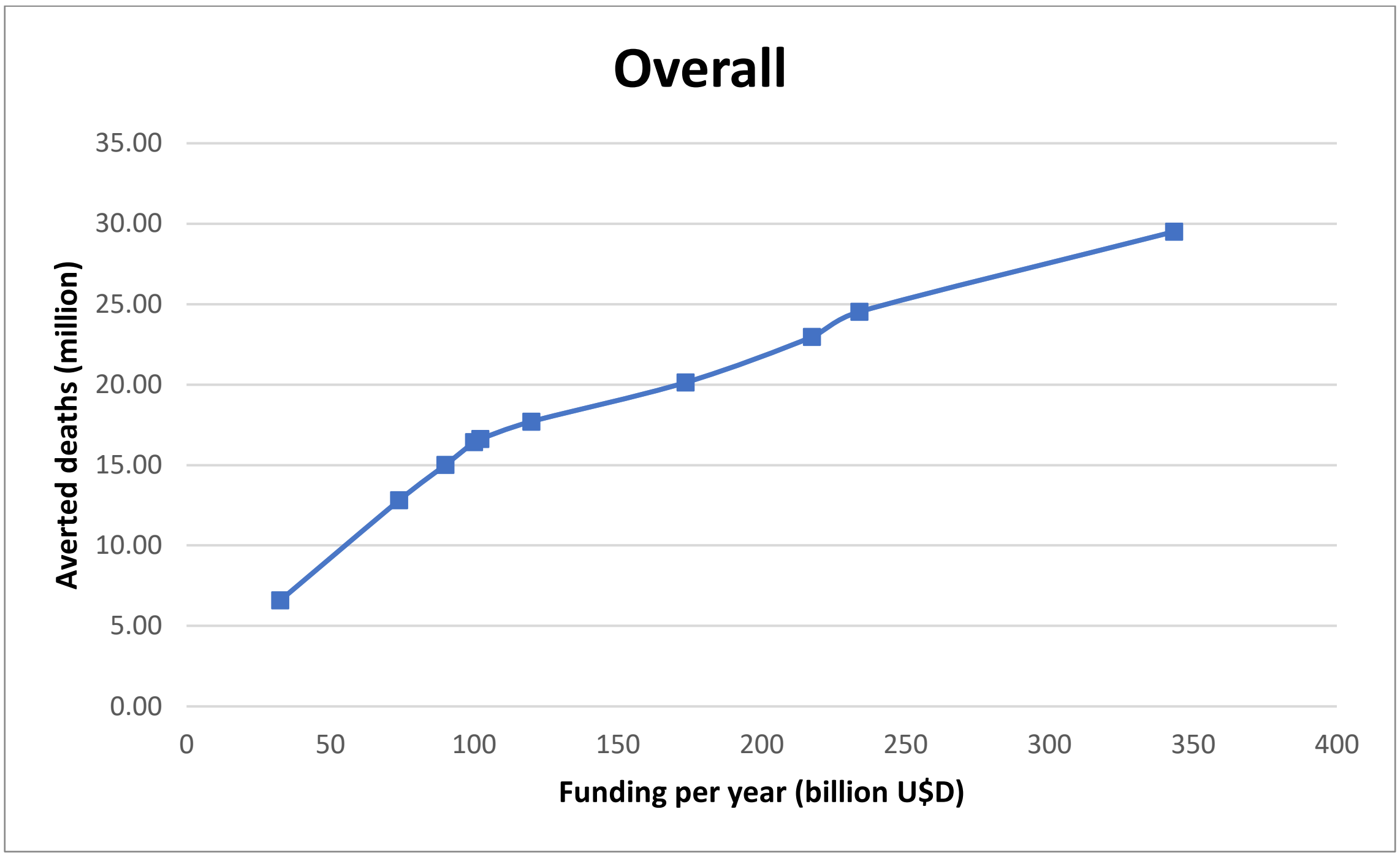

12.SUB-GROUP ANALYSIS FOR BY GOVERNANCE STRATA

WEB TABLE 31 Overall main results by governance strata (averted deaths, by year and total), 2026-2030

	Worst-off Governance			Better-off Governance		
**BAU Scenario**	**Av. Deaths**	**ui_low**	**ui_high**	**Av. Deaths**	**ui_low**	**ui_high**
**2026**	737,283	491,624	1,004,323	304,762	140,643	484,758
**2027**	921,599	691,473	1,162,311	331,939	180,718	495,212
**2028**	1,198,319	925,568	1,473,420	402,615	239,707	588,894
**2029**	1,283,815	1,003,528	1,564,555	440,042	247,878	637,113
**2030**	1,327,329	1,036,271	1,626,249	610,719	402,531	816,134
**Total**	**5,468,345**	4,210,627	6,784,850	**2,090,077**	1,251,522	2,983,211
**Crypto Tax (3% top 10,000 holders)**	**Av. Deaths**	**ui_low**	**ui_high**	**Av. Deaths**	**ui_low**	**ui_high**
**2026**	737,282	491,624	1,004,323	304,765	140,643	484,758
**2027**	921,600	691,473	1,162,311	331,937	180,718	495,212
**2028**	1,198,326	925,568	1,473,420	402,611	239,707	588,894
**2029**	914,667	645,634	1,173,228	382,761	195,329	561,220
**2030**	896,397	624,456	1,167,025	501,639	357,257	653,310
**Total**	**4,668,271**	3,609,354	5,722,966	**1,923,713**	1,184,496	2,718,515
**Interest Earnings Debt Tax (100% of interest payments by LMICs to official creditors)**	**Av. Deaths**	**ui_low**	**ui_high**	**Av. Deaths**	**ui_low**	**ui_high**
**2026**	1,085,953	628,522	1,593,624	567,615	307,577	837,838
**2027**	1,826,527	1,164,792	2,449,278	734,542	393,736	1,081,068

**2028**	2,083,608	1,376,657	2,766,799	798,507	459,859	1,150,540
**2029**	2,149,854	1,450,062	2,825,606	829,341	491,935	1,150,831
**2030**	1,753,305	1,139,752	2,326,600	993,503	672,327	1,318,996
**Total**	**8,899,248**	5,963,973	11,718,388	**3,923,508**	2,343,769	5,457,585
**Ultra-high-net-worth individuals (1% billionaires)**	**Av. Deaths**	**ui_low**	**ui_high**	**Av. Deaths**	**ui_low**	**ui_high**
**2026**	1,209,187	645,517	1,762,167	686,049	415,346	981,468
**2027**	2,020,399	1,249,745	2,750,941	1,002,855	656,647	1,359,567
**2028**	2,311,213	1,510,677	3,089,712	1,069,939	695,593	1,449,677
**2029**	2,335,407	1,556,992	3,118,050	1,096,249	737,913	1,457,828
**2030**	2,356,032	1,590,894	3,117,526	1,000,845	658,116	1,334,788
**Total**	**10,232,238**	6,708,019	13,683,715	**4,855,938**	3,213,365	6,529,979
**Crypto Tax IMF2023**	**Av. Deaths**	**ui_low**	**ui_high**	**Av. Deaths**	**ui_low**	**ui_high**
**2026**	1,669,085	1,122,472	2,192,180	689,987	408,604	985,025
**2027**	2,230,756	1,491,230	2,954,481	1,010,578	630,486	1,384,694
**2028**	2,479,066	1,709,126	3,236,647	1,069,939	695,593	1,449,677
**2029**	2,536,728	1,771,687	3,279,974	1,096,249	737,913	1,457,828
**2030**	2,392,340	1,632,600	3,133,412	1,255,967	893,297	1,628,368
**Total**	**11,307,974**	7,769,414	14,656,728	**5,122,720**	3,426,748	6,843,673
**Carbon Tax**	**Av. Deaths**	**ui_low**	**ui_high**	**Av. Deaths**	**ui_low**	**ui_high**
**2026**	1,669,085	1,122,472	2,192,180	714,186	380,918	1,054,182
**2027**	2,230,756	1,491,230	2,954,481	1,010,578	630,486	1,384,694
**2028**	2,479,066	1,709,126	3,236,647	1,069,939	695,593	1,449,677
**2029**	2,536,728	1,771,687	3,279,974	1,096,249	737,913	1,457,828
**2030**	2,552,979	1,827,554	3,285,297	1,255,967	893,297	1,628,368
**Total**	**11,468,613**	8,009,182	14,883,966	**5,146,919**	3,393,814	6,924,712
**Interest Earnings Debt Tax (50% of interest payments by LMICs to official creditors + 25% of interest payments by LMICs to private lenders)**	**Av. Deaths**	**ui_low**	**ui_high**	**Av. Deaths**	**ui_low**	**ui_high**
**2026**	1,662,267	1,039,099	2,256,114	714,186	380,918	1,054,182
**2027**	2,405,487	1,649,567	3,197,969	1,041,881	657,993	1,416,351
**2028**	2,690,636	1,851,918	3,525,973	1,165,258	798,083	1,547,622
**2029**	2,739,834	1,914,862	3,558,824	1,189,973	829,181	1,551,281
**2030**	2,751,664	1,953,047	3,550,303	1,348,132	995,223	1,722,597
**Total**	**12,249,887**	8,501,131	15,942,959	**5,459,431**	3,705,042	7,294,564
**Corporate Tax**	**Av. Deaths**	**ui_low**	**ui_high**	**Av. Deaths**	**ui_low**	**ui_high**
**2026**	2,075,465	1,355,355	2,809,729	994,900	617,288	1,378,205
**2027**	2,499,506	1,318,327	3,619,461	1,292,432	861,230	1,737,982
**2028**	2,735,841	1,472,327	3,911,193	1,588,567	1,088,548	2,127,166
**2029**	2,785,386	1,528,765	3,962,883	1,606,223	1,102,275	2,138,575
**2030**	2,796,229	1,579,523	3,917,753	1,757,448	1,256,977	2,292,994
**Total**	**12,892,428**	7,367,812	18,176,107	**7,239,571**	5,017,354	9,527,260
**Ultra-high-net-worth individuals (2% billionaires)**	**Av. Deaths**	**ui_low**	**ui_high**	**Av. Deaths**	**ui_low**	**ui_high**
**2026**	2,254,075	1,506,632	3,053,599	1,026,736	668,140	1,384,893
**2027**	2,493,235	1,244,737	3,645,280	1,292,432	861,230	1,737,982

**2028**	2,827,114	1,526,651	4,032,676	1,588,567	1,088,548	2,127,166
**2029**	2,877,226	1,601,723	4,051,961	2,793,339	2,004,634	3,626,642
**2030**	2,886,072	1,647,256	4,030,218	2,924,775	2,124,413	3,769,013
**Total**	**13,337,722**	7,700,154	18,711,170	**9,625,849**	6,991,032	12,349,922
**Tobin Tax**	**Av. Deaths**	**ui_low**	**ui_high**	**Av. Deaths**	**ui_low**	**ui_high**
**2026**	2,297,163	1,553,755	3,086,465	1,093,475	732,041	1,475,487
**2027**	2,493,235	1,244,737	3,645,280	1,537,992	1,026,995	2,071,888
**2028**	2,827,114	1,526,651	4,032,676	2,795,807	1,948,833	3,704,534
**2029**	2,877,226	1,601,723	4,051,961	2,793,339	2,004,634	3,626,642
**2030**	2,886,072	1,647,256	4,030,218	2,924,775	2,124,413	3,769,013
**Total**	**13,380,811**	7,730,432	18,750,224	**11,145,388**	8,047,988	14,353,532
**Ultra-high-net-worth individuals (3% billionaires)**	**Av. Deaths**	**ui_low**	**ui_high**	**Av. Deaths**	**ui_low**	**ui_high**
**2026**	2,334,440	1,172,736	3,448,504	1,264,689	894,971	1,641,817
**2027**	2,623,120	1,351,203	3,817,446	2,765,696	1,906,961	3,664,453
**2028**	2,857,100	1,508,740	4,129,336	3,216,905	2,284,367	4,200,948
**2029**	2,906,560	1,580,325	4,146,960	4,259,832	3,215,332	5,416,089
**2030**	2,914,771	1,635,615	4,105,996	4,366,822	3,320,979	5,525,594
**Total**	**13,635,991**	7,325,988	19,487,035	**15,873,944**	12,357,884	19,688,296

BAU= Business-as-usual; Av. Deaths = Averted Deaths in comparison to the Defunding scenario; ui_low = 95% Uncertainty Interval Lower Bound; ui_high = 95% Uncertainty Interval Upper Bound

WEB TABLE 32 Child main results by governance strata (averted deaths, by year and total), 2026-2030

	**Worst-off Governance**			**Better-off Governance**		
**BAU Scenario**	**Av. Deaths**	**ui_low**	**ui_high**	**Av. Deaths**	**ui_low**	**ui_high**
**2026**	82,653	0	161,860	76,523	24,422	128,620
**2027**	113,508	35,200	185,567	88,639	41,927	133,694
**2028**	216,765	132,736	301,034	106,149	56,141	155,117
**2029**	227,894	142,057	312,343	129,362	82,564	176,423
**2030**	228,745	146,923	309,257	166,447	114,529	219,008
**Total**	**869,564**	496,774	1,227,047	**567,119**	337,395	790,820
**Crypto Tax (3% top 10,000 holders)**	**Av. Deaths**	**ui_low**	**ui_high**	**Av. Deaths**	**ui_low**	**ui_high**
**2026**	82,665	0	161,860	76,522	24,422	128,620
**2027**	113,513	35,200	185,567	88,637	41,927	133,694
**2028**	216,763	132,736	301,034	106,149	56,141	155,117
**2029**	182,879	108,639	262,060	118,955	75,748	162,305
**2030**	186,765	120,476	258,257	140,063	100,348	180,510
**Total**	**782,585**	468,132	1,079,594	**530,326**	320,261	740,523
**Interest Earnings Debt Tax (100% of interest payments by LMICs to official creditors)**	**Av. Deaths**	**ui_low**	**ui_high**	**Av. Deaths**	**ui_low**	**ui_high**
**2026**	43,027	0	205,527	133,496	63,805	202,790
**2027**	138,794	0	338,335	164,759	85,340	245,695

**2028**	240,963	32,445	443,521	179,400	101,123	258,735
**2029**	251,085	49,416	442,876	199,856	125,414	275,928
**2030**	199,234	7,876	373,023	234,288	161,981	309,779
**Total**	**873,103**	0	1,766,238	**911,800**	545,854	1,281,140
**Ultra-high-net-worth individuals (1% billionaires)**	**Av. Deaths**	**ui_low**	**ui_high**	**Av. Deaths**	**ui_low**	**ui_high**
**2026**	60,625	0	241,925	149,336	74,969	222,441
**2027**	178,557	0	387,070	193,097	113,045	275,690
**2028**	281,224	57,147	491,474	208,957	126,657	293,724
**2029**	287,537	68,680	488,813	228,295	150,562	307,613
**2030**	285,836	79,834	478,287	236,401	163,439	314,511
**Total**	**1,093,779**	42,962	2,055,220	**1,016,086**	627,150	1,403,574
**Crypto Tax IMF2023**	**Av. Deaths**	**ui_low**	**ui_high**	**Av. Deaths**	**ui_low**	**ui_high**
**2026**	122,130	0	297,152	149,618	74,922	223,010
**2027**	180,979	0	395,292	195,469	110,798	282,900
**2028**	281,350	54,568	494,391	208,957	126,657	293,724
**2029**	289,759	70,826	496,969	228,294	150,562	307,613
**2030**	287,844	79,832	481,997	261,656	183,802	341,739
**Total**	**1,162,063**	74,456	2,130,362	**1,043,993**	651,384	1,437,602
**Carbon Tax**	**Av. Deaths**	**ui_low**	**ui_high**	**Av. Deaths**	**ui_low**	**ui_high**
**2026**	122,130	0	297,152	155,629	74,331	238,283
**2027**	180,979	0	395,292	195,466	110,798	282,900
**2028**	281,350	54,568	494,391	208,959	126,657	293,724
**2029**	289,759	70,826	496,969	228,296	150,562	307,613
**2030**	287,962	76,280	482,735	261,658	183,802	341,739
**Total**	**1,162,180**	66,550	2,146,394	**1,050,008**	649,170	1,455,130
**Interest Earnings Debt Tax (50% of interest payments by LMICs to official creditors + 25% of interest payments by LMICs to private lenders)**	**Av. Deaths**	**ui_low**	**ui_high**	**Av. Deaths**	**ui_low**	**ui_high**
**2026**	109,053	0	300,488	155,629	74,331	238,283
**2027**	199,688	0	421,428	201,257	116,102	287,684
**2028**	304,527	54,069	534,743	218,824	135,243	304,054
**2029**	311,951	72,297	534,387	237,789	157,407	318,896
**2030**	309,210	75,825	522,553	270,794	192,543	351,309
**Total**	**1,234,430**	48,688	2,312,391	**1,084,294**	682,764	1,494,553
**Corporate Tax**	**Av. Deaths**	**ui_low**	**ui_high**	**Av. Deaths**	**ui_low**	**ui_high**
**2026**	153,103	0	364,518	187,542	100,043	276,874
**2027**	164,931	0	467,922	225,875	135,954	317,286
**2028**	258,908	0	566,820	251,487	155,574	350,070
**2029**	263,670	0	566,259	269,219	177,486	363,991
**2030**	262,982	0	551,448	301,040	210,248	395,679
**Total**	**1,103,594**	0	2,526,777	**1,235,162**	791,423	1,700,031
**Ultra-high-net-worth individuals (2% billionaires)**	**Av. Deaths**	**ui_low**	**ui_high**	**Av. Deaths**	**ui_low**	**ui_high**

**2026**	172,638	0	392,840	193,561	106,773	283,670
**2027**	157,541	0	472,113	225,873	135,736	317,286
**2028**	260,040	0	580,236	251,487	155,574	350,070
**2029**	269,353	0	573,328	435,504	325,323	566,505
**2030**	268,426	0	556,474	461,058	352,350	590,029
**Total**	**1,127,997**	0	2,572,796	**1,567,482**	1,086,349	2,084,614
**Tobin Tax**	**Av. Deaths**	**ui_low**	**ui_high**	**Av. Deaths**	**ui_low**	**ui_high**
**2026**	185,894	0	408,378	198,193	110,081	289,495
**2027**	157,541	0	472,113	239,651	143,958	340,318
**2028**	260,040	0	580,236	424,278	299,251	561,910
**2029**	269,353	0	573,328	435,503	325,323	566,505
**2030**	268,426	0	556,474	461,059	352,350	590,029
**Total**	**1,141,254**	0	2,584,322	**1,758,684**	1,238,683	2,321,162
**Ultra-high-net-worth individuals (3% billionaires)**	**Av. Deaths**	**ui_low**	**ui_high**	**Av. Deaths**	**ui_low**	**ui_high**
**2026**	137,247	0	436,800	216,808	126,653	307,619
**2027**	158,737	0	483,330	419,207	291,525	560,497
**2028**	257,701	0	579,516	517,047	383,218	683,010
**2029**	267,119	0	575,629	602,604	446,032	778,588
**2030**	266,278	0	558,665	621,860	466,112	796,668
**Total**	**1,087,081**	0	2,644,204	**2,377,526**	1,770,899	3,072,251

BAU= Business-as-usual; Av. Deaths = Averted Deaths in comparison to the Defunding scenario; ui_low = 95% Uncertainty Interval Lower Bound; ui_high = 95% Uncertainty Interval Upper Bound

13.SUB-GROUP ANALYSIS FOR SUB-SAHARAN COUNTRIES

WEB TABLE 33 Main results stratifications only for 32 Sub-Saharan Countries (averted deaths, by year and total), 2026-2030

	**Overall (ASMR)**			**Under-5 children**		
**BAU Scenario**	**Av. Deaths**	**ui_low**	**ui_high**	**Av. Deaths**	**ui_low**	**ui_high**
**2026**	766,373	307,799	1,248,009	118,474	0	257,581
**2027**	973,367	599,247	1,365,687	168,100	47,063	284,038
**2028**	1,278,286	891,958	1,675,037	277,725	157,404	391,697
**2029**	1,337,928	922,054	1,771,115	307,532	184,135	426,910
**2030**	1,449,066	1,017,057	1,905,511	335,007	211,178	454,910
**Total**	**5,805,020**	3,867,280	7,738,796	**1,206,837**	608,224	1,747,232
**Crypto Tax (3% top 10,000 holders)**	**Av. Deaths**	**ui_low**	**ui_high**	**Av. Deaths**	**ui_low**	**ui_high**
**2026**	766,364	307,799	1,248,009	118,478	0	257,581
**2027**	973,359	599,247	1,365,687	168,102	47,063	284,038
**2028**	1,278,290	891,958	1,675,037	277,723	157,404	391,697
**2029**	911,067	551,350	1,252,184	251,318	142,001	363,403
**2030**	934,797	596,679	1,257,870	270,353	175,167	370,284
**Total**	**4,863,878**	3,277,695	6,481,422	**1,085,974**	583,004	1,557,005

Interest Earnings Debt Tax (100% of interest payments by LMICs to official creditors)	Av. Deaths	ui_low	ui_high	Av. Deaths	ui_low	ui_high
**2026**	888,911	255,140	1,555,026	85,969	0	299,128
**2027**	1,564,141	863,441	2,242,218	190,636	0	406,163
**2028**	1,856,773	1,133,761	2,536,686	299,483	70,907	516,275
**2029**	1,904,349	1,168,576	2,604,437	328,556	103,572	542,650
**2030**	1,582,503	911,690	2,220,450	303,584	96,389	502,620
**Total**	**7,796,678**	4,383,601	10,998,644	**1,208,230**	100,833	2,237,708
**Ultra-high-net-worth individuals (1% billionaires)**	**Av. Deaths**	**ui_low**	**ui_high**	**Av. Deaths**	**ui_low**	**ui_high**
**2026**	907,191	269,263	1,578,945	90,038	0	305,204
**2027**	1,564,135	863,441	2,245,740	190,639	0	406,163
**2028**	1,867,185	1,141,493	2,550,432	301,668	72,949	518,512
**2029**	1,904,351	1,168,576	2,604,437	328,555	103,572	542,650
**2030**	2,003,722	1,258,602	2,696,463	355,286	143,200	562,400
**Total**	**8,246,583**	4,894,587	11,485,170	**1,266,186**	157,725	2,296,263
Crypto Tax IMF2023	**Av. Deaths**	**ui_low**	**ui_high**	**Av. Deaths**	**ui_low**	**ui_high**
**2026**	1,367,085	763,839	1,968,717	151,543	0	355,863
**2027**	1,746,350	1,036,803	2,503,351	193,053	0	424,180
**2028**	2,035,036	1,321,778	2,766,575	301,794	68,178	523,044
**2029**	2,078,732	1,347,297	2,807,189	330,767	106,321	551,450
**2030**	2,013,679	1,265,263	2,709,331	357,288	144,926	565,409
**Total**	**9,240,882**	5,849,502	12,612,770	**1,334,446**	201,016	2,379,618
Carbon Tax	**Av. Deaths**	**ui_low**	**ui_high**	**Av. Deaths**	**ui_low**	**ui_high**
**2026**	1,376,488	747,749	2,007,834	154,887	0	362,204
**2027**	1,746,350	1,036,803	2,503,351	193,053	0	424,180
**2028**	2,035,036	1,321,778	2,766,575	301,795	68,178	523,044
**2029**	2,078,732	1,347,297	2,807,189	330,766	106,321	551,450
**2030**	2,174,318	1,448,889	2,883,498	357,404	136,038	569,442
**Total**	**9,410,924**	5,924,042	12,858,119	**1,337,906**	190,718	2,399,348
**Interest Earnings Debt Tax (50% of interest payments by LMICs to official creditors + 25% of interest payments by LMICs to private lenders)**	**Av. Deaths**	**ui_low**	**ui_high**	**Av. Deaths**	**ui_low**	**ui_high**
**2026**	1,369,675	675,609	2,068,741	141,812	0	375,982
**2027**	1,746,350	1,036,803	2,503,351	193,055	0	424,180
**2028**	2,080,234	1,366,858	2,797,230	313,952	79,323	536,763
**2029**	2,119,080	1,374,866	2,852,698	342,404	117,865	563,595
**2030**	2,213,785	1,478,450	2,933,933	368,550	147,048	581,569
**Total**	**9,529,124**	6,003,137	13,055,895	**1,359,773**	197,965	2,433,461
Corporate Tax	**Av. Deaths**	**ui_low**	**ui_high**	**Av. Deaths**	**ui_low**	**ui_high**
**2026**	1,555,935	821,071	2,352,198	144,331	0	388,256
**2027**	1,779,686	739,929	2,772,860	181,535	0	446,947
**2028**	2,067,643	1,055,486	3,015,358	290,768	13,723	552,776
**2029**	2,108,097	1,085,718	3,066,419	315,611	41,073	578,273

**2030**	2,203,040	1,208,961	3,137,793	342,891	75,988	597,579
**Total**	**9,714,402**	5,020,469	14,195,712	**1,275,136**	0	2,522,551
**Ultra-high-net-worth individuals (2% billionaires)**	**Av. Deaths**	**ui_low**	**ui_high**	**Av. Deaths**	**ui_low**	**ui_high**
**2026**	1,555,935	821,071	2,352,198	144,330	0	388,256
**2027**	1,779,686	739,929	2,772,860	181,539	0	446,947
**2028**	2,065,039	1,044,264	3,038,797	285,965	372	556,660
**2029**	2,108,097	1,085,718	3,066,419	315,611	41,073	578,273
**2030**	2,203,040	1,208,961	3,137,793	342,892	75,988	597,579
**Total**	**9,711,798**	4,991,840	14,213,301	**1,270,337**	0	2,525,564
**Tobin Tax**	**Av. Deaths**	**ui_low**	**ui_high**	**Av. Deaths**	**ui_low**	**ui_high**
**2026**	1,599,024	866,134	2,385,519	157,585	0	401,373
**2027**	1,779,686	739,929	2,772,860	181,537	0	446,947
**2028**	2,065,039	1,044,264	3,038,797	285,966	372	556,660
**2029**	2,108,097	1,085,718	3,066,419	315,611	41,073	578,273
**2030**	2,203,040	1,208,961	3,137,793	342,893	75,988	597,579
**Total**	**9,754,886**	5,039,931	14,251,144	**1,283,592**	0	2,538,886
**Ultra-high-net-worth individuals (3% billionaires)**	**Av. Deaths**	**ui_low**	**ui_high**	**Av. Deaths**	**ui_low**	**ui_high**
**2026**	1,574,271	561,596	2,589,170	133,205	0	413,844
**2027**	1,777,025	728,328	2,788,300	176,522	0	447,665
**2028**	2,065,040	1,044,264	3,038,797	285,964	372	556,660
**2029**	2,108,096	1,085,718	3,066,419	315,613	41,073	578,273
**2030**	2,203,039	1,208,961	3,137,793	342,891	75,988	597,579
**Total**	**9,727,470**	4,721,088	14,479,589	**1,254,195**	0	2,563,610

BAU= Business-as-usual; ASMR= Age standardized mortality rate; Av. Deaths = Averted Deaths in comparison to the Defunding scenario; ui_low = 95% Uncertainty Interval Lower Bound; ui_high = 95% Uncertainty Interval Upper Bound

14. FORECASTING RESULTS WITHOUT GOVERNANCE ADJUSTEMENT

In WEB TABLE 34 it can be appreciated the forecasting main results (i.e., with governance adjustment) and alternative results without governance adjustments. These results were generated based on the rate ratios of the main regression models, for overall and child age groups.

WEB TABLE 34 Forecasting results comparison with non-adjusted by governance forecasted results

Funding Sources	Business-as-usual		Crypto Tax (3% top 10,000 holders)		Interest Earnings Debt Tax (100% of interest payments by LMICs to official creditors)		Ultra-high-net-worth individuals (1% billionaires)		Crypto Tax IMF2023		Carbon Tax	
			32.6 billion U$D		74 billion U$D		90 billion U$D		100 billion U$D		102.2 billion U$D	
	Overall	Child (<5)	Overall	Child (<5)	Overall	Child (<5)	Overall	Child (<5)	Overall	Child (<5)	Overall	Child (<5)
Non-adjusted by governance	7,912,797	1,646,933	6,855,343	1,486,094	15,379,761	2,785,684	17,632,232	3,214,266	18,466,504	3,310,638	18,628,096	3,328,676
	(4,513,583 - 10,839,335)	(664,735 - 2,590,408)	(4,008,264 - 9,349,488)	(643,528 - 2,290,151)	(7,040,965 - 22,939,248)	(193,115 - 4,936,986)	(8,523,192 - 25,804,634)	(449,597 - 5,541,587)	(9,326,074 - 26,857,594)	(498,697 - 5,648,715)	(9,362,991 - 27,170,901)	(464,472 - 5,714,106)
Adjusted by governance	7,558,423	1,436,684	6,591,984	1,312,912	12,822,756	1,784,903	15,088,176	2,109,865	16,430,694	2,206,056	16,615,532	2,212,188
	(5,509,244 - 9,653,447)	(842,306-1,981,779)	(4,884,075 - 8,374,138)	(802,926 - 1,785,121)	(8,424,196 - 17,095,511)	(470,332 - 2,998,117)	(10,032,879 - 19,858,520)	(707,781 - 3,397,793)	(11,320,584 - 21,299,209)	(784,579 - 3,553,582)	(11,538,934 - 21,485,559)	(774,614 - 3,580,811)

Funding Sources	Business-as-usual		Interest Earnings Debt Tax (50% of interest payments by LMICs to official creditors + 25% of interest payments by LMICs to private lenders)		Corporate Tax		Ultra-high-net-worth individuals (2% billionaires)		Tobin Tax		Ultra-high-net-worth individuals (3% billionaires)	
			120 billion U$D		173.5 billion U$D		217.5 billion U$D		234 billion U$D		343.5 billion U$D	
	Overall	Child (<5)	Overall	Child (<5)	Overall	Child (<5)	Overall	Child (<5)	Overall	Child (<5)	Overall	Child (<5)
Non-adjusted by governance	7,912,797	1,646,933	20,352,322	3,588,022	23,943,484	4,058,338	26,890,627	4,295,478	28,324,448	4,398,485	35,331,646	5,039,120
	(4,513,583 - 10,839,335)	(664,735 - 2,590,408)	(10,553,285 - 29,481,578)	(608,486 - 6,014,678)	(10,215,117 - 36,496,519)	(235,266 - 7,218,137)	(12,472,554 - 39,893,729)	(414,364 - 7,540,144)	(13,551,590 - 41,846,961)	(483,386 - 7,723,134)	(19,197,619 - 50,398,045)	(826,027 - 8,679,205)
Adjusted by governance	7,558,423	1,436,684	17,709,318	2,318,724	20,131,999	2,338,756	22,963,571	2,695,480	24,526,199	2,899,938	29,509,934	3,464,607
	(5,509,244 - 9,653,447)	(842,306-1,981,779)	(12,351,645 - 22,871,053)	(778,272 - 3,758,437)	(12,457,715 - 27,510,726)	(357,598 - 4,210,899)	(14,944,443 - 30,871,693)	(673,378 - 4,610,691)	(16,268,726 - 32,604,183)	(850,725 - 4,836,305)	(20,273,842 - 38,603,961)	(1,235,930 - 5,609,331)

Data are averted deaths (95% Uncertainty Intervals) in comparison to the current defunding scenario.

15. MAIN LIMITATIONS

The analysis conducted in this forecast was based on the international guidelines for modeling and reporting set by ISPOR-SMDM. Despite that, this study is subject to limitations. Our estimates were based on levels of funding values by country, which restricts the sensitivity of the outcomes to minor changes in funding values, depending on whether the change in funding is sufficient to change the country's level of funding. In addition, the effects of cutting off aid from countries could vary widely depending on specific factors in each country. Furthermore, because we lack clear information about how and where these funding cuts will occur, it is difficult to predict exactly where the impacts will be felt and how severe they will be. This could lead to an underestimation of the total number of deaths, as the broader structural effects on countries might extend beyond the immediate financial impacts. The indirect consequences of dismantling the ODA could also have a wider impact on health system infrastructure, potentially hindering progress on several agendas that are not addressed in this analysis.

16. FORECASTING TRIANGULATION

In addition to the forecasting microsimulation method, we generated a forecasting triangulation analysis using an algebraic country-specific simulations method (ACSSM).

The analysis consisted of computing the averted deaths of the different analyzed scenarios in the main forecasting analysis in comparison with the Defunding scenario. For this, for each country -year we calculated:

Sc. Averted Deaths = Defunding Sc. Deaths – Sc. Deaths

Being,

Sc. = the scenario being analyzed.

R = Rate Ratio

Defunding Sc. Deaths = 2021 Deaths * (Defunding Sc. RR / 2023 RR)

Sc. Deaths = 2021 Deaths * (Sc. RR /2023 RR)[1]

We then aggregated the Sc. Averted Deaths by year, the total from 2026 to 2030. The results of these were extracted for Overall population and for the under-five children group.

Below it can be appreciated a table with the results, also including the microsimulation results. It can be appreciated that the algebraic country-specific simulations results fall within the microsimulation uncertainty intervals (UI) results.

WEB TABLE 35 Comparative risk assessment and microsimulation averted deaths results by scenario, for Overall and child age groups.

Scenario	Billion USD	Overall				Under-5			
		Microsimulation			ACSSM	Microsimulation			ACSSM
		Averted Deaths	Lower 95% UI	Upper 95% UI	Averted Deaths	Averted Deaths	Lower 95% UI	Upper 95% UI	Averted Deaths

[1] 2021 deaths were used instead of 2023 due to data availability limitations.

**Crypto Tax (3% top 10,000 holders)**	**32.58**	6,591,984	4,884,075	8,374,138	7,641,553	1,312,912	802,926	1,785,121	1,425,427
**Interest Earnings Debt Tax (100% of interest payments by LMICs to official creditors)**	**74**	12,822,756	8,424,196	17,095,511	14,778,517	1,784,903	470,332	2,998,117	2,191,715
**Ultra-high-net-worth individuals (1% billionaires)**	**90**	15,088,176	10,032,879	19,858,520	17,698,691	2,109,865	707,781	3,397,793	2,654,265
**Crypto Tax IMF2023**	**100**	16,430,694	11,320,584	21,299,209	18,865,953	2,206,056	784,579	3,553,582	2,832,858
**Carbon Tax**	**102.2**	16,615,532	11,538,934	21,485,559	19,023,997	2,212,188	774,614	3,580,811	2,838,605
**Interest Earnings Debt Tax (50% of interest payments by LMICs to official creditors + 25% of interest payments by LMICs to private lenders)**	**120**	17,709,318	12,351,645	22,871,053	20,693,058	2,318,724	778,272	3,758,437	2,900,381
**Corporate Tax**	**173.5**	20,131,999	12,457,715	27,510,726	23,710,814	2,338,756	357,598	4,210,899	2,769,228
**Ultra-high-net-worth individuals (2% billionaires)**	**217.5**	22,963,571	14,944,443	30,871,693	27,223,776	2,695,480	673,378	4,610,691	3,074,929
**Tobin Tax**	**234**	24,526,199	16,268,726	32,604,183	29,140,005	2,899,938	850,725	4,836,305	3,249,337
**Ultra-high-net-worth individuals (3% billionaires)**	**342.5**	29,509,934	20,273,842	38,603,961	35,254,605	3,464,607	1,235,930	5,609,331	3,706,508

REFERENCES

1. OECD. OECD Data Explorer: Creditor Reporting System [Internet]. Paris: OECD. Available from: https://data-explorer.oecd.org
2. Rasella D, Aquino R, Barreto ML. Impact of the Family Health Program on the quality of vital information and reduction of child unattended deaths in Brazil: an ecological longitudinal study. BMC Public Health. 2010;10:380.
3. Hone T, Rasella D, Barreto ML, Majeed A, Millett C. Association between expansion of primary healthcare and racial inequalities in mortality amenable to primary care in Brazil: a national longitudinal analysis. PLoS Med. 2017;14(5):e1002306.
4. Rasella D, Harhay MO, Pamponet ML, Aquino R, Barreto ML. Impact of primary health care on mortality from heart and cerebrovascular diseases in Brazil: a nationwide analysis of longitudinal data. BMJ. 2014;349:g4014.
5. Rasella D, Aquino R, Santos CAT, Paes-Sousa R, Barreto ML. Effect of a conditional cash transfer programme on childhood mortality: a nationwide analysis of Brazilian municipalities. Lancet. 2013;382(9886):57–64.
6. Aransiola TJ, Ordoñez JA, Cavalcanti DM, de Sampaio Morais GA, de Oliveira Ramos D, Rasella D. The combined effect of social pensions and cash transfers on child mortality: evaluating the last two decades in Brazil and projecting their mitigating effect during the global economic crisis. Lancet Reg Health Am. 2023;27:100618.
7. Rasella D, Alves FJO, Rebouças P, de Jesus GS, Barreto ML, Campello T, et al. Long-term impact of a conditional cash transfer programme on maternal mortality: a nationwide analysis of Brazilian longitudinal data. BMC Med. 2021;19(1):1–9.
8. Moncayo AL, Cavalcanti DM, Ordoñez JA, Almeida C, Perdomo JF, Zuluaga D, et al. Can primary health care mitigate the effects of economic crises on child health in Latin America? An integrated multicountry evaluation and forecasting analysis. Lancet Glob Health. 2024;12(6):e938–46.
9. de Sampaio Morais GA, Magno L, Silva AF, Guimarães NS, Ordoñez JA, Souza LE, et al. Effect of a conditional cash transfer programme on AIDS incidence, hospitalisations, and mortality in Brazil: a longitudinal ecological study. Lancet HIV. 2022;9(10):e690–9.
10. Cavalcanti DM, Ordoñez JA, Aransiola T, Almeida C, Díaz JFP, Mayorga DZ, et al. Evaluation and forecasting analysis of the association of conditional cash transfer with child mortality in Latin America, 2000-2030. JAMA Netw Open. 2023;6(7):e2323489.
11. Aransiola TJ, Cavalcanti D, Ordoñez JA, Hessel P, Moncayo AL, Chivardi C, et al. Current and projected mortality and hospitalization rates associated with conditional cash transfer, social pension, and primary health care programs in Brazil, 2000-2030. JAMA Netw Open. 2024;7(4):e247519.
12. Sibils GB, Brachowicz N, Silva NJ, Landin E, Macicame I, Naidoo M, et al. The mitigating effect of social protection on undernourishment during economic downturns: a longitudinal study of 46 low- and middle-income countries over the last two decades. Soc Sci Med. 2024;361:117365.

13. Basterra EL, Gentilini U, Cavalcanti DM, da Silva AF, de Sales L de OF, Silva NJ, et al. Impact of social protection on child malnutrition and mortality across 46 LMICs: a longitudinal study over two decades with insights from the COVID-19 pandemic. EClinicalMedicine. 2025;87:103378.

14. Höfler M. The Bradford Hill considerations on causality: a counterfactual perspective. Emerg Themes Epidemiol. 2005;2:11.

15. KFF. The Outlook for PEPFAR in 2025 and Beyond [Internet]. San Francisco: KFF; 2025. Available from: https://www.kff.org/global-health-policy/the-outlook-for-pepfar-in-2025-and-beyond/

16. USAID. The President's Malaria Initiative - 18th Annual Report to Congress [Internet]. Washington, DC: USAID; 2024 [cited 2026 Aug 6]. Available from: https://reliefweb.int/report/democratic-republic-congo/presidents-malaria-initiative-18th-annual-report-congress-april-2024

17. Symons TL, Lubinda J, McPhail M, Saddler A, van den Berg M, Baggen H, et al. Estimating the potential malaria morbidity and mortality avertable by the President's Malaria Initiative in 2025: a geospatial modelling analysis. medRxiv [Preprint]. 2025.

18. Stover J, Sonneveldt E, Tam Y, Clark RA, Phillips AN, Smith J, et al. Effects of reductions in US foreign assistance on HIV, tuberculosis, family planning, and maternal and child health: a modelling study. Lancet Glob Health. 2025;13(10):e1669–80.

19. Nichols B, Moakley E. Impact Dashboard - Impact Counter [Internet]. Boston: Impact Counter; 2025 [cited 2026 Aug 6]. Available from: https://www.impactcounter.com/

20. Ahsan KZ, Angeles G, Choudhury A, Singh K, Taylor TM, Majid F, et al. Accounting for aid: estimating the impact of United States' global health investments on mortality among women of reproductive age using synthetic control and Bayesian methods. J Glob Health. 2025;15:4067.

21. Ten Brink D, Martin-Hughes R, Bowring AL, Wulan N, Burke K, Tidhar T, et al. Impact of an international HIV funding crisis on HIV infections and mortality in low-income and middle-income countries: a modelling study. Lancet HIV. 2025;12(5):e346–54.

22. Kenny C, Sandefur J. How Many Lives Does US Foreign Aid Save? [Internet]. Washington, DC: Center for Global Development; 2025 [cited 2026 Aug 6]. Available from: https://www.cgdev.org/blog/how-many-lives-does-us-foreign-aid-save

23. Weiss W, Piya B, Andrus A, Ahsan KZ, Cohen R. Estimating the impact of donor programs on child mortality in low- and middle-income countries: a synthetic control analysis of child health programs funded by the United States Agency for International Development. Popul Health Metr. 2022;20(1):2.

24. Kates J, Rouw A, Oum S, Wexler A. How Much Global Health Funding Goes Through USAID? [Internet]. San Francisco: KFF; 2025 [cited 2026 Aug 6]. Available from: https://www.kff.org/global-health-policy/how-much-global-health-funding-goes-through-usaid/

25. Raymaekers K, Luyckx K, Moons P. A guide to improve your causal inferences from observational data. Eur J Cardiovasc Nurs. 2020;19(8):757–62.

26. Rajkumar AS, Swaroop V. Public spending and outcomes: does governance matter? J Dev Econ. 2008;86(1):96–111.

27. Kabongo WNS, Mbonigaba J. Effectiveness of public health spending: investigating the moderating role of governance using partial least squares structural equation modelling (PLS-SEM). Health Res Policy Syst. 2024;22(1):80.

28. Doucouliagos C, Hennessy J, Mallick D. Health aid, governance and infant mortality. J R Stat Soc Ser A Stat Soc. 2021;184(2):761–83.

29. World Bank. Worldwide Governance Indicators [Internet]. Washington, DC: World Bank; Available from: https://www.worldbank.org/en/publication/worldwide-governance-indicators

30. Gramacy RB. tgp: an R package for Bayesian nonstationary, semiparametric nonlinear regression and design by treed Gaussian process models. J Stat Softw. 2007;19(9):1–46.

31. Thiele JC, Kurth W, Grimm V. Facilitating parameter estimation and sensitivity analysis of agent-based models: a cookbook using NetLogo and R. J Artif Soc Soc Simul. 2014;17(3):11.

32. Cavalcanti DM, de Sales L de OF, da Silva AF, Basterra EL, Pena D, Monti C, et al. Evaluating the impact of two decades of USAID interventions and projecting the effects of defunding on mortality up to 2030: a retrospective impact evaluation and forecasting analysis. Lancet. 2025;406(10500):283–94.

33. Briggs AH, Weinstein MC, Fenwick EAL, Karnon J, Sculpher MJ, Paltiel AD. Model parameter estimation and uncertainty analysis: a report of the ISPOR-SMDM Modeling Good Research Practices Task Force Working Group-6. Med Decis Making. 2012;32(5):722–32.
